# Landscape of the complete RNA chemical modifications in the human 80S ribosome

**DOI:** 10.1093/nar/gky811

**Published:** 2018-09-07

**Authors:** Masato Taoka, Yuko Nobe, Yuka Yamaki, Ko Sato, Hideaki Ishikawa, Keiichi Izumikawa, Yoshio Yamauchi, Kouji Hirota, Hiroshi Nakayama, Nobuhiro Takahashi, Toshiaki Isobe

**Affiliations:** 1 Department of Chemistry, Graduate School of Science, Tokyo Metropolitan University, Minami-osawa 1-1, Hachioji-shi, Tokyo 192-0397, Japan; 2 Department of Applied Biological Science, Graduate School of Agriculture, Tokyo University of Agriculture and Technology, Saiwai-cho 3-5-8, Fuchu-shi, Tokyo 183-8509, Japan; 3 Biomolecular Characterization Unit, RIKEN Center for Sustainable Resource Science, 2-1 Hirosawa, Wako, Saitama 351-0198, Japan

## Abstract

During ribosome biogenesis, ribosomal RNAs acquire various chemical modifications that ensure the fidelity of translation, and dysregulation of the modification processes can cause proteome changes as observed in cancer and inherited human disorders. Here, we report the complete chemical modifications of all RNAs of the human 80S ribosome as determined with quantitative mass spectrometry. We assigned 228 sites with 14 different post-transcriptional modifications, most of which are located in functional regions of the ribosome. All modifications detected are typical of eukaryotic ribosomal RNAs, and no human-specific modifications were observed, in contrast to a recently reported cryo-electron microscopy analysis. While human ribosomal RNAs appeared to have little polymorphism regarding the post-transcriptional modifications, we found that pseudouridylation at two specific sites in 28S ribosomal RNA are significantly reduced in ribosomes of patients with familial dyskeratosis congenita, a genetic disease caused by a point mutation in the pseudouridine synthase gene *DKC1*. The landscape of the entire epitranscriptomic ribosomal RNA modifications provides a firm basis for understanding ribosome function and dysfunction associated with human disease.

## INTRODUCTION

Human ribosomes are comprised of two ribonucleoprotein subunits: the small subunit (40S) containing one ribosomal RNA (rRNA; 18S) and 33 ribosomal proteins, and the large subunit (60S) containing three rRNAs (5S, 5.8S, 28S) and 47 ribosomal proteins (1). In each subunit, rRNAs form the catalytic core for translation, and ribosomal proteins appear to stabilize and fine-tune ribosomal structure and function (2,3). Human ribosomes harbor numerous post-transcriptional modifications (PTMs), such as methylation or acetylation of the nucleotide base or the 2′-hydroxyl group of the ribose moiety, or conversion of uridine to pseudouridine (Ψ) (4–6). These PTMs are fundamental to global ribosome topology and functions (decoding, peptidyl transfer, translocation) and stabilize the structure either by influencing the local charge environment or increasing the hydrogen-bonding capability (7–10). In addition, the PTMs optimize the interaction of ribosomes with tRNAs, mRNAs and translation factors (9,11). Therefore, any alteration of rRNA PTMs can negatively impact both translation rate and accuracy (12–14) and impair responses to metabolites and antibiotics (15). Moreover, certain PTMs are associated with altered translation in various diseases, such as cancer and infectious diseases (16–18). Therefore, the complete identification of the PTMs and their sites in human ribosomes would constitute a major advance for understanding ribosome-associated cellular physiology and pathology.

Recently, using cryo-electron microscopy (cryo-EM), >130 individual rRNA modifications were visualized on the three-dimensional (3D) structure of the human 80S ribosome, and many of these PTMs were posited to be human-specific modifications (19). A large proportion of these PTMs, however, is inconsistent with those reported previously (4,5,20,21 and reference therein) in terms of both the types of chemical groups and sites of modification in human rRNA. Using a method for quantitative RNA analysis based on mass spectrometry (MS) technology termed SILNAS (stable isotope–labeled ribonucleic acid as an internal standard), we, for the first time, mapped all PTMs of the 80S ribosome from two eukaryotes, namely the fission yeast *Schizosaccharomyces pombe* (22) and the budding yeast *Saccharomyces cerevisiae* (23). Then, as a part of a cryo-EM analysis, we determined the PTMs in the *Leishmania* ribosome that are involved in binding the aminoglycoside paromomycin (24). We, therefore, took advantage of this technology to determine all PTMs in the entire rRNAs of the human 80S ribosome. We further investigated how human disease might alter the landscape of rRNA PTMs by assessing the PTMs of rRNAs of the 80S ribosome from patients with familial dyskeratosis congenita (DC) caused by mutations in the gene *DKC1*, the product of which converts uridine to Ψ.

## MATERIALS AND METHODS

### Reagents

Standard laboratory chemicals were obtained from Wako Pure Chemical Industries. Sodium guanosine-^13^C_10_ 5′-triphosphate (98 atom% ^13^C) and RNase A were obtained from Sigma-Aldrich. Sodium cytidine-^13^C_9_ 5′-triphosphate and sodium uridine-^13^C_9_ 5′-triphosphate (98 atom% ^13^C) were purchased from Santa Cruz Biotechnology. 5,6-D2-uridine was obtained from C/D/N Isotopes. RNase T1 was purchased from Worthington and further purified by reversed-phase LC before use. Triethylammonium acetate buffer (pH 7.0) was purchased from Glen Research. Chemically synthesized oligonucleotides were obtained from JBioS (the oligonucleotide sequences are given in Supplementary Table S1).

### Cells and culture conditions

TK6 cell (human lymphoblast cell from spleen, Epstein-Barr virus transformed) and HeLa cell (human epithelial cell from cervix, adenocarcinoma) were obtained from the American Type Culture Collection. TK6 and HeLa cells were cultured at 37°C as described (25,26). Media used in this study are listed in Supplementary Table S2. To obtain U/C-5,6-D2-labeled rRNAs, TK6 cells deficient in *UMPS* were cultured in the U/C-5,6-D2 labeling medium containing 330 μM 5,6-D2-uridine instead of uridine with natural isotope distribution.

DC fibroblasts, lymphoblastoid cell line (LCL), and control cells were obtained from Coriell Cell Repositories (GM01774, GM01786, GM01787, AG04645, GM03194, GM03195, GM03650 and AG03738, https://www.coriell.org/) or the repositories of the National Institutes of Biomedical Innovation, Health and Nutrition (KURB1983, http://cellbank.nibiohn.go.jp/). Cells were maintained in DMEM with 20% fetal bovine serum or RPMI 1640 with 2 mM l-glutamine and 15% fetal bovine serum. Information concerning the pathology associated with these cells is summarized in Supplementary Table S3.

### Generation of *UMPS* deficient TK6 cells

To confirm the site of pseudouridylation, TK6 cells deficient in uridine monophosphate synthetase were generated to block *de novo* synthesis of uridine and grown in culture medium containing 5,6-D2-uridine (Supplementary Table S2) to differentiate the molecular mass of uridine from that of Ψ. The method used was CRISPR/Cas9 gene editing technology (27) targeting human *UMPS* gene (Gene ID: 7372, https://www.ncbi.nlm.nih.gov/gene/7372), and proper gene-targeting events with *UMPS* were confirmed by PCR using the genome DNA as a template. The details will be described elsewhere.

### Preparation of cellular RNA

Total RNA (20 μg) was prepared from 10 ml TK6 cell culture (∼1.0 × 10^6^ cell/ml) using Sepasol-RNA I Super G (Nacalai Tesque, Kyoto, Japan). rRNAs were purified from the total cellular RNA (∼15 μg) by reversed-phase LC on a PLRP-S 300Å column (2.1 × 100 mm, 3 μm, Agilent Technologies) or 4000 Å column (4.6 × 150 mm, 10 μm, Agilent Technologies) (28). A rRNA preparation of >95% purity was used for this study.

### *In vitro* transcription of internal standard RNAs for SILNAS

To construct the plasmids for *in vitro* transcription of internal standard RNAs, DNAs encoding human 5S, 5.8S and 18S rRNAs were amplified by PCR from genomic DNA (PCR primers are noted in Supplementary Table S1). The amplified DNAs for 5S and 5.8S rRNAs were inserted into the EcoRI/XhoI sites of plasmid pBluescript II KS(+) (Agilent Technologies). For 18S rRNA, the amplified DNA was inserted into the KpnI/XhoI sites of plasmid pcDNA3.1(+) (Invitrogen, Carlsbad, CA, USA). For 28S rRNA, the 5′-terminal half of the PCR-amplified DNA (HindIII/BglII digest, ∼2.4 kb) and 3′-terminal half of the chemically synthesized DNA (BglII/XhoI digest, ∼2.7 kb, Wako Pure Chemical Industries) were ligated into pcDNA3.1(+) because we failed to amplify the 3′-terminal half of the DNA including the extremely GC-rich region. Before *in vitro* transcription, the plasmid was linearized with SpeI or XhoI to terminate the product at the end of the rRNA. To synthesize RNA, 2 μg of template DNA was incubated and transcribed using a Megascript T3 or T7 kit (Invitrogen). When RNA was synthesized, guanosine-^13^C_10_ 5′-triphosphate, cytidine-^13^C_9_ 5′-triphosphate, or uridine-^13^C_9_ 5′-triphosphate solution was used instead of the respective 5′-triphosphate reagent that contained carbons with a natural isotope distribution. The RNA was precipitated in ethanol, solubilized in nuclease-free water, and purified further by reversed-phase LC as described above (Supplementary Figure S1).

### Direct nanoflow LC-MS and tandem MS (MS/MS) analysis of RNA fragments

Nucleolytic RNA fragments were analyzed with a direct nanoflow LC-MS system as described (29). The LC eluate was sprayed online at –1.3 kV with the aid of a spray-assisting device (30) to a Q Exactive mass spectrometer (Thermo Fisher Scientific) in negative ion mode. Other settings were as described (23,31).

### Database search and interpretation of MS/MS RNA spectra

Ariadne (32) was used for database searches and assignment of MS/MS RNA spectra. We used the human genome database as a resource in conjunction with Ariadne (30). The following default search parameters for Ariadne were used: maximum number of missed cleavages, 1; variable modification parameters, two methylations per RNA fragment for any residue; RNA mass tolerance, ±5 ppm and MS/MS tolerance, ±20 ppm. For assignment of Ψ residues using 5,6-D2-uridine labeled RNAs, the mass table and the variable modification parameters were altered from default values to ‘5,6D_CU’ and ‘Ψ’, respectively, because both C and U were labeled with the medium and the pseudouridylation reaction results in the exchange of the position 5 deuterium of 5,6-D2-uridine to the proton of solvent, providing a −1 Da mass shift (33). The Ψ-characteristic signature ion at *m*/*z* 207.041 and 208.047 (for rRNAs containing natural and 6-D-labeled Ψ, respectively) was also used for assignment of Ψ (31).

### SILNAS-based quantitation of the stoichiometry of PTM

SILNAS-based quantitation was performed as described (23). In brief, RNA (∼100 fmol) from natural sources or cells grown in guanosine with natural isotope distribution was mixed with an equal amount of synthetic RNA transcribed *in vitro* with ^13^C-labeled guanosine. The 1:1 RNA mixing was performed based on the measurement of the absorbance at 260 nm and ensured later by a correction factor obtained experimentally by the quantitative analysis of ∼10 labeled and non-labeled RNA fragment pairs with known nucleotide sequences without modifications. The mix was digested with RNase T1 (∼4 ng/μl) in 100 mM triethylammonium acetate buffer (pH 7.0) at 37°C for 60 min. RNase A digested RNA fragments were produced by the same procedure using a reference RNA transcribed *in vitro* with ^13^C-labelled cytidine and uridine. The stoichiometry of RNA modification at each site was estimated by Ariadne program designed for SILNAS. In principle, the program first extracts the intensity of MS signals from raw data, assigns all pairs of light and heavy signal of RNA fragments produced by SILNAS, compares quantitatively the signal intensities of each pair, and determines the stoichiometry of modification as the ratio of the difference in signal intensities between the light (unmodified) and heavy (a sum of unmodified and modified) fragments to the signal intensity of the heavy isotope-labeled fragment. Upon calculation, Ariadne program considers the isotopical impurities in commercial rNTPs (∼2%) and corrects the estimate by a correction factor obtained experimentally as described above. Finally, the results were confirmed by manual inspection of the original MS spectrum to examine whether the estimates are based on ‘uncontaminated’ MS signals.

The stoichiometries of modifications in TK6 rRNAs were estimated with the fragments having unique nucleotide sequence to remove redundant sequence derived from multiple rRNA positions. To obtain such fragments that contain modified nucleoside(s) within the unique nucleotide sequence, we designed and performed systematic RNase H cleavage of full-length rRNA in many cases. The stoichiometries of rRNA modifications in DC-related cells, however, were estimated solely by the analysis of RNase T1 fragments without removing the redundancy of fragments. Thus, the modifications in the fragments with redundant nucleotide sequence were estimated with lower stoichiometries than those in the fragments with less redundant or unique nucleotide sequence. To evaluate the estimated stoichiometries of modification at each site, the numbers of redundant sequence are provided in Supplementary Tables S4-1, S4-2 and S4-3.

### Other procedures for RNA analysis

Sequence-specific RNase H cleavage of rRNAs was performed as described (22,23). All RNA/DNA sequences used for the cleavage are presented in Supplementary Table S1. The resulting RNase H fragments were separated by reversed-phase LC as described above for further digestion with other RNases. The masses of RNA fragments and a-, c-, w- and y-series ions were calculated with Ariadne (http://ariadne.riken.jp/). For the calculation of the C/U-5D-labeled fragment, 98 atom% deuterium was used. The tertiary structure of each rRNA, as defined by the PDB file, was analyzed with Swiss-Pdb viewer (http://spdbv.vital-it.ch/).

## RESULTS AND DISCUSSION

### Human rRNAs harbor 14 distinct types of PTMs at 228 internal sites

To determine the PTMs of human rRNAs in TK6 cells, we applied SILNAS (22) to 5S, 5.8S, 18S, and 28S rRNAs that had been purified by reversed-phase liquid chromatography (LC) (28). Each purified rRNA was mixed with an *in vitro* transcribed G-^13^C_10_-labeled (guanosine labeled with 10 ^13^C-atoms) reference RNA having the same sequence as each sample RNA; the mixture was digested with RNase T1 and analyzed with a nanoflow LC-coupled MS system equipped with Ariadne software to identify each RNA fragment and to detect modified oligonucleotides (for details, see Supplementary Figures S2 and S3). To align the RNase T1 fragments and identify all PTMs, we performed a similar analysis for RNase A digests of each rRNA with *in vitro* transcribed cytidine-^13^C_9_- and uridine-^13^C_9_-labeled reference RNAs; when the rRNA produced multiple fragments having the same nucleotide sequence, the undigested rRNA was systematically cleaved with RNase H (after annealing with a corresponding complementary DNA) to avoid the production of such fragments and to distinguish the redundant sequences prior to subsequent digestion with RNase T1 or A (Supplementary Figures S2 and S3). Thus, all RNA fragments were detected and quantitated in this study regardless of presence or absence of PTM. We also confirmed all pseudouridylation sites *via* analysis of rRNAs prepared from the *UMPS*-deficient TK6 cells that had been genetically modified using the CRISPR/Cas9 technique to block *de novo* synthesis of uridine and grown in culture medium containing 5,6-D2-uridine to differentiate the molecular mass of uridine from that of Ψ (Taoka *et al.*, manuscript in preparation). Thus, we could unequivocally align all the nucleolytic fragments covering the entire rRNA sequences and construct a complete PTM map of human rRNAs.

The SILNAS technology allows comprehensive quantitative identification of all RNA PTMs exceeding ∼5% in stoichiometry (22). Under the criteria, the human 80S ribosome was found to harbor 14 distinct types of PTMs at 228 internal sites (Table 1 and Supplementary Table S5): 4 in 5.8S (total length, 157 nt), 91 in 18S (1869 nt) and 133 in 28S (5064 nt). No PTMs were found in 5S rRNA (120 nt). Because PTMs of human rRNAs affect the structure, function, and biogenesis of ribosomes and because dysregulation of PTMs has been implicated in cancer and inherited human disorders, the analysis of rRNA PTMs has been the subject of numerous studies over decades. In fact, of the 228 PTM sites identified in this study, 218 were consistent with the results of previous studies (Supplementary Table S6 and references therein); however, we detected unmodified U at position 688 in 18S rRNA and at position 4501 in 28S rRNA instead of Ψ described in the snoRNABase site (4) (https://www-snorna.biotoul.fr/). The latter terminates the argument about the site of pseudouridylation, 4501 (34) or 4502 (35), by proving the sequence U4501-Ψ4502. Likewise, we found unmodified C at position 2279 in 28S rRNA instead of Cm (2′-*O*-methyl cytosine) described by Incarnato *et al.* (5) (Supplementary Figure S4). Moreover, we detected 10 additional PTM sites, comprising 3 ribose 2′-*O*-methylations and 7 pseudouridylations (Table 1 and Supplementary Figure S5), most of which are located in the functionally important interior region of the ribosome: Cm621, Ψ897, Ψ1045, Ψ1136, and Ψ1232 in 18S, and Um1760, Ψ1768, Ψ2619, Gm3606 and Ψ4463 in 28S. In particular, Cm621 in 18S is located at the decoding center. To provide the basis for these 2′-*O*-methylation/pseudouridylation reactions, we presented the candidate guide snoRNA responsible for modification at each site (Supplementary Figure S6), as predicted by Snoscan software (36) (http://lowelab.ucsc.edu/snoscan/) or the in-house program (23) using the snoRNA sequences compiled in snoRNA Atlas database (37) (http://snoatlas.bioinf.uni-leipzig.de/index.php).

**Table 1.** Position, type, and stoichiometry of modified nucleotides found in human rRNAs

rRNA	Modified nucleotide	Type^a^	Percent modification	rRNA	Modified nucleotide	Type^a^	Percent modification	rRNA	Modified nucleotide	Type^a^	Percent modification	rRNA	Modified nucleotide	Type^a^	Percent modification
5.8S	14	Um	5	18S	1004	Ψ	97	28S	1768	Ψ	100	28S	3866	Cm	99
5.8S	55	Ψ	60	18S	1031	Am	97	28S	1769	Ψ	100	28S	3878	Gm	98
5.8S	69	Ψ	61	18S	1045	Ψ	92	28S	1779	Ψ	100	28S	3899	Ψ	100
5.8S	75	Gm	87	18S	1046	Ψ	100	28S	1847	Ψ	95	28S	3904	Um	96
				18S	1056	Ψ	93	28S	1849	Ψ	95	28S	3923	Gm	80
18S	27	Am	100	18S	1081	Ψ	94	28S	1858	Am	96	28S	3938	Ψ	93
18S	34	Ψ	100	18S	1136	Ψ	7	28S	1868	Cm	35	28S	4020	Gm	83
18S	36	Ψ	82	18S	1174	Ψ	100	28S	2338	Cm	99	28S	4032	Cm	100
18S	93	Ψ	87	18S	1177	Ψ	100	28S	2350	Am	100	28S	4166	Gm	98
18S	99	Am	99	18S	1232	Ψ	98	28S	2351	Gm	100	28S	4190	m^6^A	100
18S	105	Ψ	99	18S	1238	Ψ	97	28S	2352	Cm	90	28S	4197	Um	97
18S	109	Ψ	99	18S	1244	Ψ	100	28S	2388	Am	73	28S	4198	Gm	92
18S	116	Um	98	18S	1248	m^1^acp^3^Ψ	100	28S	2402	Um	87	28S	4263	Ψ	98
18S	119	Ψ	94	18S	1272	Cm	47	28S	2409	Cm	98	28S	4266	Ψ	90
18S	121	Um	98	18S	1288	Um	98	28S	2411	Gm	90	28S	4269	Ψ	93
18S	159	Am	96	18S	1326	Um	100	28S	2495	Ψ	92	28S	4276	Um	88
18S	166	Am	100	18S	1328	Gm	100	28S	2619	Ψ	90	28S	4282	Ψ	83
18S	172	Um	96	18S	1337	ac^4^C	79	28S	2774	Am	84	28S	4323	Ψ	95
18S	174	Cm	92	18S	1347	Ψ	98	28S	2791	Cm	93	28S	4331	Ψ	93
18S	210	Ψ	83	18S	1367	Ψ	98	28S	2802	Am	92	28S	4340	Gm	99
18S	218	Ψ	100	18S	1383	Am	98	28S	2811	Cm	87	28S	4362	Gm	97
18S	296	Ψ	25	18S	1391	Cm	95	28S	2824	Um	99	28S	4373	Ψ	96
18S	354	Um	20	18S	1442	Um	78	28S	2826	Ψ	20	28S	4390	Ψ	99
18S	406	Ψ	87	18S	1445	Ψ	90	28S	2830	Ψ	9	28S	4393	Ψ	97
18S	428	Um	76	18S	1447	Gm	34	28S	2848	Cm	72	28S	4401	Ψ	89
18S	436	Gm	76	18S	1490	Gm	100	28S	2863	Gm	49	28S	4412	Ψ	100
18S	462	Cm	100	18S	1625	Ψ	79	28S	3606	Gm	96	28S	4417	m^5^C	100
18S	468	Am	99	18S	1639	m^7^G	100	28S	3616	Ψ	89	28S	4426	Cm	98
18S	484	Am	97	18S	1643	Ψ	96	28S	3618	Ψ	95	28S	4427	Ψ	98
18S	509	Gm	98	18S	1668	Um	8	28S	3674	Ψ	99	28S	4441	Ψ	87
18S	512	Am	83	18S	1678	Am	94	28S	3680	Cm	100	28S	4463	Ψ	17
18S	517	Cm	100	18S	1692	Ψ	98	28S	3694	Ψ	100	28S	4464	Gm	91
18S	572	Ψ	97	18S	1703	Cm	92	28S	3697	Am	88	28S	4468	Um	100
18S	576	Am	96	18S	1804	Um	86	28S	3703	Am	100	28S	4469	Gm	100
18S	590	Am	72	18S	1832	m^6^A	99	28S	3709	Ψ	72	28S	4470	Ψ	100
18S	601	Gm	89	18S	1842	ac^4^C	99	28S	3713	Ψ	98	28S	4491	Ψ	91
18S	609	Ψ	90	18S	1850	m^6^_2_A	94	28S	3723	Gm	83	28S	4493	Am	87
18S	621	Cm	62	18S	1851	m^6^_2_A	94	28S	3737	Ψ	85	28S	4500	m^3^U	120
18S	627	Um	99					28S	3739	Am	90	28S	4502	Ψ	100
18S	644	Gm	98	28S	389	Am	98	28S	3741	Ψ	100	28S	4506	Cm	100
18S	649	Ψ	93	28S	391	Am	98	28S	3743	Ψ	100	28S	4522	Ψ	98
18S	651	Ψ	93	28S	1303	Gm	71	28S	3747	Ψ	100	28S	4541	Am	43
18S	668	Am	99	28S	1309	m^1^A	100	28S	3749	Ψ	100	28S	4546	Ψ	100
18S	681	Ψ	67	28S	1310	Am	44	28S	3761	m^5^C	100	28S	4549	Ψ	100
18S	683	Gm	99	28S	1313	Am	100	28S	3764	Am	96	28S	4560	Am	37
18S	686	Ψ	95	28S	1327	Cm	92	28S	3771	Gm	100	28S	4588	Gm	75
18S	797	Cm	68	28S	1509	Gm	99	28S	3787	Cm	80	28S	4590	Um	82
18S	799	Um	98	28S	1511	Am	99	28S	3797	Ψm	100	28S	4593	Gm	100
18S	801	Ψ	100	28S	1521	Am	109	28S	3801	Ψ	50	28S	4598	Ψ	92
18S	814	Ψ	100	28S	1523	Ψ	88	28S	3804	Am	92	28S	4606	Ψ	42
18S	815	Ψ	100	28S	1569	Ψ	68	28S	3809	Am	100	28S	4607	Gm	100
18S	822	Ψ	99	28S	1612	Gm	100	28S	3820	Cm	100	28S	4643	Ψ	39
18S	863	Ψ	95	28S	1664	Ψ	97	28S	3823	Ψ	66	28S	4659	Ψ	87
18S	866	Ψ	88	28S	1670	Ψ	96	28S	3830	Ψ	92	28S	4937	Ψ	81
18S	867	Gm	28	28S	1731	Ψ	100	28S	3832	Ψ	100	28S	4966	Ψ	86
18S	897	Ψ	23	28S	1747	Gm	89	28S	3846	Am	43	28S	4975	Ψ	72
18S	918	Ψ	42	28S	1760	Um	70	28S	3848	Cm	67				
18S	966	Ψ	89	28S	1766	Ψ	40	28S	3863	Ψ	33				

^a^The abbreviations for nucleotides are as follows: Ψ, pseudouridine; Am, 2′-*O*-methyladenosine; Cm, 2′-*O*-methylcytidine; Gm, 2′-*O*-methylguanosine; Um, 2′-*O*-methyluridine; Ψm, 2′-*O*-methylpseudouridine; m^1^A, 1-methyladenosine; m^6^_2_A, N6, N6-dimethyladenosine; m^5^C, 5-methylcytidine; m^7^G, 7-methylguanosine; m^3^U, 3-methyluridine; m^6^A, N6-methyladenosine; ac^4^C, N4-acetylcytidine; m^1^acp^3^Ψ, 1-methyl-3-(3-amino-3-carboxypropyl)pseudouridine.

Underline denotes the modified nucleotides newly identified in this study. The standard deviation of the values described here is expected to be around 2%, deduced from the average value of the standard deviation in previous SILNAS-based PTM quantitation (Taoka M, *Nucleic Acids Res*. 2015; 43: e115).

One of the most significant findings of this study is that the rRNA PTMs detected in TK6 cells did not differ significantly from those identified in other human cells, *i.e*., HeLa cells, where we determined ∼70% of all PTMs in the rRNAs (Supplementary Table S5), and induced pluripotent stem cells (Taoka *et al.*, unpublished results), in terms of PTM types, sites, and the stoichiometry of modification at each site. This suggests that PTMs in human rRNAs have little polymorphism regarding the cell type. It should be also noted that human rRNAs contains many sequence variants (38). In fact, human TK6 cell used in this study contains five sequence variants including the one we used for the reference RNA. Although the variation includes base insertions, deletions and substitutions within ∼0.1% region of the whole sequence, all of the variation-containing RNase T1 fragments have been identified in this study and are found to contain no modified nucleosides (Supplementary Table S7). Thus, the variation in human rRNA sequence does not impact the current analysis.

Apart from the chemical analysis of PTMs of human rRNAs described above, Natchiar *et al.* recently reported the cryo-EM structure of the human 80S ribosome prepared from HeLa cell at ∼2.5 Å resolution (19). They visualized ∼136 PTMs among the rRNAs and claimed that there are many human-specific modifications that perhaps could inform the design of drugs that could selectively inhibit ribosome function in pathogens. The results of that cryo-EM study, however, are only partly consistent with those reported previously (4,5,20,21 and reference therein) and those we obtained during our MS-based analysis: namely, we estimated that only 78 of ∼136 PTMs (57%) visualized in the cryo-EM analysis of Natchiar *et al.* are supported by the MS analysis, whereas most others are found to be unmodified (Supplementary Table S8). Thus, the cryo-EM analysis of Natchiar *et al.* covered 34% of the total PTMs (78 of 228) identified in our MS-based analysis (Supplementary Table S8). Our analysis also revealed that all PTMs found in human rRNAs are typical of eukaryotic rRNAs, *i.e*., no human-specific PTMs were detected. Given that the results of the SILNAS-based PTM analysis of rRNAs in HeLa cell covering ∼70% of all PTMs showed that the modified nucleosides locate exactly at the same sites as in the TK6 rRNAs as described above (16,39), we suggest that the current single-particle cryo-EM technique needs to be improved for the detailed structural analysis at the ∼0.1 nm level and requires confirmation by a complementary technique such as MS.

According to the SILNAS-based quantitative analysis of the stoichiometry of PTMs at each site of human rRNAs, 172 of the 228 sites were almost fully modified (≥85%), whereas the others were modified to an extent between 5% and 85% (Table 1). Notably, we found that all of the partially modified sites reflect small nucleolar RNA–directed modifications, i.e. pseudouridylation, 2′-*O*-methylation, and base acetylation at C1337 in 18S rRNA (40). Although the biological relevance of this observation is unclear, indeed each of pseudouridylated and 2′-*O*-methylated sites of the rRNAs is sensitive to environmental stimuli that promote cellular stress, such as growth temperature in fission yeast (22). Moreover, cytidine acetylation at the corresponding position in fission yeast (C1297) also occurred partially, and yeast cells deficient in the responsible acetyltransferase encoded by *Nat10* showed a slow-growth phenotype and were defective in forming 18S rRNA from the precursor rRNA (41,42), suggesting a conserved role for cytidine acetylation in ribosome biogenesis during the evolution of eukaryotes.

### PTM clustering of rRNAs appears to have occurred during evolution

**Figure 1.**
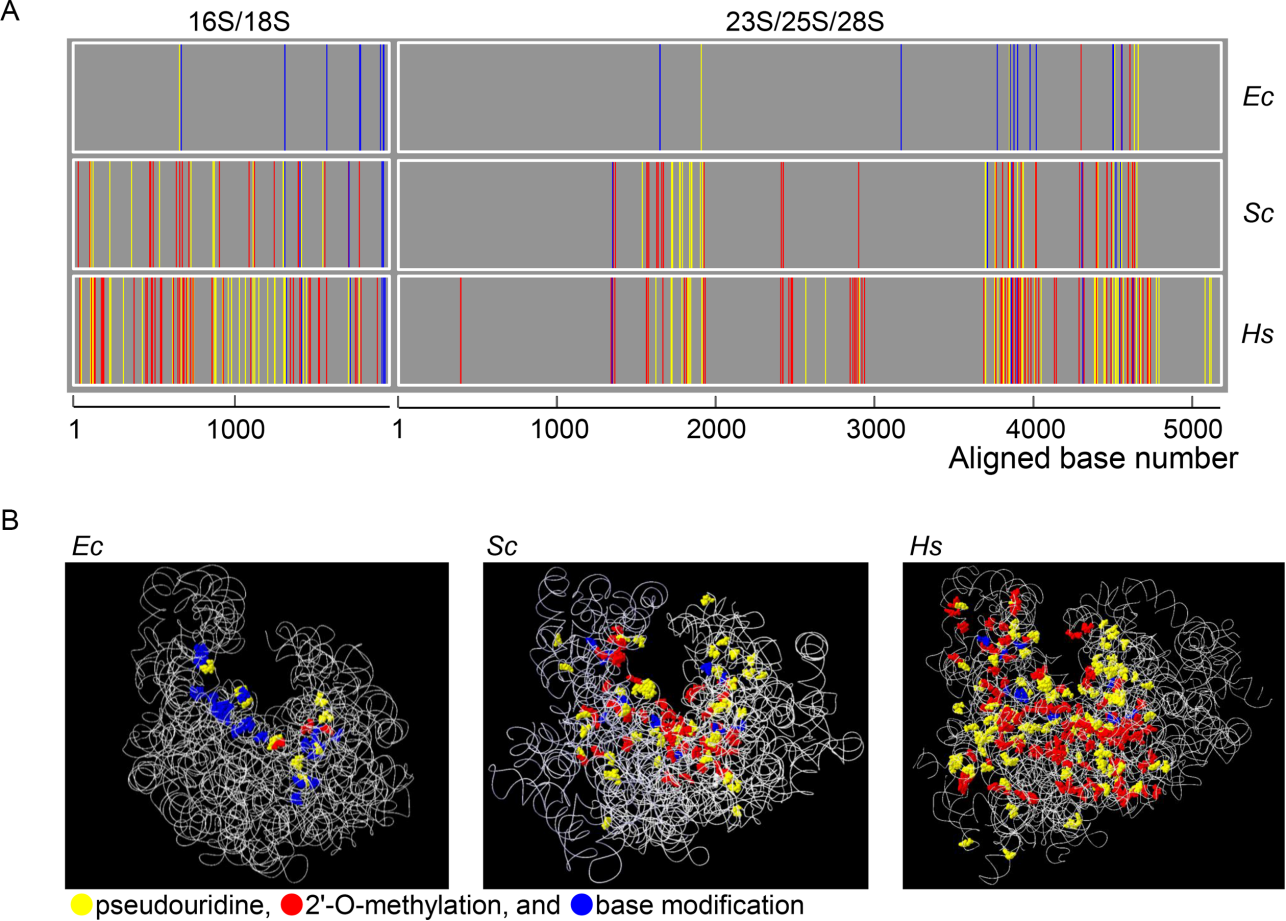
(**A**) One-dimensional PTM maps of the *E. coli* (*Ec*), budding yeast (*Saccharomyces cerevisiae, Sc*) and human (*Hs*) rRNAs. The PTM sites were plotted against rRNA sequences aligned by the sequence homology (shown in Supplementary Table S9) obtained by using the Clustal Omega software (https://www.ebi.ac.uk/Tools/msa/clustalo/). The PTM sites are colored yellow (pseudouridine), red (2′-*O*-methylated nucleoside), and blue (base-modified nucleoside). (**B**) Three-dimensional PTM maps of the *Ec, Sc*, and *Hs* ribosomes. The PTM sites were assigned to the three-dimensional structure of the rRNAs obtained from 4YBB.pdb (for *Ec*), 3U5B.pdb (*Sc*) and 4UG0.pdb (*Hs*). The RNA backbone is shown as a ribbon, and PTM sites are colored as in (A).

**Figure 2.**
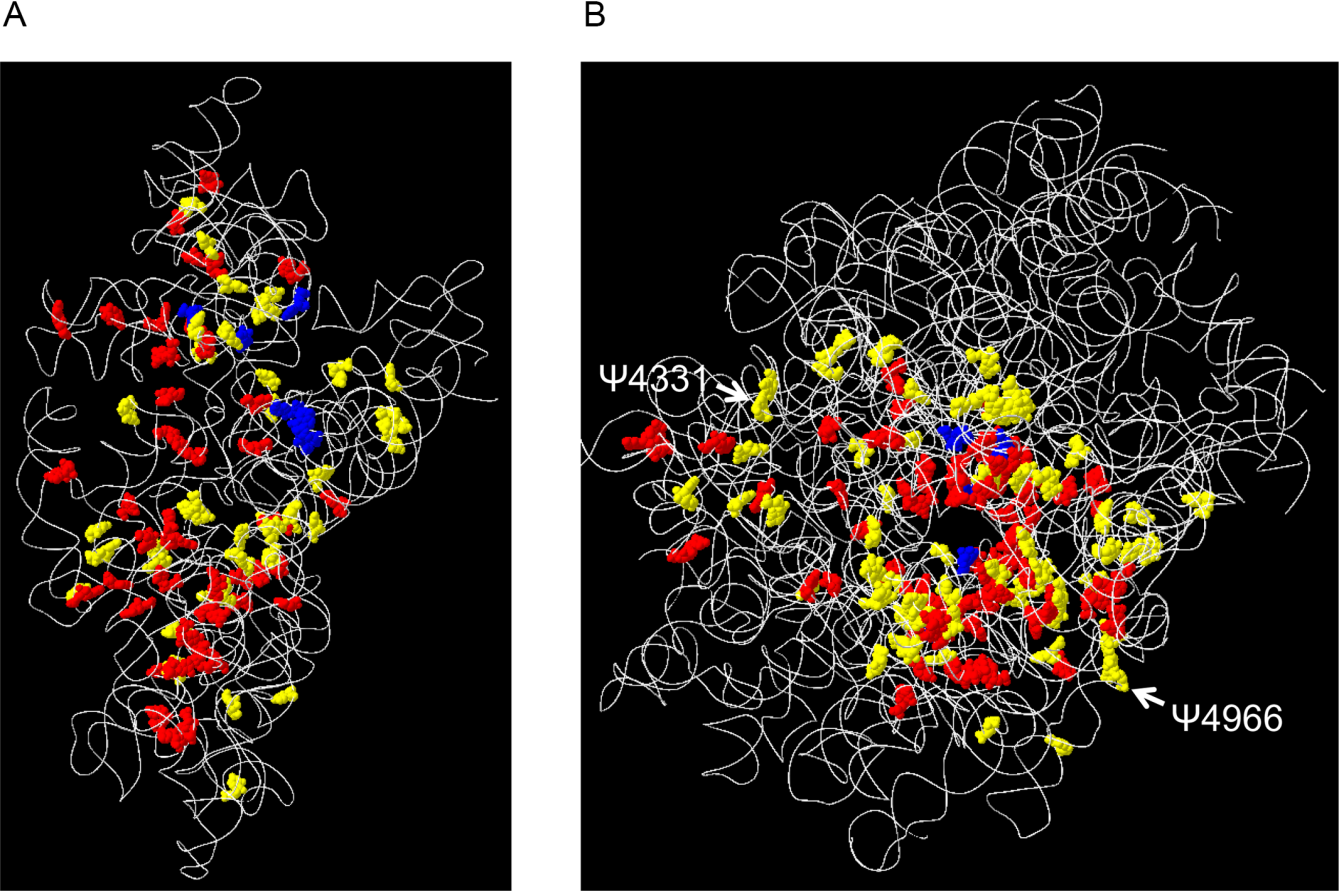
The 3D PTM map of the small (**A**) and large (**B**) subunits of the human 80S ribosome. The PTM sites were assigned to the 3D structure of human rRNAs (4UG0.pdb). The RNA backbones and PTM sites are colored as in Figure 1B. White arrows indicate the positions of Ψ4331 and Ψ4966, which are less stoichiometrically modified in patients with DC.

Although most PTMs of human rRNAs are typical of eukaryotic rRNAs and many are conserved in yeast, human rRNAs appear to accumulate much more modification sites (228 sites) than yeast rRNAs, e.g., 112 PTM for *S. cerevisiae* rRNAs (Supplementary Table S9). Furthermore, yeast rRNAs appear to have accumulated more modification sites than prokaryotic rRNAs, e.g., *Escherichia coli* has 11 modification sites in 16S rRNA and 25 in 23S rRNA (Figure 1). We found that this relatively greater number of PTMs is mostly attributable to increased pseudouridylation and 2′-*O*-methylation of the ribose moiety (Figure 1 and Supplementary Figure S7). In addition, most modification reactions occur in close proximity, forming clusters of modification sites along the rRNA chain as reported earlier by Decatur and Fournier (7); three large clusters (near positions 1600–1900, 3700–4000 and 4400–4700) and three small clusters (near positions 1300, 2400 and 2900) were detected both in the yeast 26S and human 28 rRNA. A similar type of PTM clustering also appears to have occurred in 16S/18S rRNAs during evolution (Figure 1). This appears to correlate with a rich-get-richer process or a power-law distribution (Supplementary Figure S8). For example, in biological networks, including metabolic networks (43–45), protein interactions (46), and phosphorylation (47), the power-law distribution confers functional advantages of robustness to environmental changes and tolerance to random mutations (48). Thus, we anticipate that, in human rRNAs, the clustering of PTMs might have increased the structural and functional stability of ribosomes during evolution. In this context, it should be noted that human 28S rRNA has acquired more PTMs around positions 450, 2500, 4100 and 5100 compared with the yeast counterpart, implying that these PTMs have a specific role in stabilizing the human ribosome and/or enhancing its function. On the other hand, a 3D overview of all modification sites in human rRNAs clearly indicates that most sites are concentrated in functionally important interior regions of the ribosome, including the peptidyl transferase center, the A, P and E sites for tRNA and mRNA binding, the polypeptide exit tunnel, and the interacting surfaces of the small and large subunits (Figures 1 and 2). Clearly, the PTM sites in human rRNAs expand from the interior to the exterior regions as compared with yeast rRNAs (Figure 1 and Supplementary Figure S7).

### Pseudouridylation at two specific sites of rRNA are significantly reduced in ribosomes of patients with familial DC

**Figure 3.**
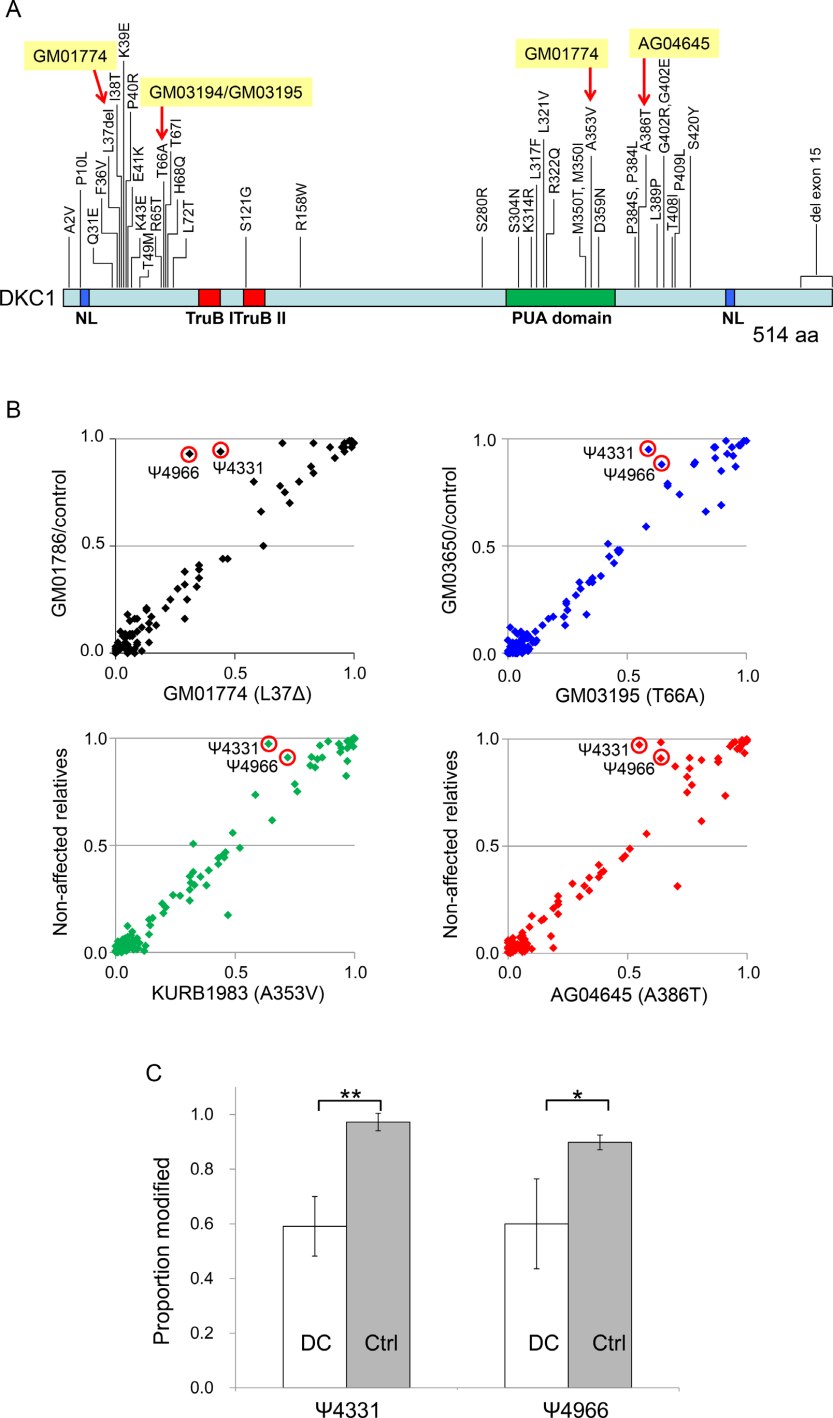
SILNAS-based PTM analysis of rRNAs from cells derived from DC patients. (**A**) The positions of amino acid (aa) substitutions or deletions in *DKC1* from DC patient cells. The nuclear localization signals (NL) and TruB and PUA domains of DKC1 are indicated. Red arrows denote mutations in patient rRNAs analyzed in this study. The figure was redrawn based on Vulliamy *et al.* (56) (http://telomerase.asu.edu/diseases.html). (**B**) Correlation between the stoichiometry of PTM in 28S rRNA from cells of DC patients and healthy controls. The PTMs in all rRNAs from the cells of five DC patients and four non-affected relatives were assessed with SILNAS (Supplementary Tables S3 and S4). The four plots compare the stoichiometry of modification in 28S rRNA derived from four DKC1 mutant cell lines (del37L, A353V, T66A, A386T) *vs*. control lines, and a significant difference was detected for Ψ4331 and Ψ4966 (red circles). Note that the average stoichiometric value of non-affected relatives was used as the control for Patients KURB1983 and AG4645 because the cells of a relative were not available for Patients KURB1983 and AG4645. (**C**) Statistical significance of differences in pseudouridylation at positions 4331 and 4966 in 28S rRNA from DC patient cells. Each value represents the mean ± S.D. of five DC patients and four relatives (Ctrl). *P < 0.005, **P < 0.0005 (one-tailed Student's *t*-test).

X-linked DC is a rare genetic disease characterized by defective tissue maintenance and cancer predisposition caused by a point mutation in the pseudouridine synthase gene *DKC1* (49); because DKC1 is also a telomere maintenance factor, however, it remains unclear which DKC1 activity is primarily responsible for the pathogenesis of DC. The autosomal dominant types of DC are a consequence of mutations in the telomerase RNA TERC (50), implying that this form of the disease is caused by a defect in telomere maintenance, whereas the mutations found in the catalytic domain of DKC1 are associated with a more severe form of DC (51), implying that this form of the disease is caused by a defect in the rRNA pseudouridine synthase activity. To examine whether mutations in *DKC1* affect the pseudouridylation of rRNAs, we performed SILNAS-based comprehensive quantification of PTMs in rRNA derived from DC patients. We cultured fibroblasts and B-lymphoblastoid cell lines derived from patients age 7–19 years old harboring four distinct mutations, namely del37L, A353V, T66A and A386T, located in the different domains of DKC1 pseudouridine synthase (Figure 3A and Supplementary Table S3); cells from relatives served as a reference. This approach identified and quantified ∼90% of the PTMs among the 5.8S, 18S and 28S rRNAs, which allowed for quantitative comparison of most rRNA PTMs from DC patients and relatives. Notably, the PTMs from nine individuals were strictly conserved with respect to not only the sites but also the stoichiometry of modification at each site (Supplementary Table S4), underscoring the paucity of PTM polymorphisms in human rRNAs. Thus, we propose that the rRNA PTMs are much more conservative than previously thought (16,39) and are tightly regulated, as noted in the PTM analysis of rRNAs derived from different cell types (Supplementary Table S5).

Based on a detailed comparison of the stoichiometry of modification at each site, however, we detected relatively less pseudouridylation at positions 4331 and 4966 in 28S rRNA derived from DC patients (Figure 3B and Supplementary Table S4). The difference in each case (∼40%) appears to be statistically significant (Figure 3C) and evident in DC patients regardless of the mutation site in *DKC1*. According to the 3D structure of the ribosome, both Ψ4331 and Ψ4966 are located at the periphery of the large subunit rather than the interior, and thereby have potential roles in stabilizing the ribosome structure *via* interactions with ribosomal proteins (Figure 2). This raises a possibility that Ψ4331 and Ψ4966 alter internal ribosomal entry site-dependent RNA translation and translational fidelity (52), and the resulting fluctuations in protein synthesis may impair the function of hematopoietic stem cells (53) and cause DC symptoms (54). Whether the reduced pseudouridylation at those sites of rRNA has a role in DC pathogenesis remains unknown, but Ψ4331 and Ψ4966 may in fact serve as novel biochemical markers for early diagnosis of DC that complements the currently available indicators, e.g., a telomere length (55). Apparently, further studies on more DC cases are required to verify the results and possibilities described above. Nevertheless, the complete survey of rRNA PTMs provided by this study will serve as a resource for exploring the functional roles of rRNA PTMs and yield the most complete atomic model of human ribosome.

## Supplementary Material

Supplementary Data

## References

[1] Khatter H., Myasnikov A.G., Natchiar S.K., Klaholz B.P. Structure of the human 80S ribosome. Nature. 2015; 520:640–645. 25901680 10.1038/nature14427

[2] Anger A.M., Armache J.P., Berninghausen O., Habeck M., Subklewe M., Wilson D.N., Beckmann R. Structures of the human and Drosophila 80S ribosome. Nature. 2013; 497:80–85. 23636399 10.1038/nature12104

[3] Quade N., Boehringer D., Leibundgut M., van den Heuvel J., Ban N. Cryo-EM structure of Hepatitis C virus IRES bound to the human ribosome at 3.9-A resolution. Nat. Commun. 2015; 6:7646. 26155016 10.1038/ncomms8646 PMC4510694

[4] Lestrade L., Weber M.J. snoRNA-LBME-db, a comprehensive database of human H/ACA and C/D box snoRNAs. Nucleic Acids Res. 2006; 34:D158–D162. 16381836 10.1093/nar/gkj002 PMC1347365

[5] Incarnato D., Anselmi F., Morandi E., Neri F., Maldotti M., Rapelli S., Parlato C., Basile G., Oliviero S. High-throughput single-base resolution mapping of RNA 2-*O*-methylated residues. Nucleic Acids Res. 2017; 45:1433–1441. 28180324 10.1093/nar/gkw810 PMC5388417

[6] Machnicka M.A., Milanowska K., Osman Oglou O., Purta E., Kurkowska M., Olchowik A., Januszewski W., Kalinowski S., Dunin-Horkawicz S., Rother K.M. MODOMICS: a database of RNA modification pathways–2013 update. Nucleic Acids Res. 2013; 41:D262–D267. 23118484 10.1093/nar/gks1007 PMC3531130

[7] Decatur W.A., Fournier M.J. rRNA modifications and ribosome function. Trends Biochem. Sci. 2002; 27:344–351. 12114023 10.1016/s0968-0004(02)02109-6

[8] Noeske J., Wasserman M.R., Terry D.S., Altman R.B., Blanchard S.C., Cate J.H. High-resolution structure of the Escherichia coli ribosome. Nat. Struct. Mol. Biol. 2015; 22:336–341. 25775265 10.1038/nsmb.2994 PMC4429131

[9] Polikanov Y.S., Melnikov S.V., Soll D., Steitz T.A. Structural insights into the role of rRNA modifications in protein synthesis and ribosome assembly. Nat. Struct. Mol. Biol. 2015; 22:342–344. 25775268 10.1038/nsmb.2992 PMC4401423

[10] Sharma S., Lafontaine D.L. ‘View from a Bridge’: A new perspective on eukaryotic rRNA base modification. Trends Biochem. Sci. 2015; 40:560–575. 26410597 10.1016/j.tibs.2015.07.008

[11] Burakovsky D.E., Prokhorova I.V., Sergiev P.V., Milon P., Sergeeva O.V., Bogdanov A.A., Rodnina M.V., Dontsova O.A. Impact of methylations of m2G966/m5C967 in 16S rRNA on bacterial fitness and translation initiation. Nucleic Acids Res. 2012; 40:7885–7895. 22649054 10.1093/nar/gks508 PMC3439901

[12] Liang X.H., Liu Q., Fournier M.J. rRNA modifications in an intersubunit bridge of the ribosome strongly affect both ribosome biogenesis and activity. Mol. Cell. 2007; 28:965–977. 18158895 10.1016/j.molcel.2007.10.012

[13] Baudin-Baillieu A., Fabret C., Liang X.H., Piekna-Przybylska D., Fournier M.J., Rousset J.P. Nucleotide modifications in three functionally important regions of the Saccharomyces cerevisiae ribosome affect translation accuracy. Nucleic Acids Res. 2009; 37:7665–7677. 19820108 10.1093/nar/gkp816 PMC2794176

[14] Kimura S., Suzuki T. Fine-tuning of the ribosomal decoding center by conserved methyl-modifications in the Escherichia coli 16S rRNA. Nucleic Acids Res. 2010; 38:1341–1352. 19965768 10.1093/nar/gkp1073 PMC2831307

[15] Motorin Y., Helm M. RNA nucleotide methylation. Wiley Interdiscip. Rev. RNA. 2011; 2:611–631. 21823225 10.1002/wrna.79

[16] McMahon M., Contreras A., Ruggero D. Small RNAs with big implications: new insights into H/ACA snoRNA function and their role in human disease. Wiley Interdiscip. Rev. RNA. 2015; 6:173–189. 25363811 10.1002/wrna.1266 PMC4390053

[17] Sloan K.E., Warda A.S., Sharma S., Entian K.D., Lafontaine D.L.J., Bohnsack M.T. Tuning the ribosome: The influence of rRNA modification on eukaryotic ribosome biogenesis and function. RNA Biol. 2017; 14:1138–1152. 27911188 10.1080/15476286.2016.1259781 PMC5699541

[18] Sergiev P.V., Aleksashin N.A., Chugunova A.A., Polikanov Y.S., Dontsova O.A. Structural and evolutionary insights into ribosomal RNA methylation. Nat. Chem. Biol. 2018; 14:226–235. 29443970 10.1038/nchembio.2569

[19] Natchiar S.K., Myasnikov A.G., Kratzat H., Hazemann I., Klaholz B.P. Visualization of chemical modifications in the human 80S ribosome structure. Nature. 2017; 551:472–477. 29143818 10.1038/nature24482

[20] Krogh N., Jansson M.D., Hafner S.J., Tehler D., Birkedal U., Christensen-Dalsgaard M., Lund A.H., Nielsen H. Profiling of 2′-O-Me in human rRNA reveals a subset of fractionally modified positions and provides evidence for ribosome heterogeneity. Nucleic Acids Res. 2016; 44:7884–7895. 27257078 10.1093/nar/gkw482 PMC5027482

[21] Carlile T.M., Rojas-Duran M.F., Zinshteyn B., Shin H., Bartoli K.M., Gilbert W.V. Pseudouridine profiling reveals regulated mRNA pseudouridylation in yeast and human cells. Nature. 2014; 515:143–146. 25192136 10.1038/nature13802 PMC4224642

[22] Taoka M., Nobe Y., Hori M., Takeuchi A., Masaki S., Yamauchi Y., Nakayama H., Takahashi N., Isobe T. A mass spectrometry-based method for comprehensive quantitative determination of post-transcriptional RNA modifications: the complete chemical structure of Schizosaccharomyces pombe ribosomal RNAs. Nucleic Acids Res. 2015; 43:e115. 26013808 10.1093/nar/gkv560 PMC4605285

[23] Taoka M., Nobe Y., Yamaki Y., Yamauchi Y., Ishikawa H., Takahashi N., Nakayama H., Isobe T. The complete chemical structure of Saccharomyces cerevisiae rRNA: partial pseudouridylation of U2345 in 25S rRNA by snoRNA snR9. Nucleic Acids Res. 2016; 44:8951–8961. 27325748 10.1093/nar/gkw564 PMC5062969

[24] Shalev-Benami M., Zhang Y., Rozenberg H., Nobe Y., Taoka M., Matzov D., Zimmerman E., Bashan A., Isobe T., Jaffe C.L. Atomic resolution snapshot of Leishmania ribosome inhibition by the aminoglycoside paromomycin. Nat. Commun. 2017; 8:1589. 29150609 10.1038/s41467-017-01664-4 PMC5693986

[25] Takashima Y., Sakuraba M., Koizumi T., Sakamoto H., Hayashi M., Honma M. Dependence of DNA double strand break repair pathways on cell cycle phase in human lymphoblastoid cells. Environ. Mol. Mutagen. 2009; 50:815–822. 19402155 10.1002/em.20481

[26] Ishikawa H., Yoshikawa H., Izumikawa K., Miura Y., Taoka M., Nobe Y., Yamauchi Y., Nakayama H., Simpson R.J., Isobe T. Poly(A)-specific ribonuclease regulates the processing of small-subunit rRNAs in human cells. Nucleic Acids Res. 2017; 45:3437–3447. 27899605 10.1093/nar/gkw1047 PMC5389690

[27] Keka I.S., Mohiuddin, Maede Y., Rahman M.M., Sakuma T., Honma M., Yamamoto T., Takeda S., Sasanuma H. Smarcal1 promotes double-strand-break repair by nonhomologous end-joining. Nucleic Acids Res. 2015; 43:6359–6372. 26089390 10.1093/nar/gkv621 PMC4513880

[28] Yamauchi Y., Taoka M., Nobe Y., Izumikawa K., Takahashi N., Nakayama H., Isobe T. Denaturing reversed phase liquid chromatographic separation of non-coding ribonucleic acids on macro-porous polystyrene-divinylbenzene resins. J. Chromatogr. A. 2013; 1312:87–92. 24044980 10.1016/j.chroma.2013.09.021

[29] Taoka M., Yamauchi Y., Nobe Y., Masaki S., Nakayama H., Ishikawa H., Takahashi N., Isobe T. An analytical platform for mass spectrometry-based identification and chemical analysis of RNA in ribonucleoprotein complexes. Nucleic Acids Res. 2009; 37:e140. 19740761 10.1093/nar/gkp732 PMC2790879

[30] Nakayama H., Yamauchi Y., Taoka M., Isobe T. Direct identification of human cellular microRNAs by nanoflow liquid chromatography-high-resolution tandem mass spectrometry and database searching. Anal. Chem. 2015; 87:2884–2891. 25662820 10.1021/ac504378s

[31] Yamauchi Y., Nobe Y., Izumikawa K., Higo D., Yamagishi Y., Takahashi N., Nakayama H., Isobe T., Taoka M. A mass spectrometry-based method for direct determination of pseudouridine in RNA. Nucleic Acids Res. 2015; 44:e59. 26673725 10.1093/nar/gkv1462 PMC4824092

[32] Nakayama H., Akiyama M., Taoka M., Yamauchi Y., Nobe Y., Ishikawa H., Takahashi N., Isobe T. Ariadne: a database search engine for identification and chemical analysis of RNA using tandem mass spectrometry data. Nucleic Acids Res. 2009; 37:e47. 19270066 10.1093/nar/gkp099 PMC2665244

[33] Popova A.M., Williamson J.R. Quantitative analysis of rRNA modifications using stable isotope labeling and mass spectrometry. J. Am. Chem. Soc. 2014; 136:2058–2069. 24422502 10.1021/ja412084b PMC3985470

[34] Ofengand J., Bakin A. Mapping to nucleotide resolution of pseudouridine residues in large subunit ribosomal RNAs from representative eukaryotes, prokaryotes, archaebacteria, mitochondria and chloroplasts. J. Mol. Biol. 1997; 266:246–268. 9047361 10.1006/jmbi.1996.0737

[35] Maden B.E., Salim M. The methylated nucleotide sequences in HELA cell ribosomal RNA and its precursors. J. Mol. Biol. 1974; 88:133–152. 4374549 10.1016/0022-2836(74)90299-x

[36] Lowe T.M., Eddy S.R. A computational screen for methylation guide snoRNAs in yeast. Science. 1999; 283:1168–1171. 10024243 10.1126/science.283.5405.1168

[37] Jorjani H., Kehr S., Jedlinski D.J., Gumienny R., Hertel J., Stadler P.F., Zavolan M., Gruber A.R. An updated human snoRNAome. Nucleic Acids Res. 2016; 44:5068–5082. 27174936 10.1093/nar/gkw386 PMC4914119

[38] Parks M.M., Kurylo C.M., Dass R.A., Bojmar L., Lyden D., Vincent C.T., Blanchard S.C. Variant ribosomal RNA alleles are conserved and exhibit tissue-specific expression. Sci. Adv. 2018; 4:eaao0665. 29503865 10.1126/sciadv.aao0665 PMC5829973

[39] Xue S., Barna M. Specialized ribosomes: a new frontier in gene regulation and organismal biology. Nat. Rev. Mol. Cell Biol. 2012; 13:355–369. 22617470 10.1038/nrm3359 PMC4039366

[40] Sharma S., Yang J., van Nues R., Watzinger P., Kotter P., Lafontaine D.L.J., Granneman S., Entian K.D. Specialized box C/D snoRNPs act as antisense guides to target RNA base acetylation. PLos Genet. 2017; 13:e1006804. 28542199 10.1371/journal.pgen.1006804 PMC5464676

[41] Li Z., Lee I., Moradi E., Hung N.J., Johnson A.W., Marcotte E.M. Rational extension of the ribosome biogenesis pathway using network-guided genetics. PLoS Biol. 2009; 7:e1000213. 19806183 10.1371/journal.pbio.1000213 PMC2749941

[42] Taoka M., Ishikawa D., Nobe Y., Ishikawa H., Yamauchi Y., Terukina G., Nakayama H., Hirota K., Takahashi N., Isobe T. RNA cytidine acetyltransferase of small-subunit ribosomal RNA: identification of acetylation sites and the responsible acetyltransferase in fission yeast, Schizosaccharomyces pombe. PLoS One. 2014; 9:e112156. 25402480 10.1371/journal.pone.0112156 PMC4234376

[43] Fell D.A., Wagner A. The small world of metabolism. Nat. Biotechnol. 2000; 18:1121–1122. 11062388 10.1038/81025

[44] Jeong H., Tombor B., Albert R., Oltvai Z.N., Barabasi A.L. The large-scale organization of metabolic networks. Nature. 2000; 407:651–654. 11034217 10.1038/35036627

[45] Ravasz E., Somera A.L., Mongru D.A., Oltvai Z.N., Barabasi A.L. Hierarchical organization of modularity in metabolic networks. Science. 2002; 297:1551–1555. 12202830 10.1126/science.1073374

[46] Jeong H., Mason S.P., Barabasi A.L., Oltvai Z.N. Lethality and centrality in protein networks. Nature. 2001; 411:41–42. 11333967 10.1038/35075138

[47] Yachie N., Saito R., Sugahara J., Tomita M., Ishihama Y. In silico analysis of phosphoproteome data suggests a rich-get-richer process of phosphosite accumulation over evolution. Mol. Cell. Proteomics. 2009; 8:1061–1071. 19136663 10.1074/mcp.M800466-MCP200 PMC2689765

[48] Przulj N. Protein-protein interactions: making sense of networks via graph-theoretic modeling. Bioessays. 2011; 33:115–123. 21188720 10.1002/bies.201000044

[49] Calado R.T., Young N.S. Telomere maintenance and human bone marrow failure. Blood. 2008; 111:4446–4455. 18239083 10.1182/blood-2007-08-019729 PMC2343587

[50] Vulliamy T., Marrone A., Goldman F., Dearlove A., Bessler M., Mason P.J., Dokal I. The RNA component of telomerase is mutated in autosomal dominant dyskeratosis congenita. Nature. 2001; 413:432–435. 11574891 10.1038/35096585

[51] Dokal I. Dyskeratosis congenita in all its forms. Br. J. Haematol. 2000; 110:768–779. 11054058 10.1046/j.1365-2141.2000.02109.x

[52] Penzo M., Rocchi L., Brugiere S., Carnicelli D., Onofrillo C., Coute Y., Brigotti M., Montanaro L. Human ribosomes from cells with reduced dyskerin levels are intrinsically altered in translation. FASEB J. 2015; 29:3472–3482. 25934701 10.1096/fj.15-270991

[53] Signer R.A., Magee J.A., Salic A., Morrison S.J. Haematopoietic stem cells require a highly regulated protein synthesis rate. Nature. 2014; 509:49–54. 24670665 10.1038/nature13035 PMC4015626

[54] Yu Y.T., Meier U.T. RNA-guided isomerization of uridine to pseudouridine–pseudouridylation. RNA Biol. 2015; 11:1483–1494. 10.4161/15476286.2014.972855 PMC4615163 25590339

[55] Townsley D.M., Dumitriu B., Young N.S. Bone marrow failure and the telomeropathies. Blood. 2014; 124:2775–2783. 25237198 10.1182/blood-2014-05-526285 PMC4215309

[56] Vulliamy T.J., Marrone A., Knight S.W., Walne A., Mason P.J., Dokal I. Mutations in dyskeratosis congenita: their impact on telomere length and the diversity of clinical presentation. Blood. 2006; 107:2680–2685. 16332973 10.1182/blood-2005-07-2622

