# Supplementary material for: Landscape of the complete RNA chemical modifications in the human 80S ribosome
Source: Nucleic Acids Res. 2018 Sep 7;46(18):9289–98. doi: 10.1093/nar/gky811 (PMC6182160; doi:10.1093/nar/gky811)

Supplementary information for

Landscape of the complete RNA chemical modifications in the human 80S ribosome

Masato Taoka*[1], Yuko Nobe[1], Yuka Yamaki[1], Ko Sato[1], Hideaki Ishikawa[2], Keiichi Izumikawa[2], Yoshio Yamauchi[1], Kouji Hirota[1], Hiroshi Nakayama[3], Nobuhiro Takahashi[2], Toshiaki Isobe*[1]

[1]Department of Chemistry, Graduate School of Science, Tokyo Metropolitan University, Minami-osawa 1-1, Hachioji-shi, Tokyo 192-0397, Japan
[2]Department of Biotechnology, Global innovation research institute, Tokyo University of Agriculture and Technology, Saiwai-cho 3-5-8, Fuchu-shi, Tokyo 183-8509, Japan
[3]Biomolecular Characterization Unit, RIKEN Center for Sustainable Resource Science, 2-1 Hirosawa, Wako, Saitama 351-0198, Japan

*Corresponding authors:
Masato Taoka, Ph.D., Laboratory of Biochemistry, Department of Chemistry, Graduate School of Science, Tokyo Metropolitan University, Minami-osawa 1-1, Hachioji-shi, Tokyo 192-0397, Japan
Tel: +81 426 77 2543; Fax: +81 426 77 2525
Email: mango@tmu.ac.jp

Toshiaki Isobe, Ph.D., Laboratory of Biochemistry, Department of Chemistry, Graduate School of Science, Tokyo Metropolitan University, Minami-osawa 1-1, Hachioji-shi, Tokyo 192-0397, Japan
Tel: +81 426 77 5667; Fax: +81 426 77 2525
Email: isobe-toshiaki@tmu.ac.jp

SUPPLEMENTARY FIGURELEGEND

Supplementary Figure S1. Purification of *in vitro* transcribed human reference rRNAs used for SILNAS.

(A-D) RP-LC purification of the reference rRNAs. Each rRNA transcribed *in vitro* was separated by RP-LC as described in Materials and Methods. The fraction indicated in the figure (sectioned with blue line) was examined by electrophoresis shown in panel D and used for SILNAS as a quantitation standard. (E) Electropherogram of the purified reference RNAs. The rRNAs were analyzed on an Agilent Bioanalyzer 2100 (Agilent technologies) with Agilent RNA 6000 Pico molecular marker kits (1 ng each RNA). 1, RNA marker; 2, 5S rRNA; 3, 5.8S rRNA; 4,18S rRNA; 5, 28S rRNA. Although the synthetic 28S rRNA showed slightly faster mobility than that expected from the molecular size (5,106 bases), we confirmed that the authentic human 28S rRNA exhibited the same behavior on the Bioanalyzer.

Supplementary Figure S2. The complete sequence and PTMs of the human small ribosomal subunit rRNA (18S) and the fragments used for the structural analysis.

Black solid and black shaded bars denote the fragments produced by RNase T1 and RNase A digestion, respectively, of 18S rRNA. Blue arrows denote RNase H–digested fragments of 18S rRNA. Blue solid and blue shaded bars denote RNase T1–digested and RNase A–digested fragments of the RNase H fragments, respectively. All fragments were identified by Ariadne software (see Methods) except for several heavily modified RNA fragments, which were identified by manual inspection of their MS/MS spectra. The numbering of nucleotides is performed according to the snoRNA-LBME database (Nucleic Acids Res. 2006;34(Database issue):D158-62. http://www-snorna.biotoul.fr/). Modified nucleotides are shown as blue letters, and their abbreviations are summarized as follows:

P, pseudouridine; Λ, 2'-O-methyladenosine; B, 2'-O-methylcytidine; #, 2'-O-methylguanosine; J, 2'-O-methyluridine; Z, 2'-O-methylpseudouridine; Π, 1-methyladenosine; ζ, N6, N6-dimethyladenosine; Σ, 5-methylcytidine;
7, 7-methylguanosine; δ, 3-methyluridine; π, N6-methyladenosine; M, N4-acetylcytidine; α, 1-methyl-3-(3-amino-3-carboxypropyl)pseudouridine.

Supplementary Figure S3. The complete sequence and PTMs of the human large ribosomal subunit rRNAs (28S, 5S, and 5.8S) and the fragments used for the structural analysis.

The details and abbreviations are as described in Supplementary Figure S2.

Supplementary Figure S4. Human rRNAs contain unmodified U at position 688 in 18S rRNA and unmodified C and U at position 2279 and 4501, respectively, in 28S rRNA.

S4-1. U688 in 18S rRNA.

Cellular 18S rRNA and *in vitro* transcribed reference ($^{13}C_{10}$-guanosine-labeled) 18S rRNA were purified by reversed-phase HPLC. The resulting "light" and "heavy" fragments were mixed at a 1: 1 ratio, digested with RNase T1, and subjected to LC-MS analysis. In principle, RNase T1 should produce three types of fragments, namely AUCUUGp$_{685\text{-}690}$, AUCUU*Gp$_{685\text{-}690}$ (*G, $^{13}C_{10}$-guanosine) and modified AUCUUGp$_{685\text{-}690}$ (AΨCUUGp$_{685\text{-}690}$, AUCΨUGp$_{685\text{-}690}$, AΨCΨUGp$_{685\text{-}690}$, etc.), from the sequence 685-690 of each of the cellular and reference 18S rRNAs. **(A)** Extracted ion monitoring of AUCUUGp and its cognate fragment. The AUCUUGp-cognate fragments were obtained by RNase T1 digestion of the purified 18S rRNAs. Oligonucleotide ion masses are shown in the figure. The mass windows used for extraction were ±5 ppm. In the LC-MS analysis, the heavy fragments AUCUU*Gp$_{685\text{-}690}$ (upper panel) and the light fragments AUCUUGp$_{685\text{-}690}$ (lower panel) eluted at the same time. A modified AUCUUGp$_{685\text{-}690}$ was also detected (marked with "‡"). In this fragment, at least one uridine was pseudouridylated (according to the signature signal m/z 207.041 in MS/MS, data not shown). **(B)** Identification of AUCUUGp$_{685\text{-}690}$ cognate and its site of pseudouridylation analyzed by database searching *via* the human rRNA sequences with Ariadne. The modified AUCUUGp$_{685\text{-}690}$ (in the peak corresponding to the one marked with "‡" in panel A) was separated from the RNase T1 digest of C/U-5,6-d2-labeled 18S rRNA. The MS/MS spectrum (parent *m/z* 960.130) was obtained by collision-induced dissociation. Full sequence coverage of AΨCUUGp$_{685\text{-}690}$ was achieved using the a, b, c, d, w, x, y and z ions and their derivatives. Identified signal, red line with black letter; the signals possessing the theoretical mass with permissible error and undetermined ionic valence, blue line with blue letter; the signals of internally cleaved fragments or molecular mass without a base, black line with gray letter. Errors of identified signals are placed under the MS/MS. Note that no Ψ688-containing fragment was detected. **(C)** The dissociated ions identified from the MS/MS signals for AΨCUUGp$_{685\text{-}690}$. Red/bold: the identified signals; blue/bold: signals representing the theoretical mass with permissible error and undetermined ionic valence.

S4-2. C2279 in 28S rRNA.

Extracted ion monitoring of the 28S rRNA fragments containing C2279 is shown. The RNase H fragments H15 from cellular 28S rRNA and *in vitro* transcribed reference ($^{13}C_{10}$-guanosine-labeled) 28S rRNA were purified by reversed-phase HPLC. The resulting "light" and "heavy" fragments were mixed with a 1: 1 ratio, digested with RNase T1, and

subjected to LC-MS analysis. In principle, RNase T1 should produce three types of fragments, namely CUGp, CU*Gp, and modified $CUGp_{2279-2281}$ ($CmUGp_{2279-2281}$), from the sequence 2040-2042 and 2279-2281 of each of the RNase H fragment of the cellular and reference 28S rRNA. The CUGp-cognate fragments were obtained by RNase T1 digestion of the purified RNase H fragment H15 of 28S rRNAs. In the LC-MS analysis, the heavy fragments CU*Gp (upper panel) and the light fragments CUGp (middle panel), eluted at the same time. The most intense signal in the top panel was set to 100%, and the peak in the middle and bottom panel scaled accordingly. From the signal heights of the MS spectra of the light and heavy CUGp ions, the stoichiometry of modification was estimated as 0%. Note that the modified $CUGp_{2279-2281}$ ($CmUGp_{2279-2281}$, m/z 987.133, bottom panel) was not detected. Note that the methylation of C2279 is also denied by Krogh et al. (Nucleic Acids Res. 2016;44(16):7884-95).

S4-3. U4501 in 28S rRNA.

Cellular 28S rRNA and *in vitro* transcribed reference ($^{13}C_{10}$-guanosine-labeled) 28S rRNA were purified by reversed-phase HPLC. The resulting “light” and “heavy” fragments were mixed at a 1: 1 ratio, digested with RNase T1, and subjected to LC-MS analysis. In principle, RNase T1 should produce three types of fragment, namely UUUAGp, UUUA*Gp and modified $UUUAGp_{4500-4504}$ ($m^3UUUAGp_{4500-4504}$, $m^3U\Psi UAGp_{4500-4504}$, $m^3UU\Psi AGp_{4500-4504}$, $m^3U\Psi\Psi AGp_{4500-4504}$, etc.), from the sequence 4500-4504 of each of the cellular and reference 28S rRNAs. **(A)** Extracted ion monitoring of UUUAGp and its cognate fragment. The UUUAGp-cognate fragments were obtained by RNase T1 digestion of the purified 28S rRNAs. In the LC-MS analysis, only the heavy fragment $UUUA{*}Gp_{4500-4504}$ eluted (upper panel), and the fragment corresponding $UUUAGp_{4500-4504}$ was not detected (middle panel), demonstrating that the cellular fragment was 100% modified. A modified $UUUAGp_{4500-4504}$ (m/z 811.094) fragment was also detected. This fragment contained the monomethylated uridine at position 4500, and at least one pseudouridylated uridine (according to the signature signal m/z 207.041 in MS/MS, data not shown). **(B)** Identification of $m^3UUUAGp_{4500-4504}$ cognate and the site of pseudouridylation analyzed by database searching *via* the human rRNA sequences with Ariadne. The modified $UUUAGp_{4500-4504}$ (in the peak corresponding to the one marked with "‡" in panel A) was separated from the RNase T1 digest of C/U-5,6-d2-labeled 28S rRNA. The MS/MS spectrum (parent *m/z* 813.611) was obtained by collision-induced dissociation. Full sequence coverage of $m^3UU\Psi AGp_{4500-4504}$ was achieved using the a, b, c, d, w, x, y and z ions and their derivatives. Note that no Ψ4501-containing fragment was detected. **(C)** The dissociated ions identified from the MS/MS signals for $m^3UU\Psi AGp_{4500-4504}$.

Supplementary Figure S5. Human rRNAs contain 10 previously unknown PTM sites.

S5-1. Cm621 in 18S rRNA.

The RNase H fragments H3 from cellular 18S rRNA and *in vitro* transcribed reference ($^{13}C_{10}$-guanosine-labeled) 18S rRNA were purified by reversed-phase -HPLC. The resulting "light" and "heavy" fragments were mixed with a 1: 1 ratio, digested with RNase T1, and subjected to LC-MS analysis. In principle, RNase T1 should produce five fragments, namely CCGp$_{621\text{-}623}$, CCGp$_{710\text{-}712}$, CC*Gp$_{621\text{-}623}$ (*G, $^{13}C_{10}$-guanosine), CC*Gp$_{710\text{-}712}$ and modified CCGp$_{621\text{-}623}$ (CmCGp$_{621\text{-}623}$), from the sequence 621-623 and 710-712 of each of the cellular and reference 18S rRNA. In the prior analysis using the RNase H fragment H3 digested with RNase A, we confirmed that C710 in 18S rRNA did not contain 2'-O-methyl residue (data not shown). **(A)** Extracted ion monitoring of CCGp and its cognate fragments. The CCGp-cognate fragments were obtained by RNase T1 digestion of the purified RNase H fragments H3 of 18S rRNA. In the LC-MS analysis, the fragments CCGp$_{621\text{-}623}$ and CCGp$_{710\text{-}712}$ and the corresponding heavy fragments CC*Gp$_{621\text{-}623}$ and CC*Gp$_{710\text{-}712}$, eluted at the same time. Oligonucleotide ion masses are shown in the right. The mass windows used for extraction were ± 5 ppm. From the signal heights of MS spectra of the light and heavy CCGp ions, the stoichiometry of modification was estimated as 62%. **(B)** Identification of CmCGp$_{621\text{-}623}$ and the site of 2'-*O*-methylation analyzed by database searching *via* the human rRNA sequences with Ariadne. CmCGp$_{621\text{-}623}$ was separated from the RNase T1 digests of cellular 18S rRNA. The MS/MS spectrum was obtained from [CmCGp]$^{-}$, m/z 986.151 by collision-induced dissociation. Full sequence coverage of CmCGp$_{621\text{-}623}$ was achieved using the a, b, c, d, w, x, y and z ions and their derivatives. Identified signal, red line with black letter; the signals possessing the theoretical mass with permissible error and undetermined ionic valence, blue line with blue letter; the signals of internally cleaved fragments or molecular mass without a base, black line with gray letter. Errors of identified signals are placed under the MS/MS. **(C)** The dissociated ions identified from the MS/MS signals for CmCGp$_{621\text{-}623}$. Red/bold: the identified signals; blue/bold: signals representing the theoretical mass with permissible error and undetermined ionic valence.

S5-2. Ψ897 in 18S rRNA.

Cellular 18S rRNA and *in vitro* transcribed reference ($^{13}C_{10}$-guanosine-labeled) 18S rRNA were purified by reversed-phase HPLC. The resulting "light" and "heavy" rRNAs were mixed at a 1: 1 ratio, digested with RNase T1, and subjected to LC-MS analysis. In principle, RNase T1 should produce three fragments, namely UUUUCGp$_{896\text{-}901}$, UUUUC*Gp$_{896\text{-}901}$, and modified UUUUCGp$_{896\text{-}901}$ (UΨUUCGp$_{896\text{-}901}$), from the sequence 896-901 of each of

the cellular and reference 18S rRNAs. **(A)** Extracted ion monitoring of UUUUCGp and its cognate fragment. The UUUUCGp-cognate fragments were obtained by RNase T1 digestion of the purified 18S rRNA. In the LC-MS analysis, the fragment $UUUUCGp_{896\text{-}901}$ and the corresponding heavy fragment $UUUUC^{*}Gp_{896\text{-}901}$ eluted at the same time. Oligonucleotide ion masses are shown in the right. The mass windows used for extraction were ±5 ppm. From the signal heights of the MS spectra of the light and heavy UUUUCGp ions, the stoichiometry of modification was estimated as 23%. **(B)** Identification of $U\Psi UUCGp_{896\text{-}901}$ and the site of pseudouridylation analyzed by database searching *via* the human rRNA sequences with Ariadne. $U\Psi UUCGp_{896\text{-}901}$ was separated from the RNase T1 digest of C/U-5,6-d2-labeled 18S rRNA. The MS/MS spectrum was obtained for $[U\Psi UUCGp]^{2-}$ (m/z 949.623) by collision-induced dissociation. Full sequence coverage of $U\Psi UUCGp_{896\text{-}901}$ was achieved using the a, b, c, d, w, x, y and z ions and their derivatives. **(C)** The dissociated ions identified from the MS/MS signals for $U\Psi UUCGp_{896\text{-}901}$.

S5-3. Ψ1045 in 18S rRNA.

Cellular 18S rRNA and *in vitro* transcribed reference ($^{13}C_{10}$-guanosine-labeled) 18S rRNA were purified by reversed-phase HPLC. The resulting “light” and “heavy” rRNAs were mixed at a 1: 1 ratio, digested with RNase T1, and subjected to LC-MS analysis. In principle, RNase T1 should produce five fragments, namely $UUCGp_{422\text{-}425}$, $UUCGp_{1045\text{-}1048}$, $UUC^{*}Gp_{422\text{-}425}$, $UUC^{*}Gp_{1045\text{-}1048}$ and modified $UUCGp_{1045\text{-}1048}$ ($\Psi\Psi CGp_{1045\text{-}1048}$), from the sequence 422-425 and 1045-1048 of each of the cellular and reference 18S rRNAs. In the prior analysis using RNase H fragment H2 digested with RNase T1, we confirmed that U422 in 18S rRNA did not contain Ψ (data not shown). **(A)** Extracted ion monitoring of UUCGp and its cognate fragment. The UUCGp-cognate fragments were obtained by RNase T1 digestion of the purified 18S rRNA. In the LC-MS analysis, the fragment $UUCGp_{422\text{-}425}$ and $UUCGp_{1045\text{-}1048}$ and the corresponding heavy fragment $UUC^{*}Gp_{422\text{-}425}$ and $UUC^{*}Gp_{1045\text{-}1048}$ eluted at the same time. Oligonucleotide ion masses are shown in the right. The mass windows used for extraction were ±5 ppm. From the signal heights of the MS spectra of the light and heavy UUCGp ions, the stoichiometry of modification was estimated as 92%. **(B)** Identification of $\Psi\Psi CGp_{1045\text{-}1048}$ and the site of pseudouridylation analyzed by database searching *via* the human rRNA sequences with Ariadne. $\Psi\Psi CGp_{1045\text{-}1048}$ was separated from the RNase T1 digest of C/U-5,6-d2-labeled 18S rRNA. The MS/MS spectrum was obtained for $[\Psi\Psi CGp]^{2-}$ (m/z 641.081) by collision-induced dissociation. Full sequence coverage of $\Psi\Psi CGp_{1045\text{-}1048}$ was achieved using the a, b, c, d, w, x, y and z ions and their derivatives. **(C)** The dissociated ions identified from the MS/MS signals for $\Psi\Psi CGp_{1045\text{-}1048}$.

S5-4. Ψ1136 in 18S rRNA.

Cellular 18S rRNA and *in vitro* transcribed reference ($^{13}C_{10}$-guanosine-labeled) 18S rRNA were purified by reversed-phase HPLC. The resulting “light” and “heavy” rRNAs were mixed at a 1: 1 ratio, digested with RNase T1, and subjected to LC-MS analysis. In principle, RNase T1 should produce CUUCCGp$_{1135\text{-}1140}$, CUUCC*Gp$_{1135\text{-}1140}$ and modified CUUCCGp$_{1135\text{-}1140}$ (CΨUCCGp$_{1135\text{-}1140}$), from the sequence 1135-1140 of each of the cellular and reference 18S rRNAs. **(A)** Extracted ion monitoring of CUUCCGp and its cognate fragment. The CUUCCGp-cognate fragments were obtained by RNase T1 digestion of the purified 18S rRNA. In the LC-MS analysis, the fragment CUUCCGp$_{1135\text{-}1140}$ and the corresponding heavy fragment CUUCC*Gp$_{1135\text{-}1140}$ eluted at the same time. Oligonucleotide ion masses are shown in the right. The mass windows used for extraction were ±5 ppm. From the signal heights of the MS spectra of the light and heavy CUUCCGp ions, the stoichiometry of modification was estimated as 7%. **(B)** Identification of CΨUCCGp$_{1135\text{-}1140}$ and the site of pseudouridylation analyzed by database searching *via* the human rRNA sequences with Ariadne. CΨUCCGp$_{1135\text{-}1140}$ was separated from the RNase T1 digest of C/U-5,6-d2-labeled 18S rRNA. The MS/MS spectrum was obtained for [CΨUCCGp]$^{2-}$ (m/z 948.635) by collision-induced dissociation. Full sequence coverage of CΨUCCGp$_{1135\text{-}1140}$ was achieved using the a, b, c, d, w, x, y and z ions and their derivatives. **(C)** The dissociated ions identified from the MS/MS signals for CΨUCCGp$_{1135\text{-}1140}$.

S5-5. Ψ1232 in 18S rRNA.

The RNase H fragments H5 from cellular 18S rRNA and *in vitro* transcribed reference ($^{13}C_{10}$-guanosine-labeled) 18S rRNA were purified by reversed-phase HPLC. The resulting “light” and “heavy” fragments were mixed at a 1: 1 ratio, digested with RNase T1, and subjected to LC-MS analysis. In principle, RNase T1 should produce three fragments, namely CCUGp$_{1230–1233}$, CCU*Gp$_{1230–1233}$ (*G, $^{13}C_{10}$-guanosine), and modified CCUGp$_{1230–1233}$ (CCΨGp$_{1230–1233}$), from the sequence 1230–1233 of each of the cellular and reference 18S rRNAs. **(A)** Extracted ion monitoring of CCUGp and its cognate fragment. The CCUGp-cognate fragments were obtained by RNase T1 digestion of the purified RNase H 1211–1435 fragment of 18S rRNA. In the LC-MS analysis, the fragment CCUGp$_{1230–1233}$ and the corresponding heavy fragment CCU*Gp$_{1230–1233}$ eluted at the same time. Oligonucleotide ion masses are indicated in the right. From the signal heights of the MS spectra of the light and heavy CCUGp ions, the stoichiometry of modification was estimated as 97%. **(B)** Identification of CCΨGp$_{1230–1233}$ and the site of pseudouridylation analyzed by database searching *via* the human rRNA sequences with Ariadne. CCΨGp$_{1230–1233}$ was separated from the RNase T1 digest of C/U-5,6-d2-labeled 18S rRNA. The MS/MS

spectrum was obtained for $[CC\Psi Gp]^{2-}$ (m/z 641.093) by collision-induced dissociation. Full sequence coverage of $CC\Psi Gp_{1230–1233}$ was achieved using the a, b, c, d, w, x, y and z ions and their derivatives. **(C)** The dissociated ions identified from the MS/MS signals for $CC\Psi Gp_{1230–1233}$.

S5-6. Um1760 and Ψ1768 in 28S rRNA.

Cellular 28S rRNA and *in vitro* transcribed reference ($^{13}C_{10}$-guanosine-labeled) 28S rRNA were purified by reversed-phase HPLC. The resulting "light" and "heavy" fragments were mixed at a 1: 1 ratio, digested with RNase T1, and subjected to LC-MS analysis.

(A) Extracted ion monitoring of AUCUCAACCUAUUCUCAAACUUUAAAUGp and its cognate fragment. By the LC-MS analysis, we determined five cognate fragments, namely AUCUCAACCUAUUCUCAAACUUUAAAU*$Gp_{1757-1784}$, and modified AUCUCAACCUAUUCUCAAACUUUAAAU$Gp_{1757-1784}$ (AUCUmCAACCΨAΨΨCUCAAACUUΨAAAU$Gp_{1757-1784}$, AUCUCAACCΨAΨΨCUCAAACUUΨAAAU$Gp_{1757-1784}$, AUCUmCAACCUAΨΨCUCAAACUUΨAAAU$Gp_{1757-1784}$ and AUCUCAACCUAΨΨCUCAAACUUΨAAAU$Gp_{1757-1784}$), from the sequence 1757-1784 of each of the cellular and reference 28S rRNAs. The fragment AUCUCAACCUAUUCUCAAACUUUAAAU$Gp_{1757-1784}$ was not detected. Most abundant masses of oligonucleotide ion are shown in the right. **(B)** Identification of AUCUmCAACCΨAΨΨC$OH_{1757-1770}$ and the sites of 2'-O-methylation and pseudouridylation analyzed by database searching *via* the human rRNA sequences with Ariadne. AUCUmCAACCΨAΨΨC$OH_{1757-1770}$ was separated from the RNase T1 and RNase H (by using h28S-1783-chimera oligonucleotide) digest of C/U-5,6-d2-labeled 28S rRNA. The MS/MS spectrum was obtained for $[AUCUmCAACC\Psi A\Psi\Psi COH]^{3-}$ (m/z 1445.898) by collision-induced dissociation. Full sequence coverage of AUCUmCAACCΨAΨΨC$OH_{1757-1770}$ was achieved using the a, b, c, d, w, x, y and z ions and their derivatives. **(C)** The dissociated ions identified from the MS/MS signals for AUCUmCAACCΨAΨΨC$OH_{1757-1770}$.

S5-7. Ψ2619 in 28S rRNA.

Cellular 28S rRNA and *in vitro* transcribed reference ($^{13}C_{10}$-guanosine-labeled) 28S rRNA were purified by reversed-phase HPLC. The resulting "light" and "heavy" fragments were mixed at a 1: 1 ratio, digested with RNase T1, and subjected to LC-MS analysis. In principle, RNase T1 should produce three fragments, namely UUCUCUUUUCUUU$Gp_{2612-2625}$, UUCUCUUUUCUUU*$Gp_{2612-2625}$ and modified UUCUCUUUUCUUU$Gp_{2612-2625}$

($UUCUCUU\Psi UCUUUGp_{2612-2625}$), from the sequence 2612-2625 of each of the cellular and reference 28S rRNAs. **(A)** Extracted ion monitoring of UUCUCUUUUCUUUGp and its cognate fragment. The UUCUCUUUUCUUUGp-cognate fragments were obtained by RNase T1 digestion of the purified 28S rRNA. In the LC-MS analysis, the fragment $UUCUCUUUUCUUUGp_{2612-2625}$ and the corresponding heavy fragment $UUCUCUUUUCUUU{*}Gp_{2612-2625}$ eluted at the same time. Oligonucleotide ion masses are shown in the right. The mass windows used for extraction were ±5 ppm. From the signal heights of the MS spectra of the light and heavy UUCUCUUUUCUUUGp ions, the stoichiometry of modification was estimated as 90%. **(B)** Identification of $UUCUCUU\Psi UCUUUGp_{2612-2625}$ and the site of pseudouridylation analyzed by database searching *via* the human rRNA sequences with Ariadne. $UUCUCUU\Psi UCUUUGp_{2612-2625}$ was separated from the RNase T1 digest of C/U-5,6-d2-labeled 28S rRNA. The MS/MS spectrum was obtained for $[UUCUCUU\Psi UCUUUGp]^{3-}$ (m/z 1453.526) by collision-induced dissociation. Full sequence coverage of $UUCUCUU\Psi UCUUUGp_{2612-2625}$ was achieved using the a, b, c, d, w, x, y and z ions and their derivatives. **(C)** The dissociated ions identified from the MS/MS signals for $UUCUCUU\Psi UCUUUGp_{2612-2625}$.

S5-8. Gm3606 in 28S rRNA.

Cellular 28S rRNA and *in vitro* transcribed reference ($^{13}C_9$-C/U-labeled) 28S rRNA were purified by reversed-phase HPLC. The resulting “light” and “heavy” rRNAs were mixed at a 1: 1 ratio, digested with RNase A, and subjected to LC-MS analysis. In principle, RNase A should produce namely three fragments, $AGGGGAAUp_{3603-3610}$, $AGGGGAA{*}Up_{3603-3610}$ (*U, $^{13}C_9$-uridine) and modified $AGGGGAAUp_{3603-3610}$ ($AGGGmGAAUp_{3603-3610}$), from the sequence 3603-3610 of each of the cellular and reference 28S rRNAs. **(A)** Extracted ion monitoring of AGGGGAAUp and its cognate fragment. The AGGGGAAUp-cognate fragments were obtained by RNase A digestion of the purified 28S rRNA. In the LC-MS analysis, the fragment $AGGGGAAUp_{3603-3610}$ and the corresponding heavy fragment $AGGGGAA{*}Up_{3603-3610}$ eluted at the same time. Oligonucleotide ion masses are shown in the right. The mass windows used for extraction were ±5 ppm. From the signal heights of the MS spectra of the light and heavy AGGGGAAUp ions, the stoichiometry of modification was estimated as 96%. **(B)** Identification of $AGGGmGAAUp_{3603-3610}$ and the site of 2'-O-methylation analyzed by database searching *via* the human rRNA sequences with Ariadne. $AGGGmGAAUp_{3603-3610}$ was separated from the RNase A digest of 28S rRNA. The MS/MS spectrum was obtained for $[AGGGmGAAUp]^{3-}$ (m/z 900.793) by collision-induced dissociation. Full sequence coverage of $AGGGmGAAUp_{3603-3610}$ was achieved using the a, b, c, d, w, x, y and z ions and their derivatives. **(C)** The dissociated ions identified from the

MS/MS signals for AGGGmGAAUp$_{3603\text{-}3610}$

S5-9. Ψ4463 in 28S rRNA.

The RNase H fragments H27 from cellular 28S rRNA and *in vitro* transcribed reference ($^{13}C_{10}$-guanosine-labeled) 28S rRNA were purified by reversed-phase HPLC. The resulting "light" and "heavy" fragments were mixed at a 1: 1 ratio, digested with RNase T1, and subjected to LC-MS analysis. In principle, RNase T1 should produce five fragments, namely UUGp$_{4462\text{-}4464}$, UU*Gp$_{4462\text{-}4464}$ and modified UUGp$_{4462\text{-}4464}$ (UΨGp$_{4462\text{-}4464}$, UUGmGp$_{4462\text{-}4465}$, and UΨGmGp$_{4462\text{-}4465}$), from the sequence 4462-4464 of each of the cellular and reference 28S rRNAs. **(A)** Extracted ion monitoring of UUGp and its cognate fragment. The UUGp-cognate fragments were obtained by RNase T1 digestion of the purified RNase H fragment H27 of 28S rRNA. In the LC-MS analysis, the fragment UUGp$_{4462\text{-}4464}$ and the corresponding heavy fragment UU*Gp$_{4462\text{-}4464}$ eluted at the same time. Oligonucleotide ion masses are shown in the right. The mass windows used for extraction were ±5 ppm. From the peak areas of the MS chromatogram of the each fragment, the stoichiometry of modification at Ψ4463 was estimated as 17%. **(B)** Identification of UΨGmGp$_{4462\text{-}4465}$ and the site of pseudouridylation analyzed by database searching *via* the human rRNA sequences with Ariadne. UΨGmGp$_{4462\text{-}4465}$ was separated from the RNase T1 digest of C/U-5,6-d2-labeled 28S rRNA. The MS/MS spectrum was obtained for [UΨGmGp]$^{2-}$ (m/z 667.589) by collision-induced dissociation. Full sequence coverage of UΨGmGp$_{4462\text{-}4465}$ was achieved using the a, b, c, d, w, x, y and z ions and their derivatives. **(C)** The dissociated ions identified from the MS/MS signals for UΨGmGp$_{4462\text{-}4465}$.

Supplementary Figure S6. Potential base-pairing interactions between candidate guide snoRNAs and 3 ribose 2'-O-methylation and 7 pseudouridylation sites in human 18S/28S rRNAs found in this study.

To predict box CD snoRNA targeting each 2′-O-methylation site, a sequence of 20 nucleotides centered on the modification site was extracted and examined by Snoscan software (Science. 1999;283(5405):1168-71, http://lowelab.ucsc.edu/snoscan/) using the snoRNA sequences compiled in snoRNA Atlas database (Nucleic Acids Res. 2016;44(11):5068-82, http://snoatlas.bioinf.uni-leipzig.de/index.php). Box ACA snoRNA targeting each pseudouridylation site, on the other hand, was predicted by the in-house program (Nucleic Acids Res. 2016;44(18):8951-8961) using the snoRNA sequences in snoRNA Atlas database. **(A)** Each 2′-O-methylation guiding snoRNA and its base paring to rRNA around Cm621 (A1) in 18S rRNA and Um1760 (A2) and Gm3606 (A3) in 28S rRNA are indicated. **(B)** Each Ψ guiding snoRNA and its base paring to rRNA around Ψ897 (B1),

Ψ1045 (B2), Ψ1136 (B3) and Ψ1232 (B4) in 18S rRNA, and Ψ1768 (B5), Ψ2619 (B6) and Ψ4463 (B7) in 28S rRNA are indicated. The snoRNA and rRNA sequences are shown in the upper and lower strands, respectively. The D-, D'-, H- and ACA-boxes are boxed and the hairpin domains are schematized with a solid line. Modified nucleoside is indicated as blue letter with nucleoside number. **−**, regular hydrogen bonding; •, GU pairing.

Supplementary Figure S7. 3D maps of modified nucleotides in the *E. coli* (*Ec*), budding yeast (*Saccharomyces cerevisiae*, *Sc*)], and human (*Hs*) rRNAs.
The figure indicates the positions of 2'-O-methylated nucleotides (red), pseudouridines (yellow), and base-modified nucleotides (blue) in the 3D structures of the *Ec*, *Sc*, and *Hs* rRNAs (4YBB.pdb, 3U5B.pdb and 4UG0.pdb).

Supplementary Figure S8. Distribution of PTM sites within the *S. cerevisiae* (upper graphs) and human (lower graphs) rRNAs.
The sequence distance from a given PTM site to the nearest neighbor PTM site is plotted against its frequency. A bin size of two was used for the frequency distribution. The left graphs use an ordinal scale, and the right graphs use a logarithmic scale.

Supplementary Figure S1. Taoka *et al*

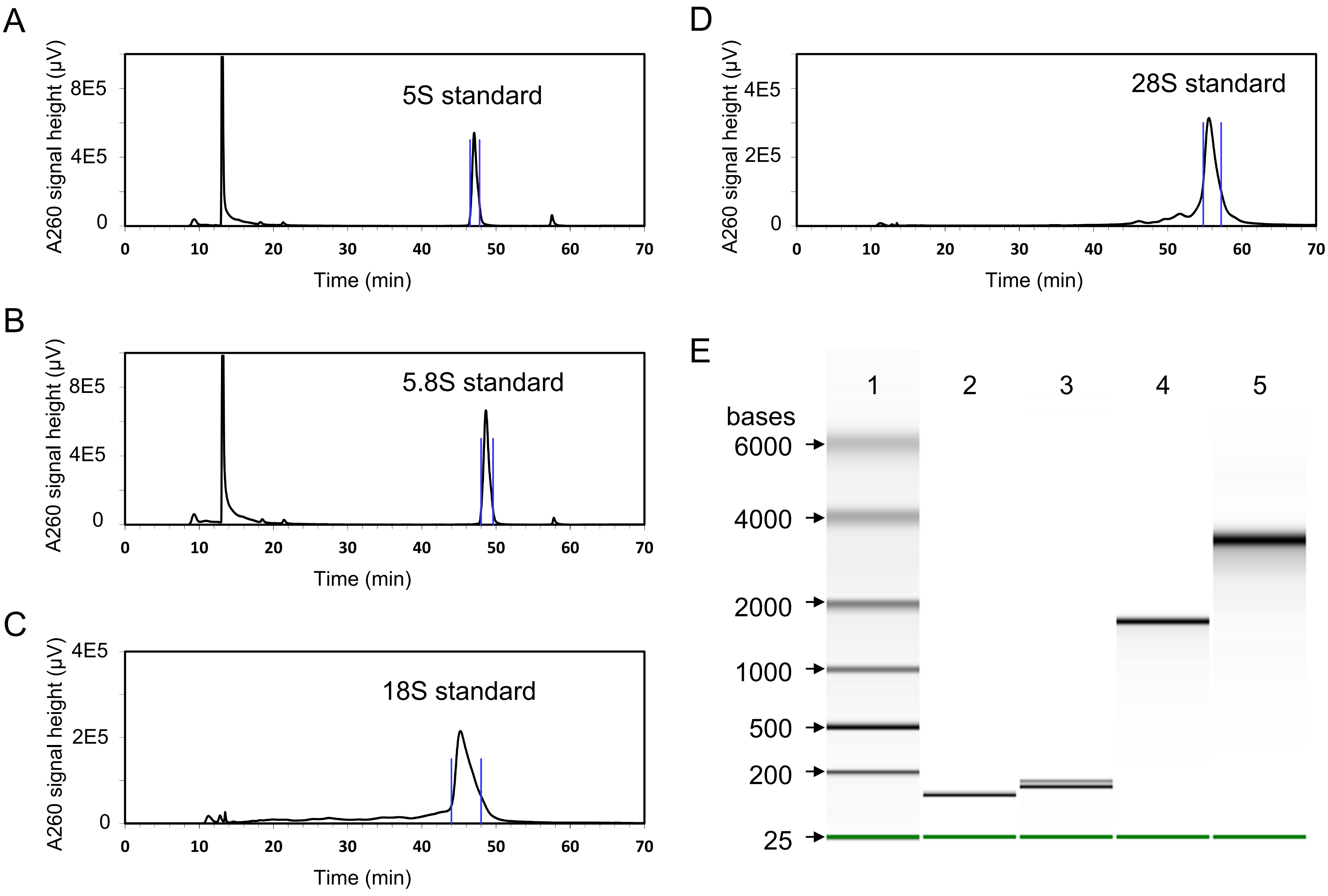

18S rRNA

1 UACCUGGUUGAUCCUGCCAGUAGCAUΛUGCUUGPCPCAAAGAUUAAGCCAUGCAUGUCUA

Fragment H1

61 AGUACGCACGGCCGGUACAGUGAAACUGCGAAPGGCUCΛUUAAAPCAGPUAUGGUJCCPU

Fragment H1

121 JGGUCGCUCGCUCCUCUCCUACUUGGAUAACUGUGGUAΛUUCUAGΛGCUAAJABAUGCCG

Fragment H1

181 ACGGGCGCUGACCCCCUUCGCGGGGGGAPGCGUGCAPUUAUCAGAUCAAAACCAACCCG

Fragment H1

241 GUCAGCCCCUCUCCGGCCCCGGCCGGGGGGCGGGCGCCGGCGGCUUUGGUGACUCPAGAU

Fragment H1

301 AACCUCGGGCCGAUCGCACGCCCCCCGUGGCGGCGACGACCCAUUCGAACGUCJGCCCUA

Fragment H1

Fragment H2

361 UCAACUUUCGAUGGUAGUCGCCGUGCCUACCAUGGUGACCACGGGPGACGGGGAAUCAGG

Fragment H2

421 GUUCGAUJCCGGAGA#GGAGCCUGAGAAACGGCUACCACAUBCAAGGΛAGGCAGCAGGCG

Fragment H2

Supplementary Figure S2-*continued*

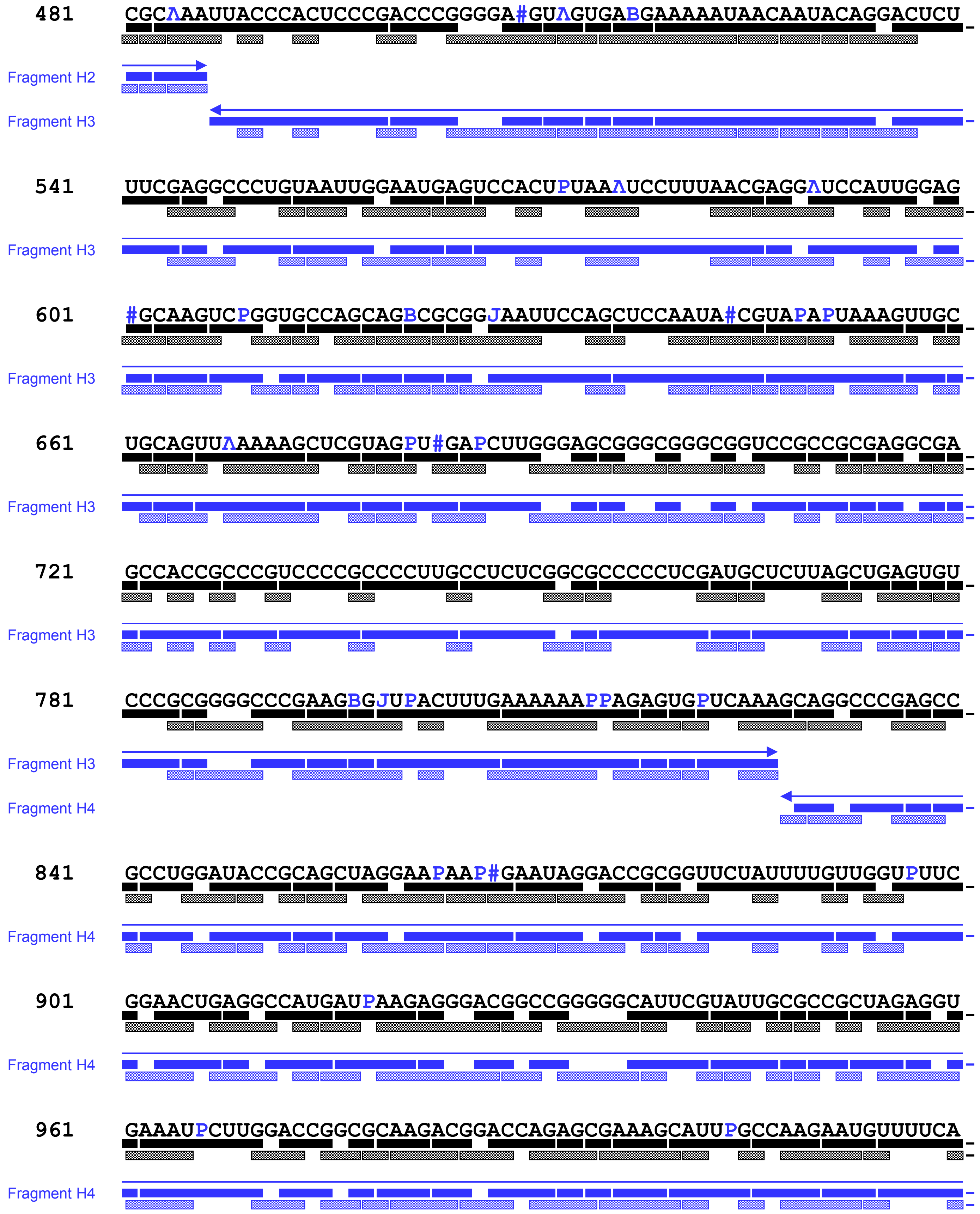

Supplementary Figure S2-*continued*

1021 UUAAUCAAGAΛCGAAAGUCGGAGGPPCGAAGACGAPCAGAUACCGUCGUAGUUCCGACCA

Fragment H4

1081 PAAACGAUGCCGACCGGCGAUGCGGCGGCGUUAUUCCCAUGACCCGCCGGGCAGCPUCCG

Fragment H4

1141 GGAAACCAAAGUCUUUGGGUUCCGGGGGGAGUAPGGPUGCAAAGCUGAAACUUAAAGGAA

Fragment H4

1201 UUGACGGAAGGGCACCACCAGGAGUGGAGCCPGCGGCPUAAUUPGACαCAACACGGGAAA

Fragment H4

Fragment H5

1261 CCUCACCCGGCBCGGACACGGACAGGAJUGACAGAUUGAUAGCUCUUUCUCGAUUCCGUG

Fragment H5

1321 GGUGGJG#UGCAUGGCMGUUCUUAGUPGGUGGAGCGAUUUGUCUGGPUAAUUCCGAUAAC

Fragment H5

1381 GAΛCGAGACUBUGGCAUGCUAACUAGUUACGCGACCCCCGAGCGGUCGGCGUCCCCCAAC

Fragment H5

Fragment H6

1441 UJCUPA#AGGGACAAGUGGCGUUCAGCCACCCGAGAUUGAGCAAUAACA#GUCUGUGAUG

Fragment H6

1501 CCCUUAGAUGUCCGGGGCUGCACGCGCGCUACACUGACUGGCUCAGCGUGUGCCUACCCU

Fragment H6

Supplementary Figure S2-*continued*

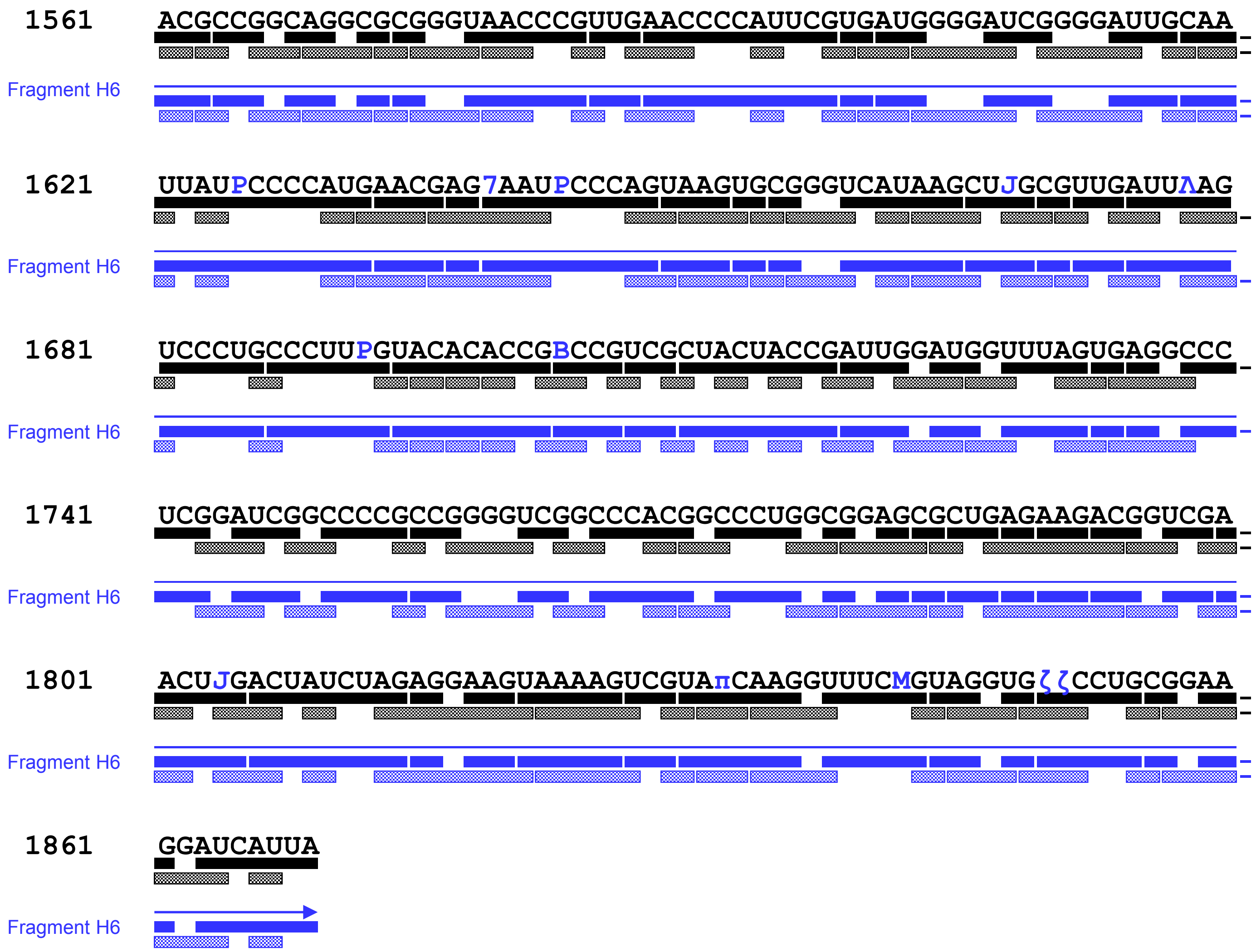

28S rRNA

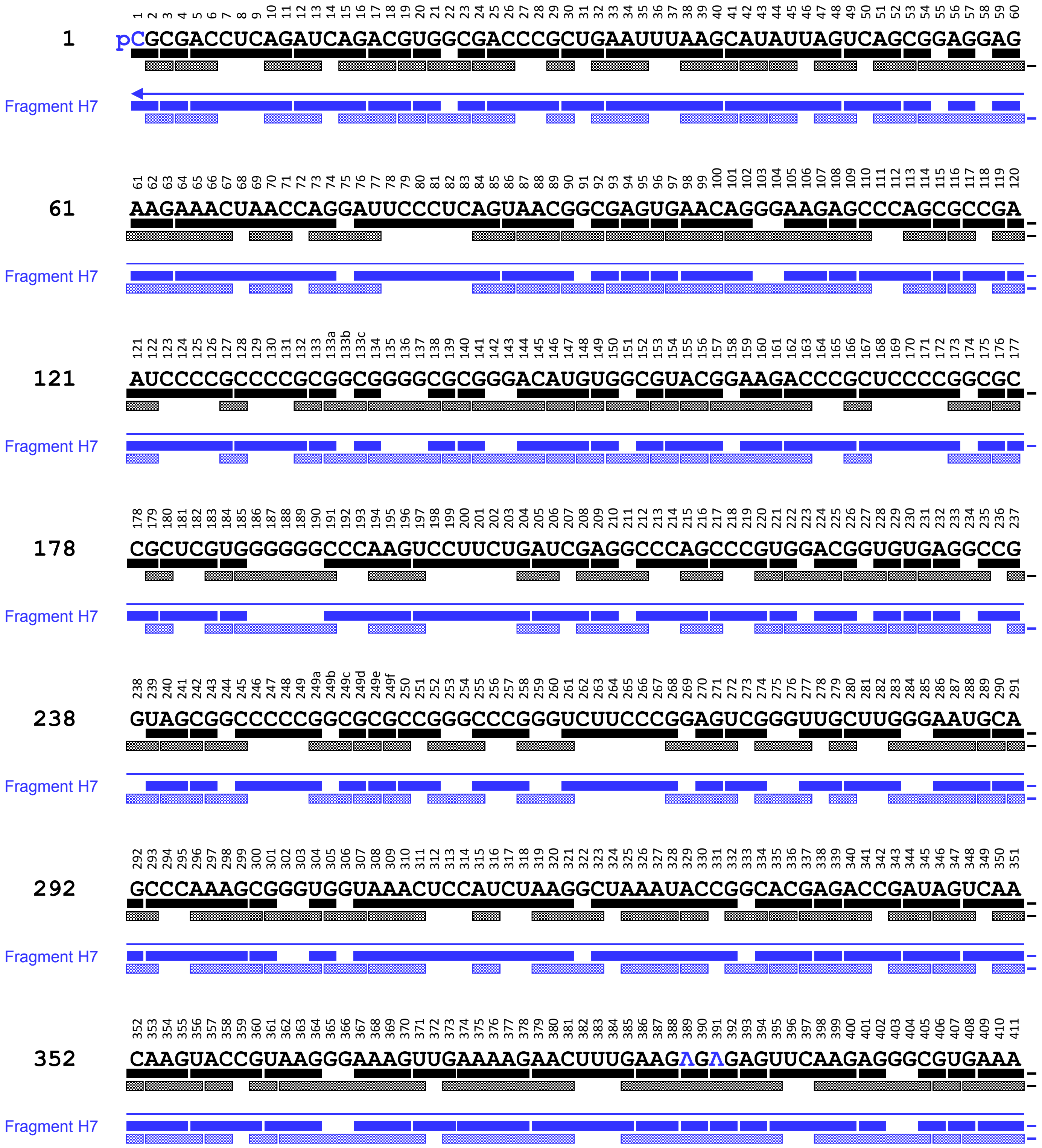
1 pCGCGACCUCAGAUCAGACGUGGCGACCCGCUGAAUUUAAGCAUAUUAGUCAGCGGAGGAG
Fragment H7
61 AAGAAACUAACCAGGAUUCCCUCAGUAACGGCGAGUGAACAGGGAAGAGCCCAGCGCCGA
Fragment H7
121 AUCCCCGCCCCGCGGCGGGGCGCGGGACAUGUGGCGUACGGAAGACCCGCUCCCCGGCGC
Fragment H7
178 CGCUCGUGGGGGGCCCAAGUCCUUCUGAUCGAGGCCCAGCCCGUGGACGGUGUGAGGCCG
Fragment H7
238 GUAGCGGCCCCCGGCGCGCCGGGCCCGGGUCUUCCCGGAGUCGGGUUGCUUGGGAAUGCA
Fragment H7
292 GCCCAAAGCGGGUGGUAAACUCCAUCUAAGGCUAAAUACCGGCACGAGACCGAUAGUCAA
Fragment H7
352 CAAGUACCGUAAGGGAAAGUUGAAAAGAACUUUGAAGΛGΛGAGUUCAAGAGGGCGUGAAA
Fragment H7

Supplementary Figure S3-*continued*

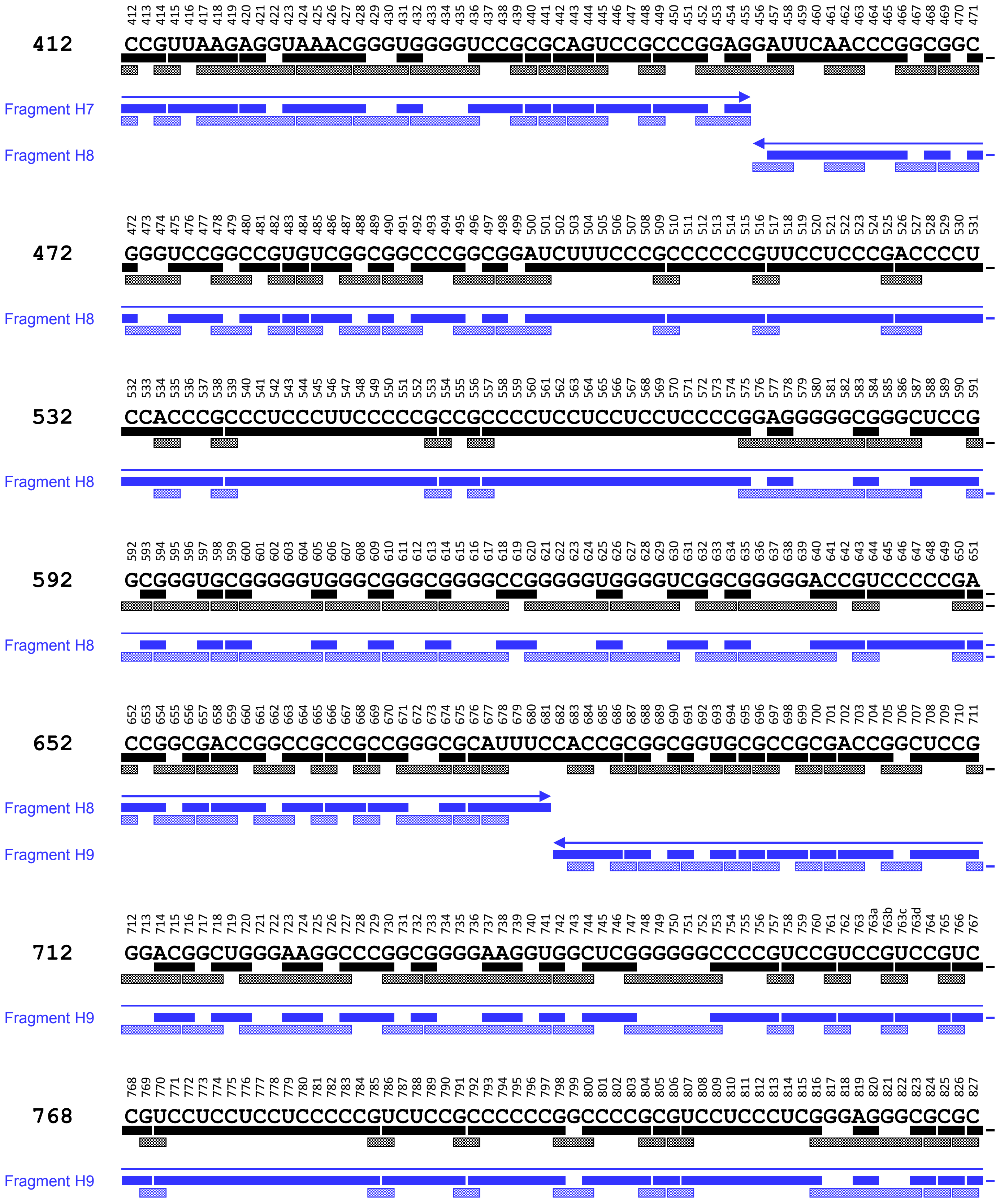

Supplementary Figure S3-*continued*

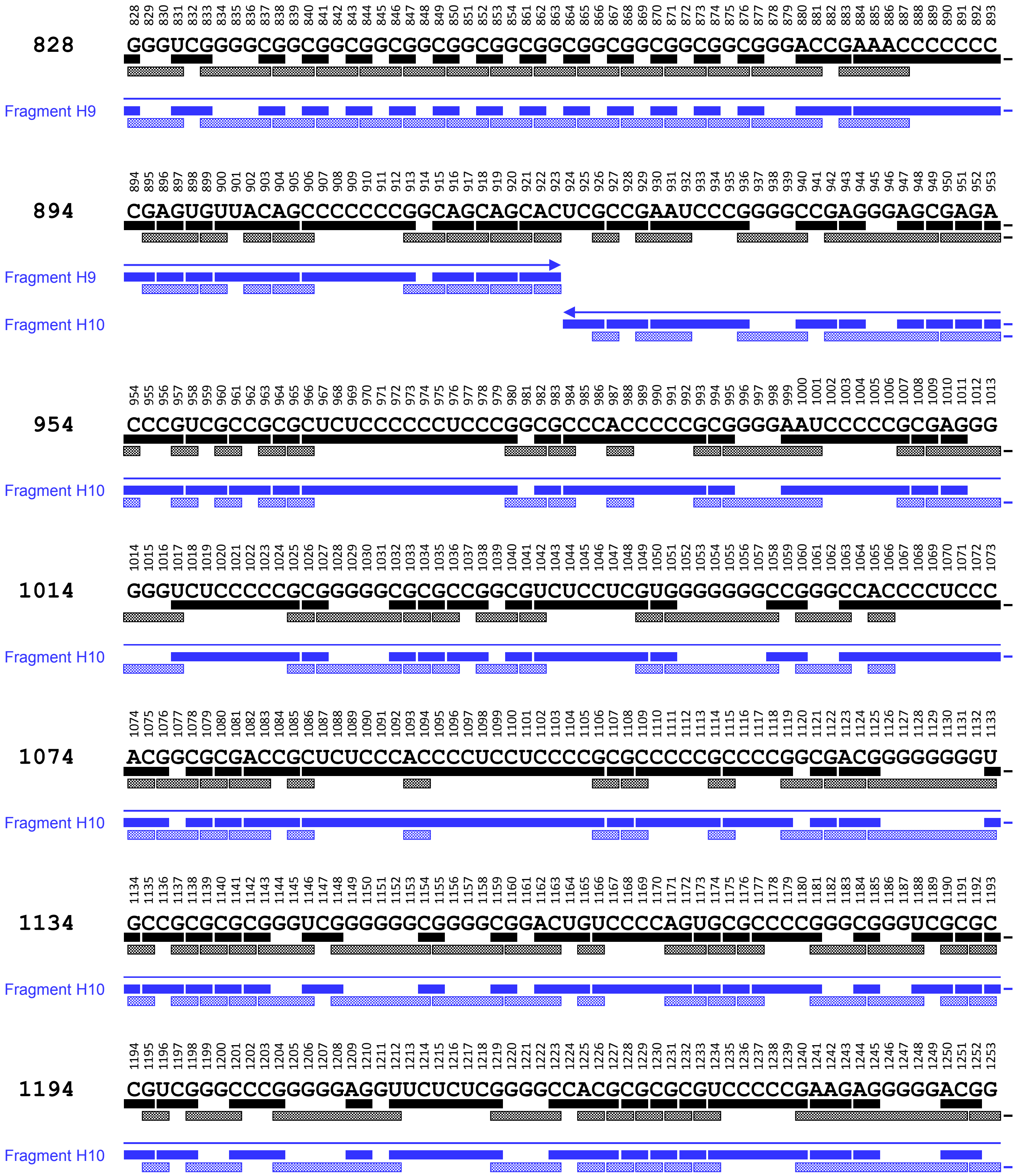

Supplementary Figure S3-*continued*

1254 CGGAGCGAGCGCACGGGGUCGGCGGCGACGUCGGCUACCCACCCGACCC#UCUUGΠΛACΛ

Fragment H10

1314 CGGACCAAGGAGUBUAACACGUGCGCGAGUCGGGGGCUCGCACGAAAGCCGCCGUGGCGC

Fragment H10

Fragment H11

1374 AAUGAAGGUGAAGGCCGGCGCGCUCGCCGGCCGAGGUGGGAUCCCGAGGCCUCUCCAGUC

Fragment H11

1434 CGCCGAGGGCGCACCACCGGCCCGUCUCGCCCGCCGCGCCGGGGAGGUGGAGCACGAGCG

Fragment H11

1494 CACGUGUUAGGACCC#AΛAGAUGGUGAΛCPAUGCCUGGGCAGGGCGAAGCCAGAGGAAAC

Fragment H11

1554 UCUGGUGGAGGUCCGPAGCGGUCCUGACGUGCAAAUCGGUCGUCCGACCUGGGUAUAG#G

Fragment H11

1614 GCGAAAGACUAAUCGAACCAUCUAGUAGCUGGUUCCCUCCGAAGUUUCCCPCAGGAPAGC

Fragment H11

Supplementary Figure S3-*continued*

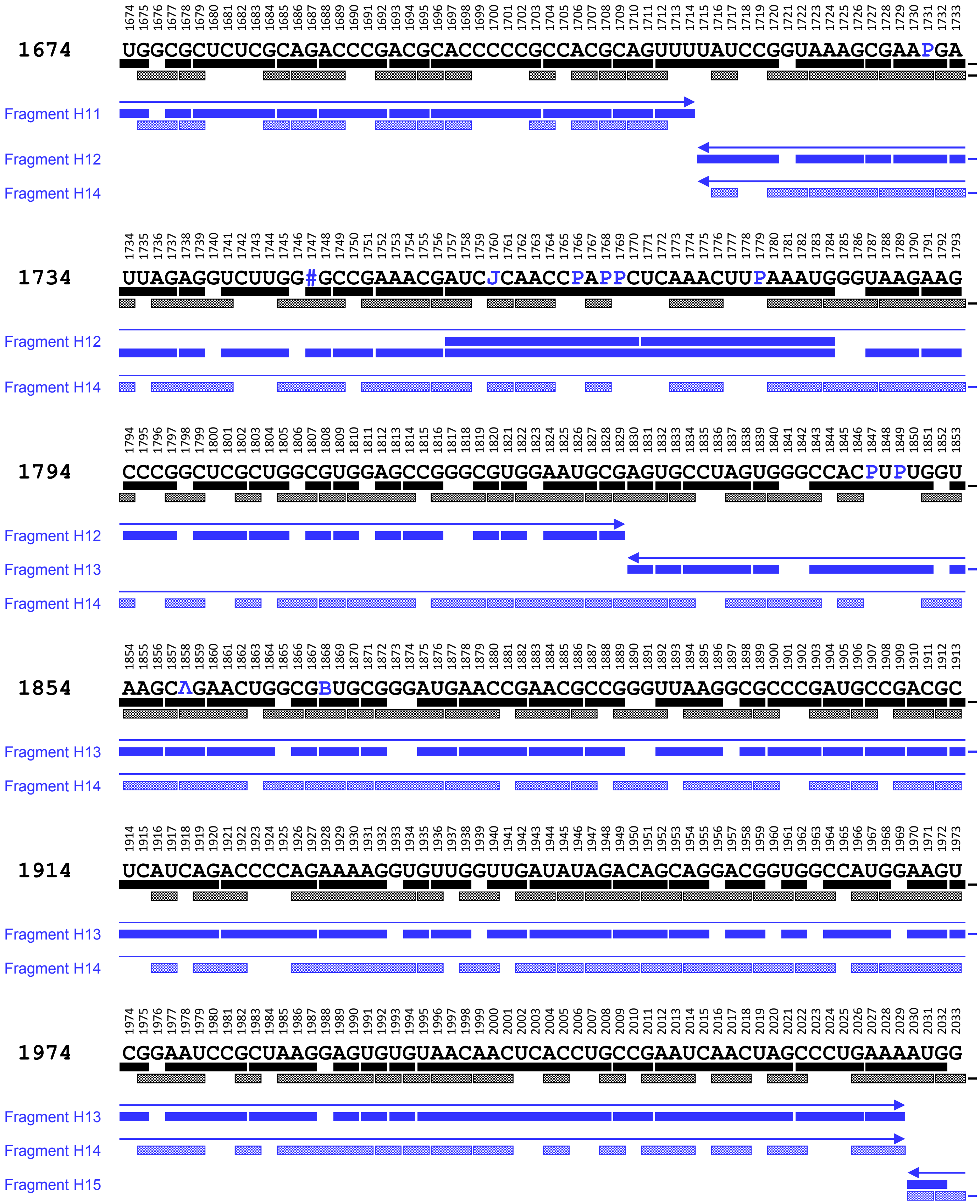

Supplementary Figure S3-*continued*

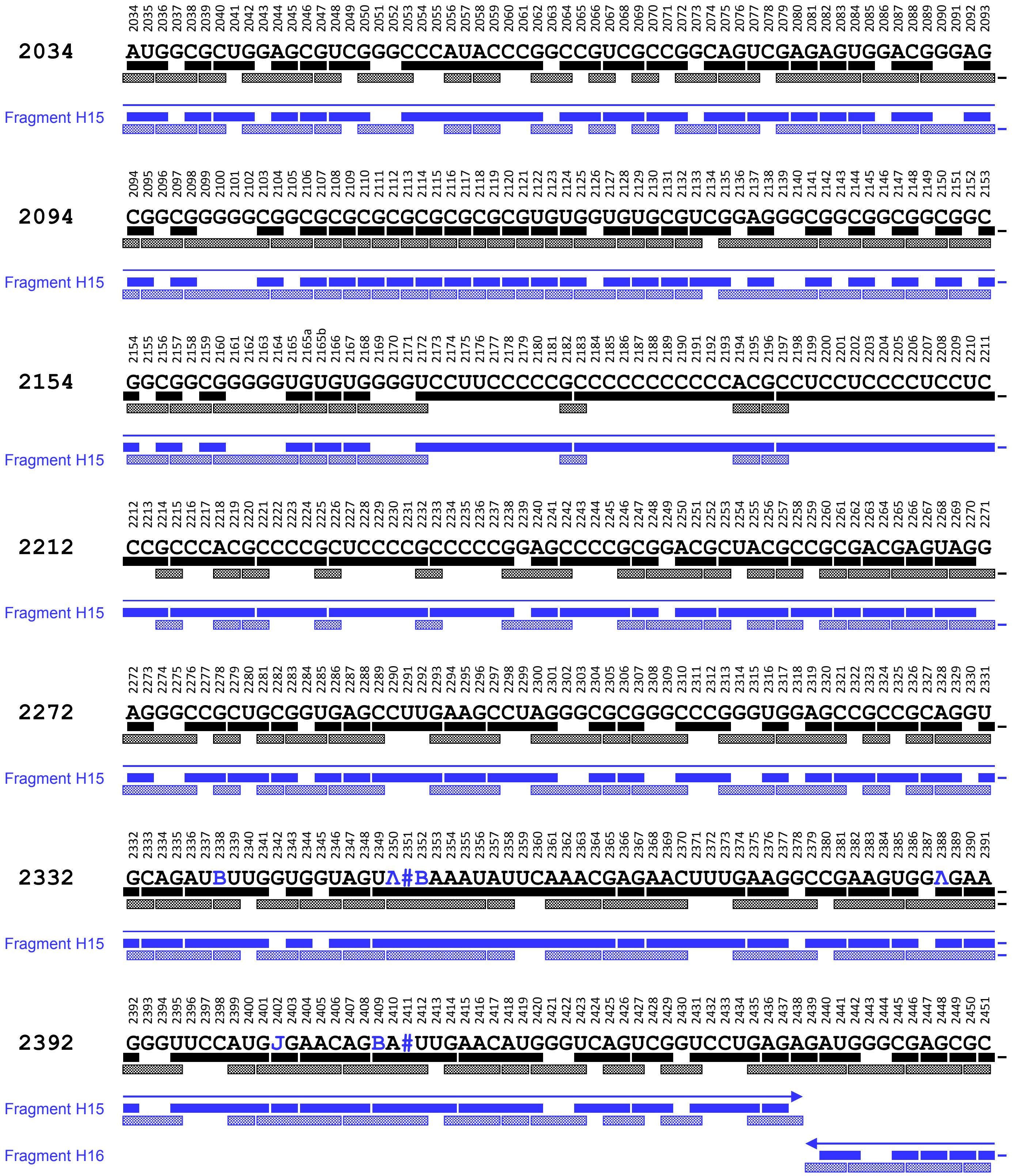

2452 **CGUUCCGAAGGGACGGGCGAUGGCCUCCGUUGCCCUCGGCCGAPCGAAAGGGAGUCGGGU**

Fragment H16

2512 **UCAGAUCCCCGAAUCCGGAGUGGCGGAGAUGGGCGCCGCGAGGCGUCCAGUGCGGUAACG**

Fragment H16

2572 **CGACCGAUCCCGGAGAAGCCGGCGGGAGCCCCGGGGAGAGUUCUCUUPUCUUUGUGAAGG**

Fragment H16

2632 **GCAGGGCGCCCUGGAAUGGGUUCGCCCCGAGAGAGGGGCCCGUGCCUUGGAAAGCGUCGC**

Fragment H16

2692 **GGUUCCGGCGGCGUCCGGUGAGCUCUCGCUGGCCCUUGAAAAUCCGGGGGAGAGGGUGUA**

Fragment H16

2752 **AAUCUCGCGCCGGGCCGUACCCΛUAUCCGCAGCAGGUCUBCAAGGUGAACΛGCCUCUGGB**

Fragment H16

Fragment H17

2812 **AUGUUGGAACAAJGPAGGPAAGGGAAGUCGGCAAGCBGGAUCCGUAACUUC#GGAUAAGG**

Fragment H17

Supplementary Figure S3-*continued*

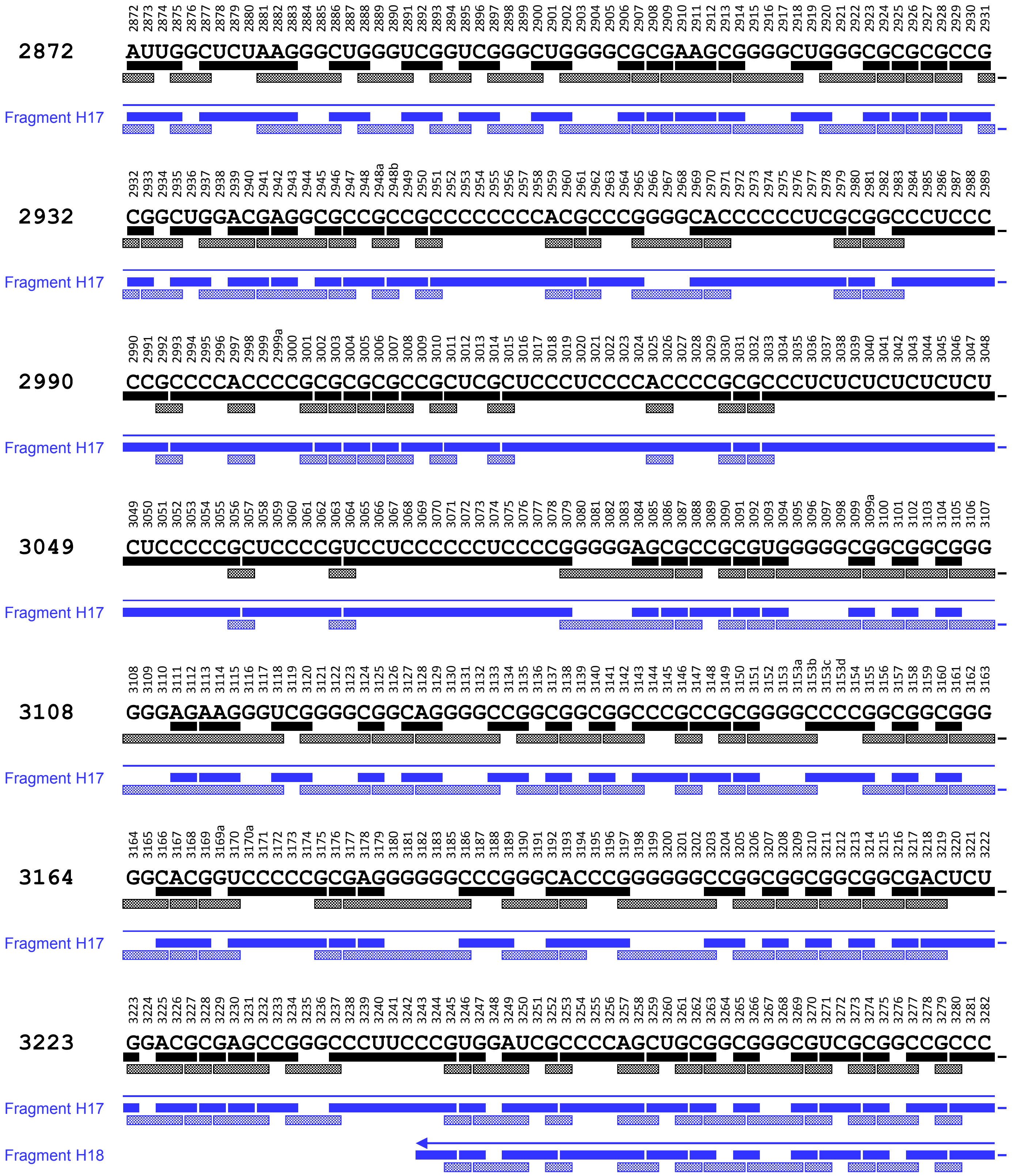

Supplementary Figure S3-*continued*

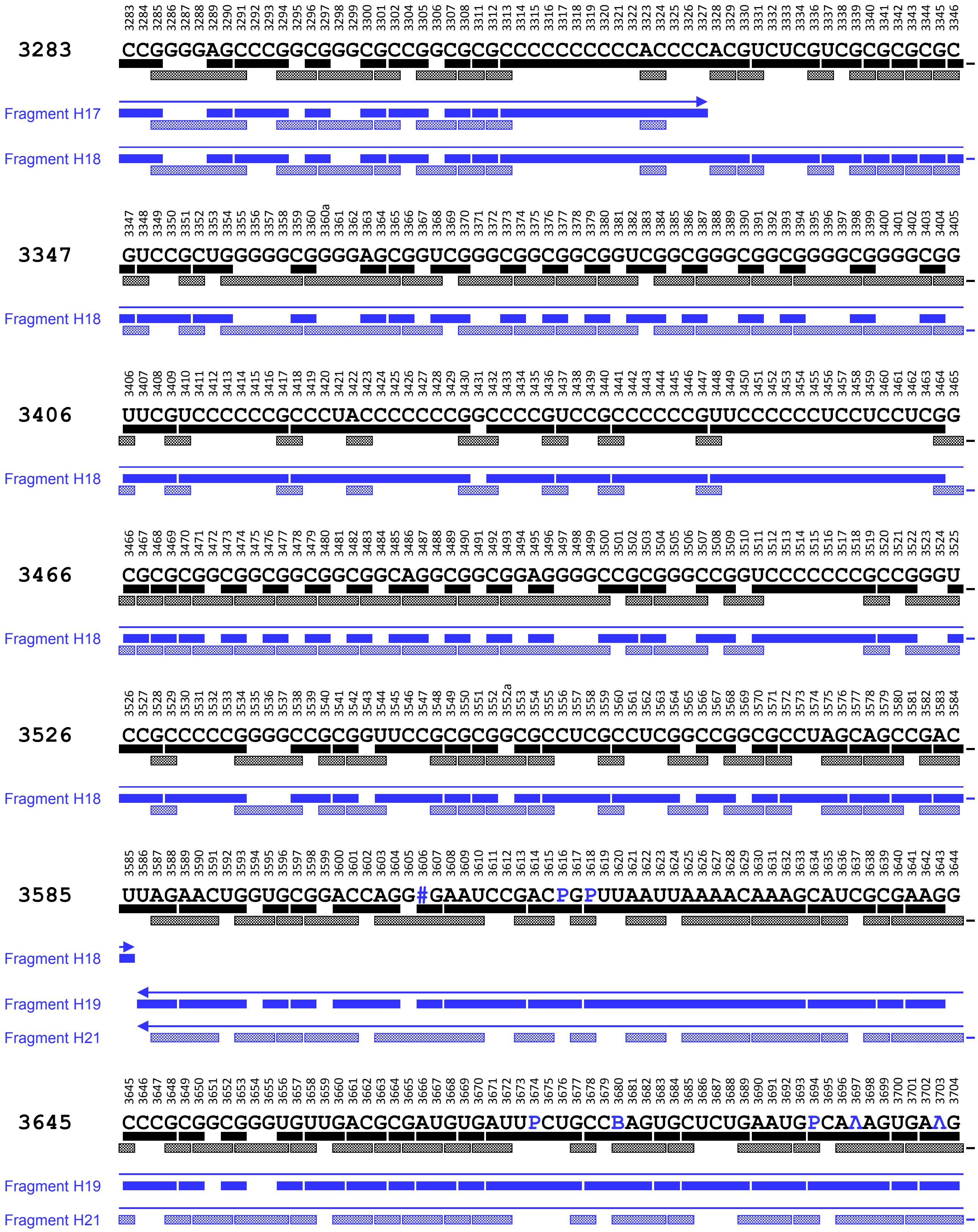

Supplementary Figure S3-*continued*

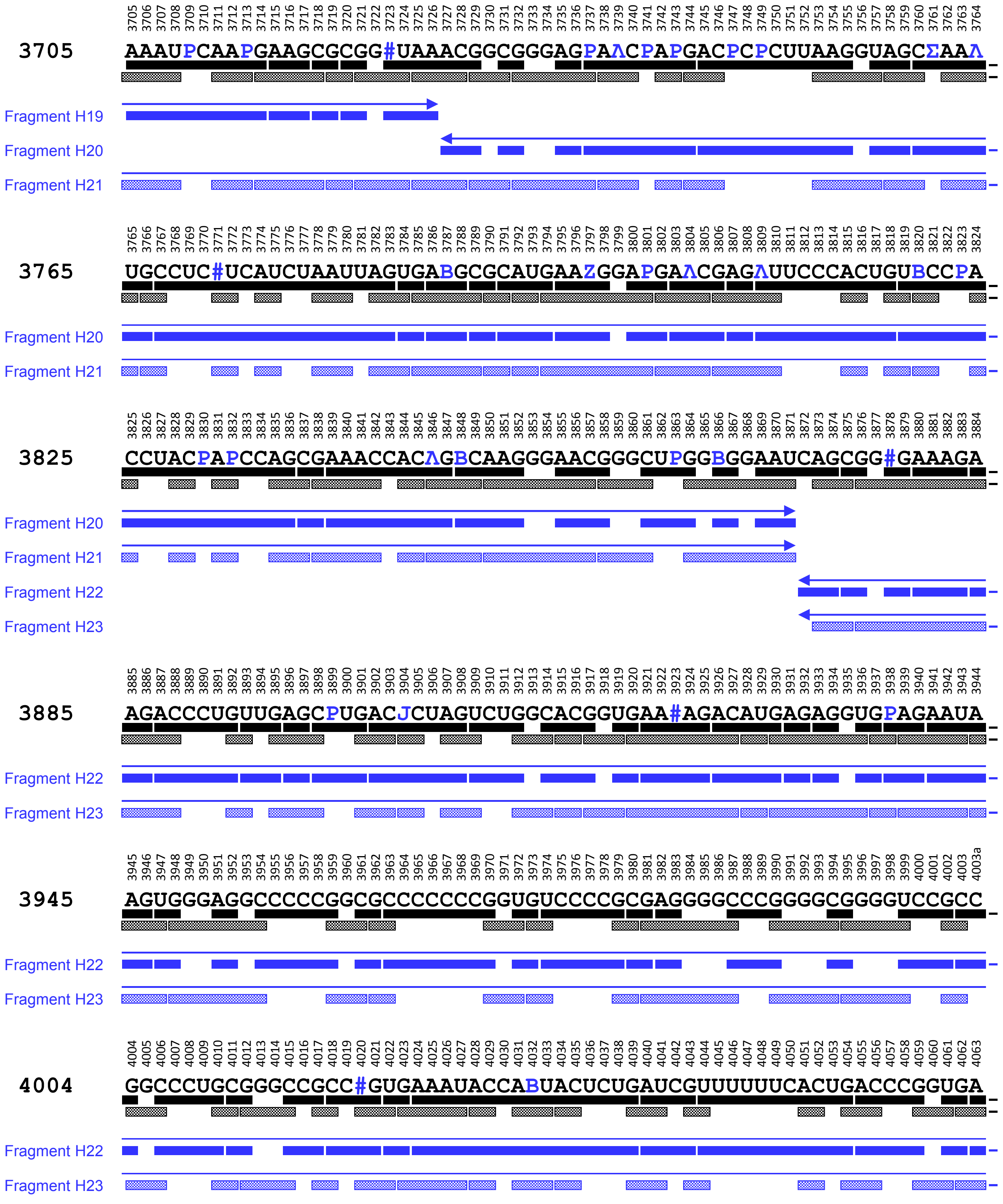

Supplementary Figure S3-*continued*

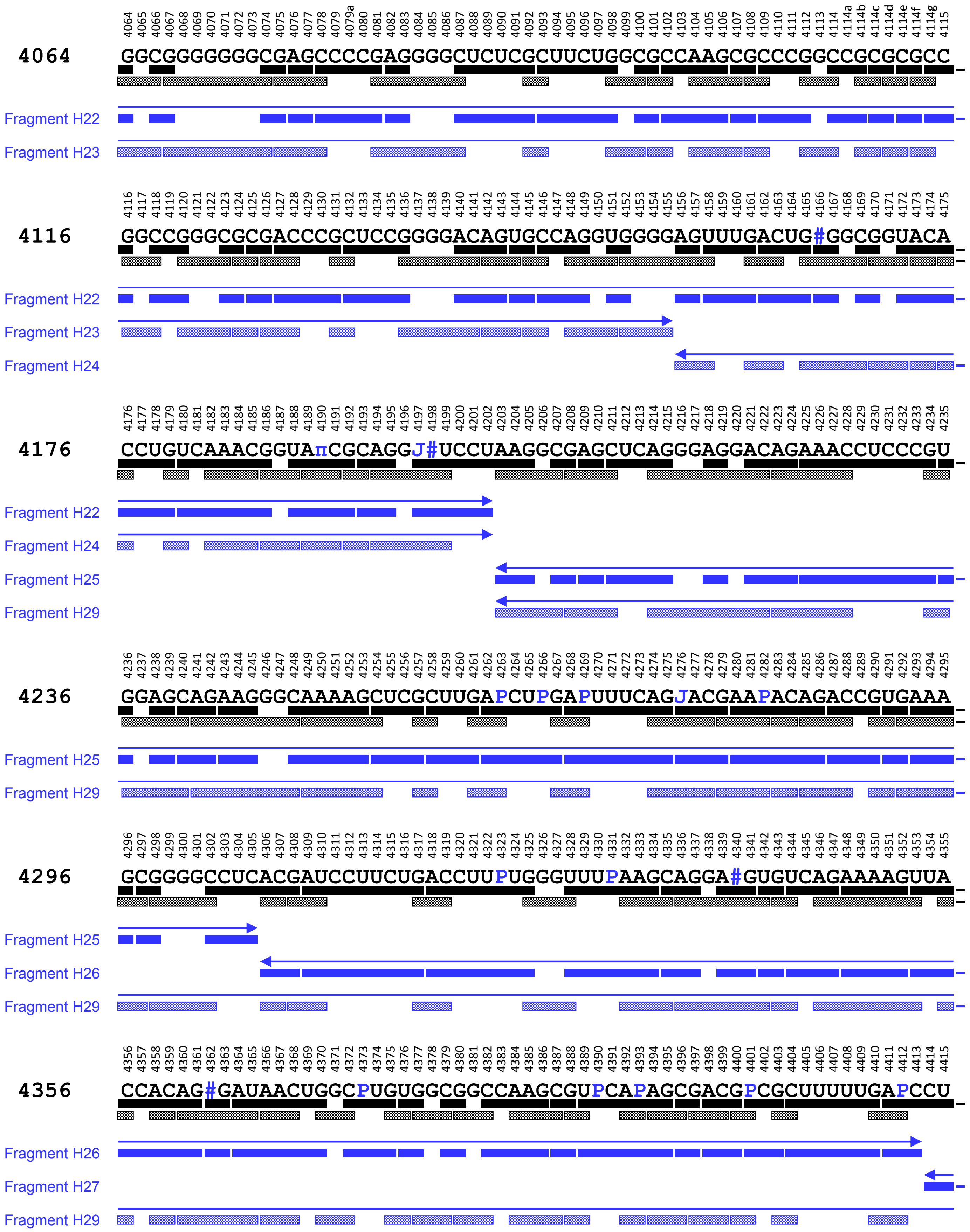

Supplementary Figure S3-*continued*

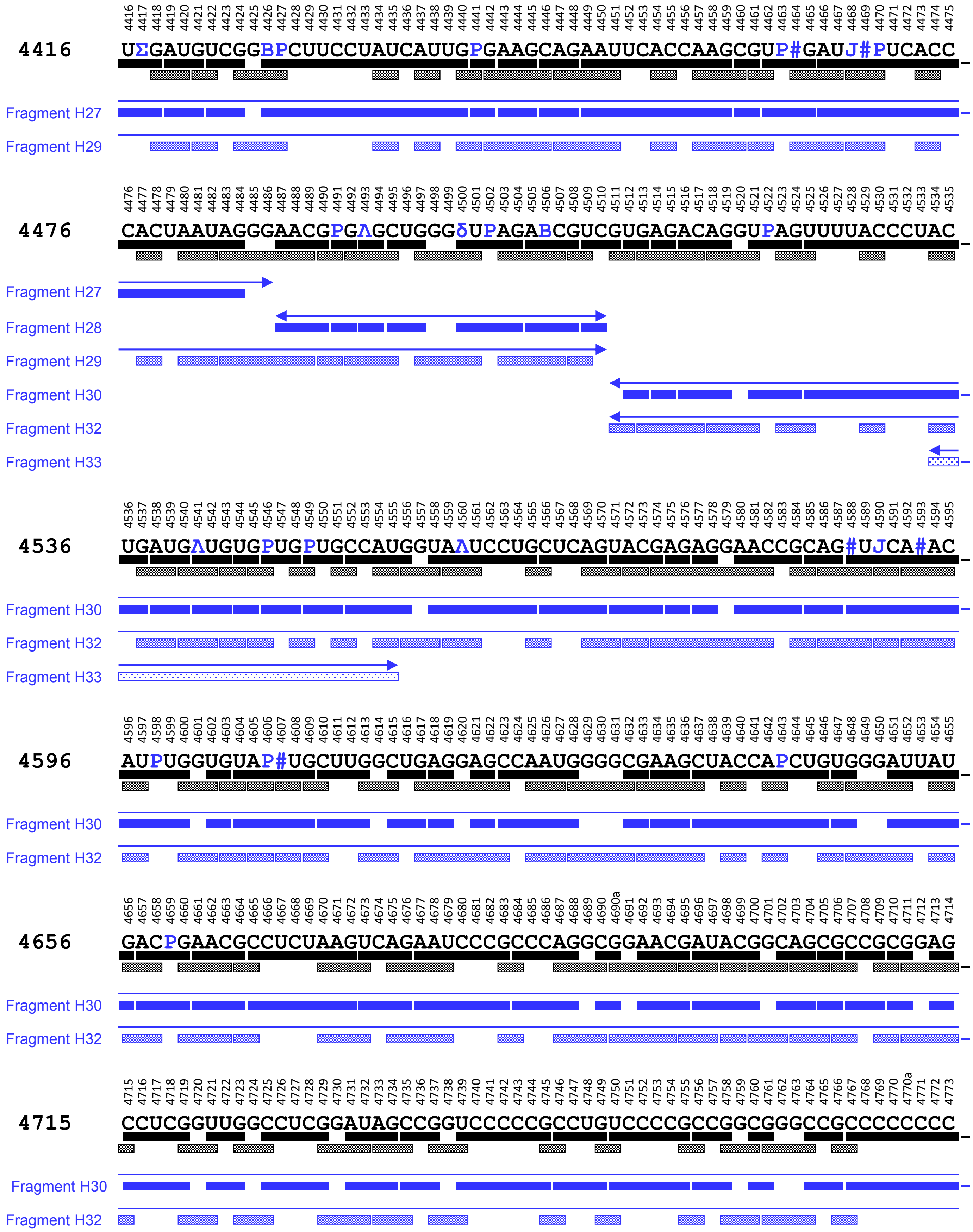

Supplementary Figure S3-*continued*

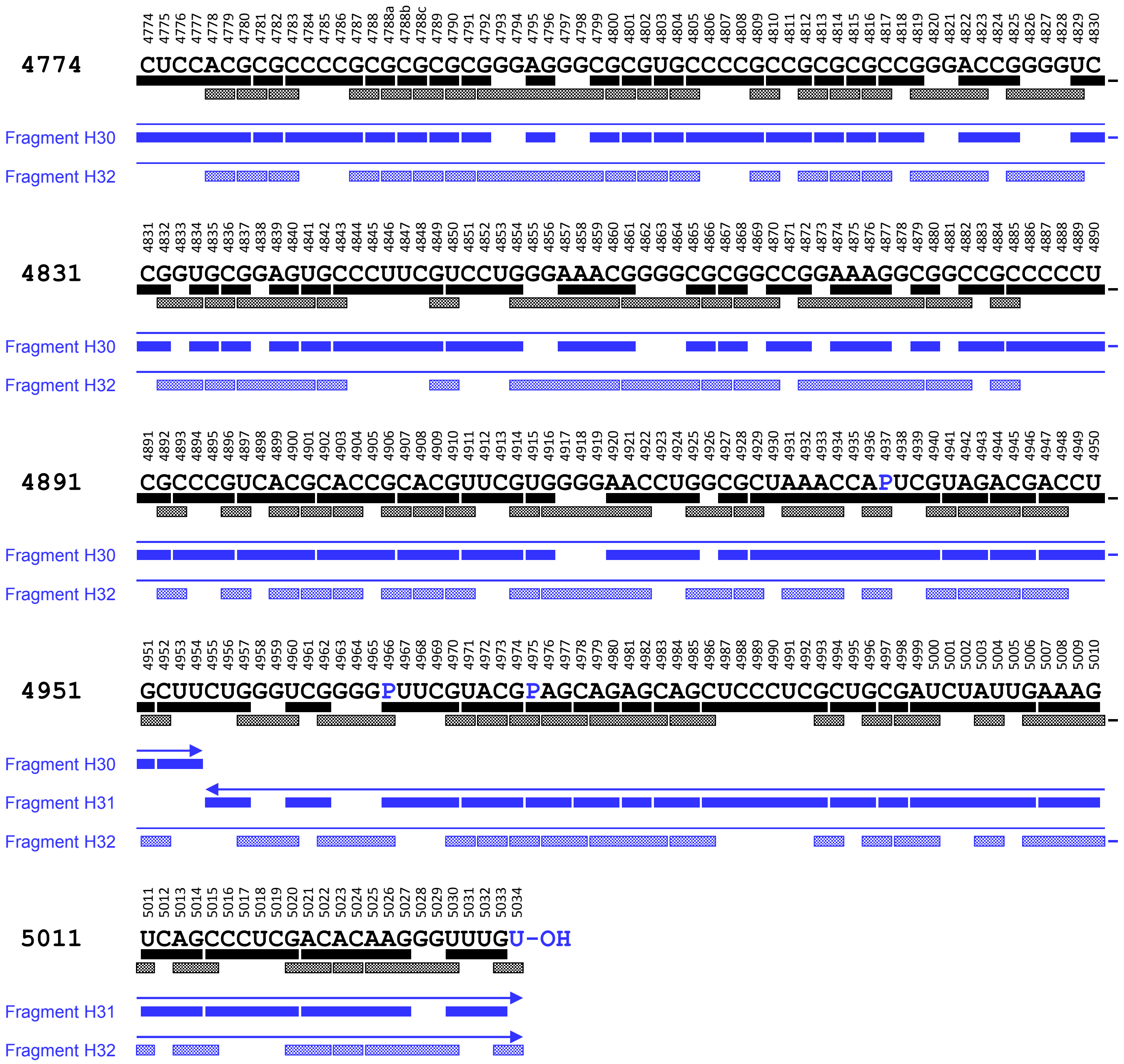

5S rRNA

```
  1 pppGUCUACGGCCAUACCACCCUGAACGCGCCCGAUCUCGUCUGAUCUCGGAAGCUAAGCAGG
 61     GUCGGGCCUGGUUAGUACUUGGAUGGGAGACCGCCUGGGAAUACCGGGUGCUGUAGGCUU-OH
```

5.8S rRNA

```
  1   pCGACUCUUAGCGGJGGAUCACUCGGCUCGUGCGUCGAUGAAGAACGCAGCUAGCPGCGAG
 61     AAUUAAUGPGAAUU#CAGGACACAUUGAUCAUCGACACUUCGAACGCACUUGCGGCCCCG
121     GGUUCCUCCCGGGGCUACGCCUGUCUGAGCGUCGCUU-OH
```

Supplementary Figure S4-1. Taoka *et al*

A

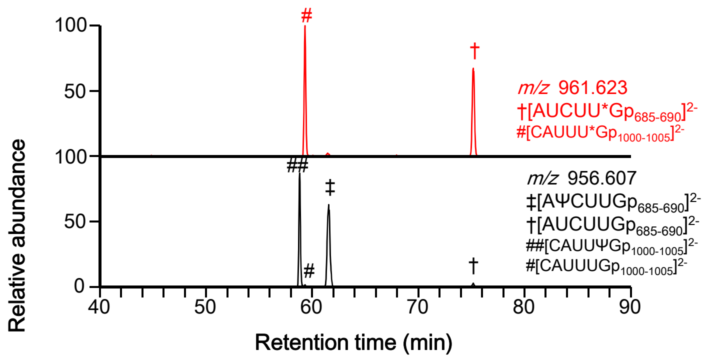

B

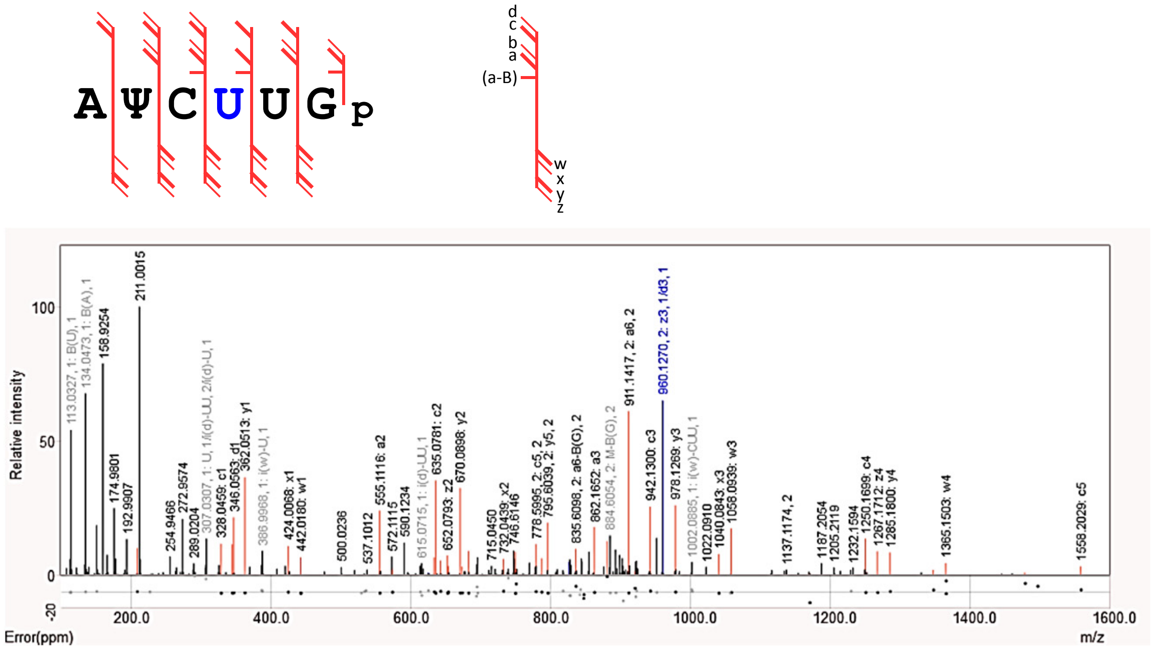

C

#	a-B	(a-B)2-	a	a2-	b	b2-	c	c2-	d	d2-	seq.	w	w2-	x	x2-	y	y2-	z	z2-	#
5'											OH									5'
1	113.024	56.009	248.079	123.536	266.090	132.541	328.045	163.519	346.056	172.524	A									6
2	---	---	555.111	277.052	573.121	286.057	635.077	317.035	653.087	326.040	Ψ	1672.178	835.586	1654.168	826.580	1592.212	795.602	1574.201	786.597	5
3	749.109	374.051	862.164	430.579	880.175	439.584	942.131	470.562	960.141	479.567	C	1365.147	682.070	1347.136	673.064	1285.180	642.087	1267.170	633.081	4
4	1056.162	527.578	1170.202	584.598	1188.213	593.603	1250.169	624.581	1268.179	633.586	U	1058.093	528.543	1040.082	519.538	978.126	488.560	960.116	479.554	3
5	1364.200	681.597	1478.240	738.616	1496.251	747.622	1558.206	778.600	1576.217	787.605	U	750.055	374.524	732.044	365.519	670.089	334.541	652.078	325.535	2
6	1672.238	835.615	1823.288	911.140	1841.298	920.145					G	442.017	220.505	424.007	211.500	362.051	180.522	344.040	171.516	1
3'											P									3'

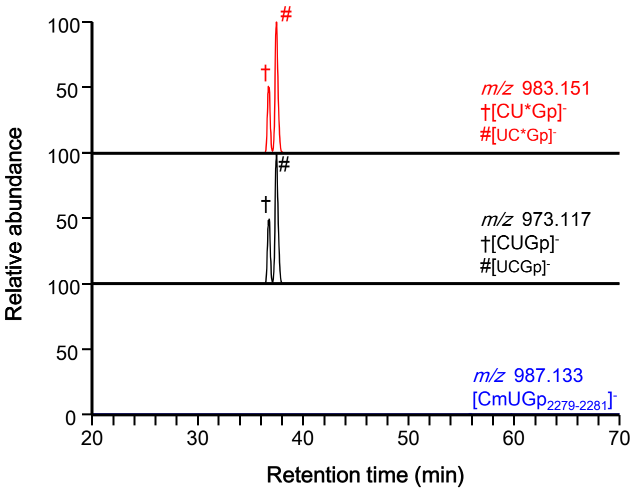

100
50
100
50
100
50
0
Relative abundance
†
#
m/z 983.151
†[CU*Gp]-
#[UC*Gp]-
m/z 973.117
†[CUGp]-
#[UCGp]-
m/z 987.133
[CmUGp2279-2281]-
20
30
40
50
60
70
Retention time (min)

A

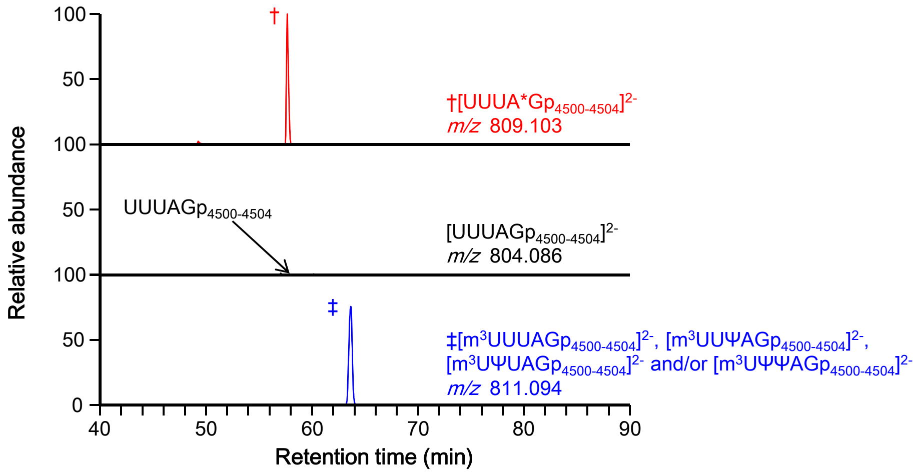

B

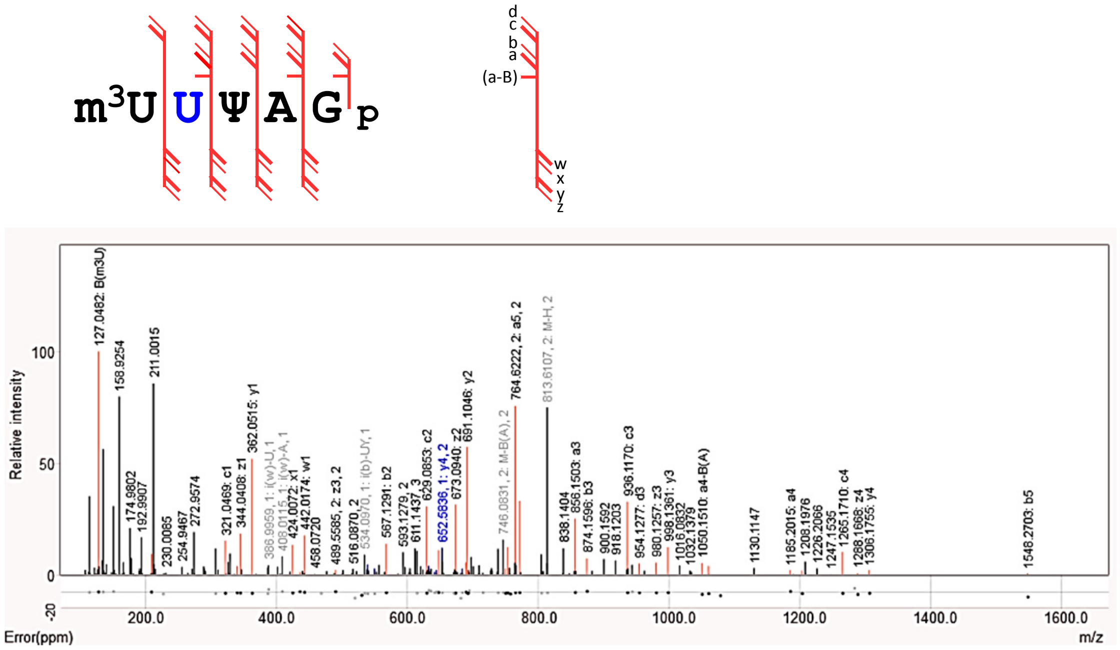

C

#	a-B	(a-B)$^{2-}$	a	a^{2-}	b	b^{2-}	c	c^{2-}	d	d^{2-}	seq.	w	w^{2-}	x	x^{2-}	y	y^{2-}	z	z^{2-}	#
5'											OH									5'
1	113.024	56.009	241.080	120.036	259.091	129.042	321.046	160.020	339.057	169.025	m^{3}U									5
2	435.078	217.035	549.118	274.055	567.128	283.061	629.084	314.038	647.095	323.044	U	1386.139	692.566	1368.128	683.561	1306.173	652.583	1288.162	643.577	4
3	---	---	856.149	427.571	874.160	436.576	936.116	467.554	954.126	476.560	Ψ	1078.101	538.547	1060.091	529.542	998.135	498.564	980.124	489.559	3
4	1050.147	524.570	1185.202	592.097	1203.212	601.103	1265.168	632.081	1283.179	641.086	A	771.070	385.031	753.059	376.026	691.103	345.048	673.093	336.043	2
5	1379.200	689.096	1530.249	764.621	1548.260	773.626					G	442.017	220.505	424.007	211.500	362.051	180.522	344.040	171.516	1
3'											P									3'

Supplementary Figure S5-1. Taoka *et al*

A

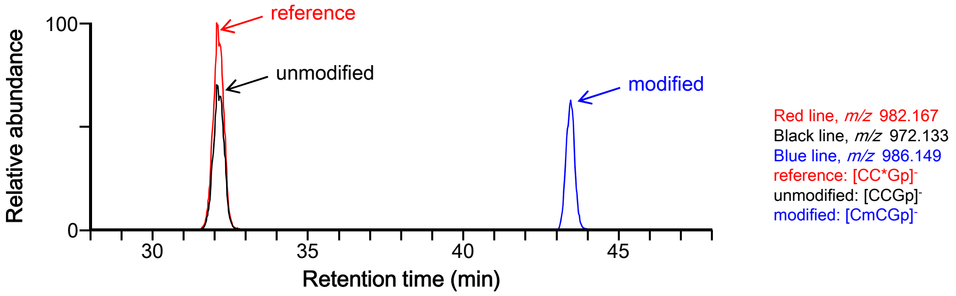

B

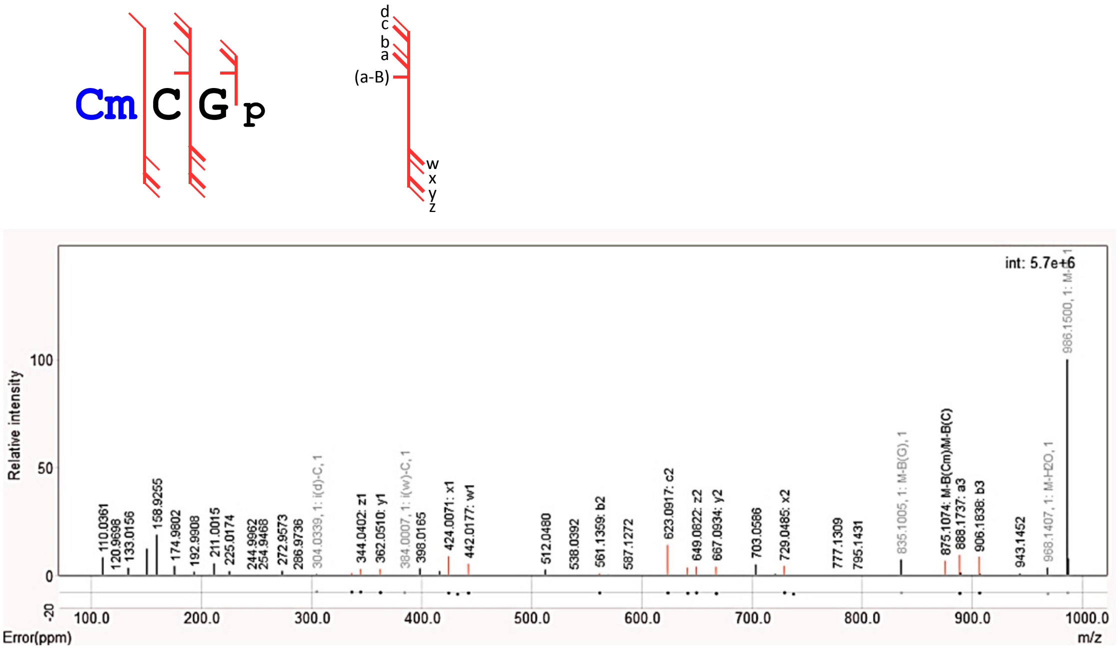

C

#	a-B	a	b	c	d	seq.	w	x	y	z	#
5'						OH					5'
1	127.040	238.083	256.094	318.050	336.060	Cm					3
2	432.081	543.125	561.135	623.091	641.102	C	747.058	729.048	667.092	649.081	2
3	737.123	888.172	906.183			G	442.017	424.007	362.051	344.040	1
3'						P					3'

A

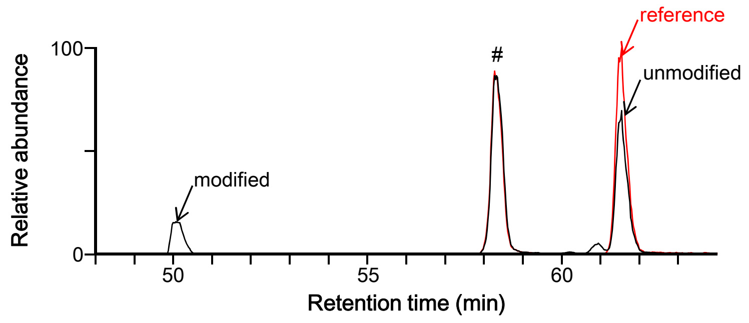

Red line, *m/z* 950.110
Black line, *m/z* 945.093
reference, [UUUUC*Gp]$^{2-}$
unmodified, [UUUUCGp]$^{2-}$
modified, [UΨUUCGp]$^{2-}$
#, [UCUUU*Gp]$^{2-}$ and [UCUUUGp]$^{2-}$ from the sequence 1152-1157of 18S rRNAs

B

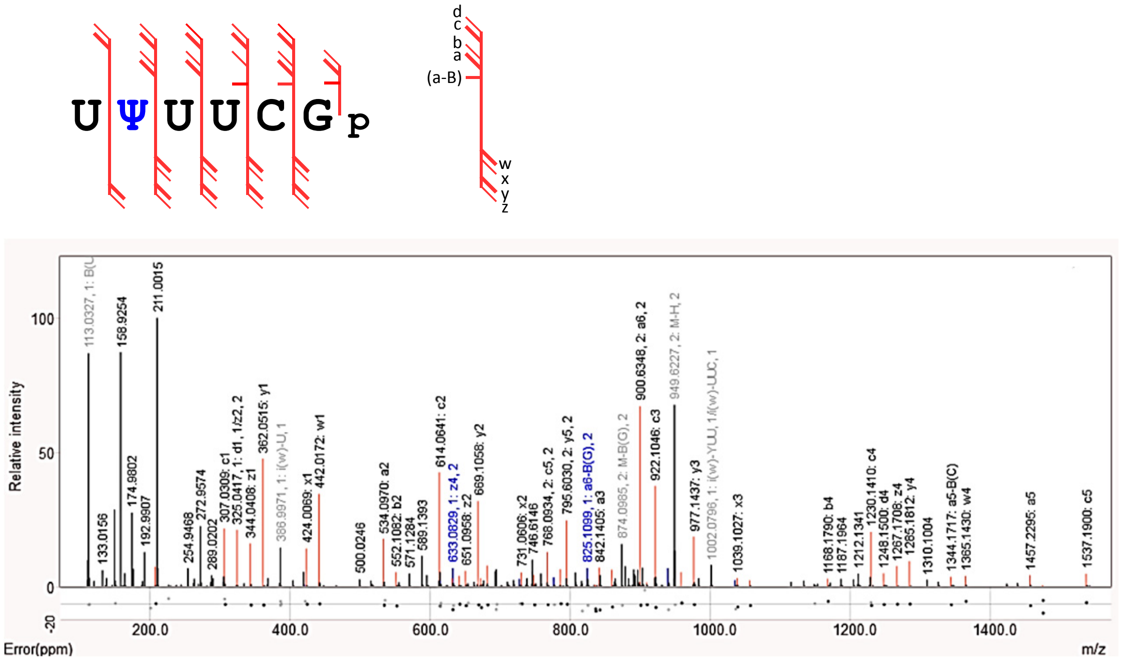

C

#	a-B	(a-B)$^{2-}$	a	a^{2-}	b	b^{2-}	c	c^{2-}	d	d^{2-}	seq.	w	w^{2-}	x	x^{2-}	y	y^{2-}	z	z^{2-}	#
5'											OH									5'
1	113.024	56.009	227.064	113.029	245.075	122.034	307.031	153.012	325.041	162.017	U									6
2	---	---	534.096	266.544	552.106	275.550	614.062	306.527	632.073	315.533	Ψ	1672.178	835.586	1654.168	826.580	1592.212	795.602	1574.201	786.597	5
3	728.094	363.543	842.134	420.563	860.144	429.569	922.100	460.546	940.111	469.552	U	1365.147	682.070	1347.136	673.064	1285.180	642.087	1267.170	633.081	4
4	1036.132	517.562	1150.172	574.582	1168.182	583.587	1230.138	614.565	1248.148	623.571	U	1057.109	528.051	1039.098	519.046	977.142	488.068	959.132	479.062	3
5	1344.170	671.581	1457.225	728.109	1475.236	737.114	1537.192	768.092	1555.202	777.098	C	749.071	374.032	731.060	365.027	669.105	334.049	651.094	325.043	2
6	1651.223	825.108	1802.273	900.633	1820.283	909.638					G	442.017	220.505	424.007	211.500	362.051	180.522	344.040	171.516	1
3'											P									3'

Supplementary Figure S5-3. Taoka *et al*

A

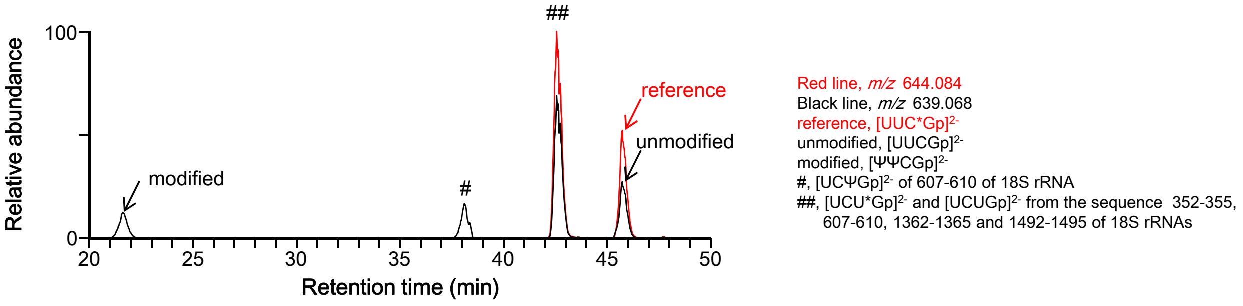

B

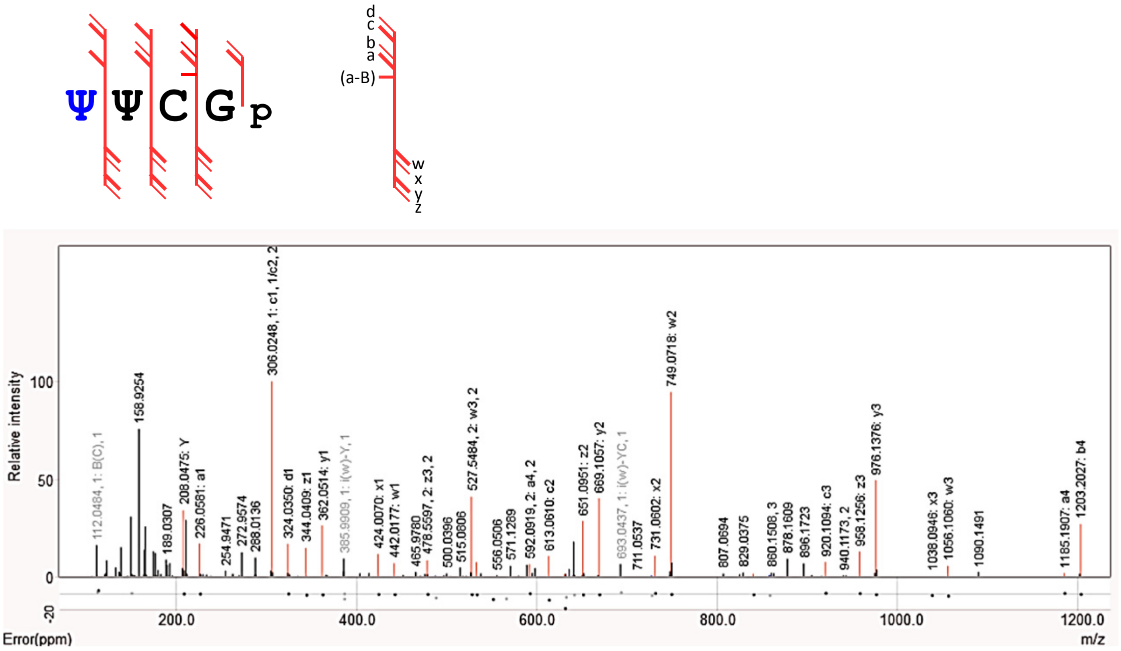

C

#	a-B	(a-B)$^{2-}$	a	a^{2-}	b	b^{2-}	c	c^{2-}	d	d^{2-}	seq.	w	w^{2-}	x	x^{2-}	y	y^{2-}	z	z^{2-}	#
5'											OH									5'
1	---	---	226.058	112.525	244.069	121.531	306.024	152.509	324.035	161.514	Ψ									4
2	---	---	533.090	266.041	551.100	275.046	613.056	306.024	631.066	315.030	Ψ	1056.103	527.548	1038.092	518.542	976.136	487.564	958.126	478.559	3
3	727.088	363.040	840.143	419.568	858.154	428.573	920.110	459.551	938.120	468.557	C	749.071	374.032	731.060	365.027	669.105	334.049	651.094	325.043	2
4	1034.141	516.567	1185.191	592.092	1203.201	601.097					G	442.017	220.505	424.007	211.500	362.051	180.522	344.040	171.516	1
3'											P									3'

Supplementary Figure S5-4. Taoka *et al*

A

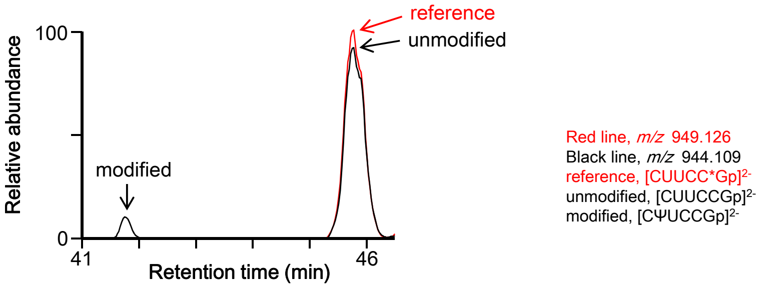

B

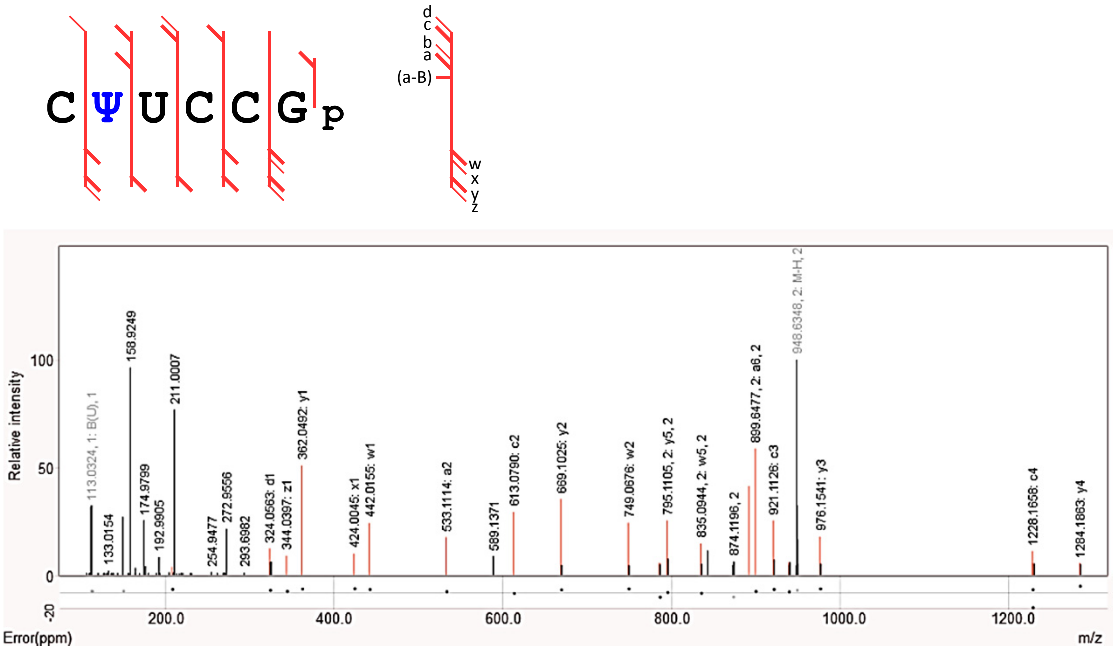

C

#	a-B	(a-B)$^{2-}$	a	a^{2-}	b	b^{2-}	c	c^{2-}	d	d^{2-}	seq.	w	w^{2-}	x	x^{2-}	y	y^{2-}	z	z^{2-}	#
5'											OH									5'
1	113.024	56.009	226.080	112.537	244.091	121.542	306.047	152.520	**324.057**	161.525	C									6
2	---	---	**533.112**	266.052	551.122	275.058	**613.078**	306.035	631.089	315.041	Ψ	1671.194	**835.093**	1653.184	826.088	1591.228	**795.110**	1573.217	**786.105**	5
3	727.110	363.051	841.150	420.071	859.160	429.077	**921.116**	460.054	**939.127**	469.060	U	1364.163	681.578	1346.152	672.572	**1284.196**	641.595	1266.186	632.589	4
4	1035.148	517.070	1148.204	573.598	1166.214	582.603	**1228.170**	613.581	1246.180	622.587	C	1056.125	527.559	1038.114	518.553	**976.158**	487.576	958.148	478.570	3
5	1342.202	670.597	1455.257	727.125	1473.268	736.130	1535.224	767.108	1553.234	776.114	C	**749.071**	374.032	731.060	365.027	**669.105**	334.049	651.094	325.043	2
6	1649.255	824.124	1800.305	**899.649**	1818.315	908.654					G	**442.017**	220.505	**424.007**	211.500	**362.051**	180.522	**344.040**	171.516	1
3'											P									3'

Supplementary Figure S5-5. Taoka *et al*

A

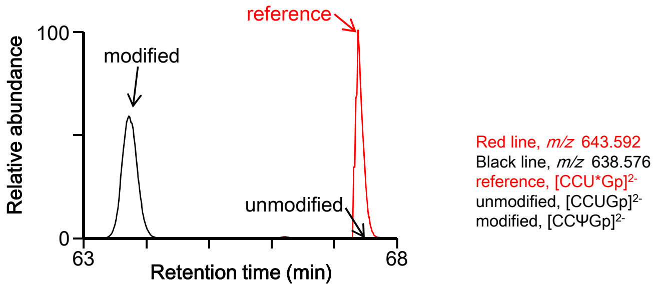

B

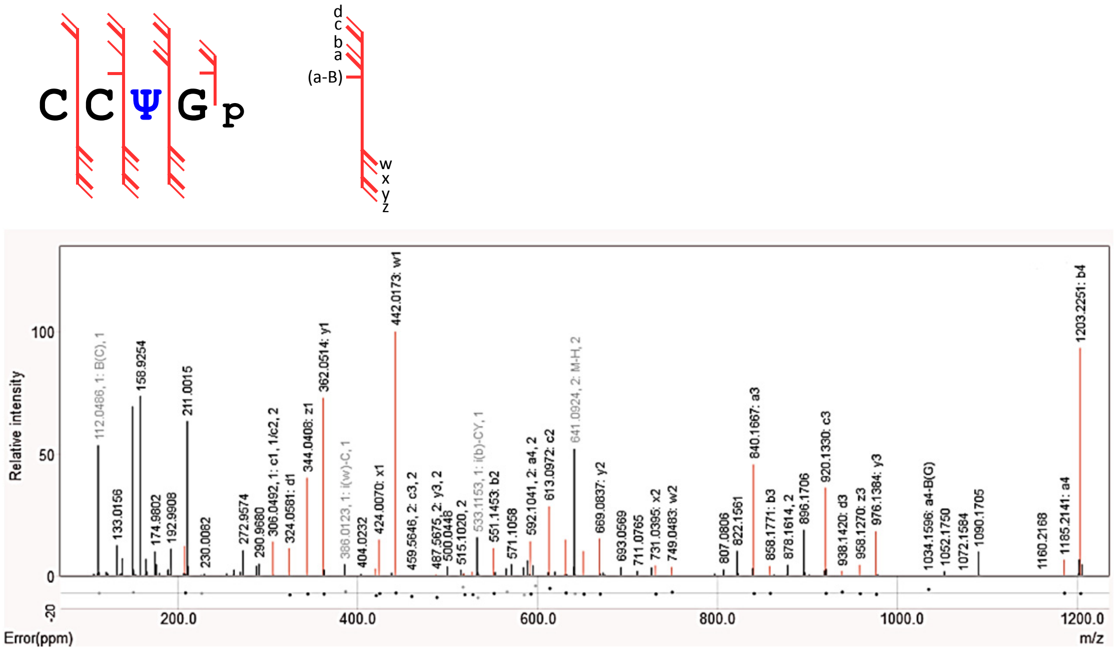

C

#	a-B	(a-B)2-	a	a2-	b	b2-	c	c2-	d	d2-	seq.	w	w2-	x	x2-	y	y2-	z	z2-	#
5'											OH									5'
1	113.0244	56.0086	226.0802	112.5365	244.0908	121.5418	306.047	152.5196	324.057	161.5249	C									4
2	420.0783	209.5355	533.1341	266.0634	551.1446	275.0687	613.1	306.047	631.111	315.0518	C	1056.1025	527.548	1038.0919	518.542	976.136	487.564	958.126	478.5592	3
3	---	---	840.1657	419.5792	858.1762	428.5845	920.132	459.562	938.143	468.5676	Ψ	749.0486	374.0207	731.0381	365.0154	669.082	334.0375	651.072	325.0322	2
4	1034.164	516.578	1185.213	592.103	1203.224	601.1082					G	442.0171	220.5049	424.0065	211.4996	362.051	180.5217	344.04	171.5164	1
3'											P									3'

Supplementary Figure S5-6. Taoka *et al*

A

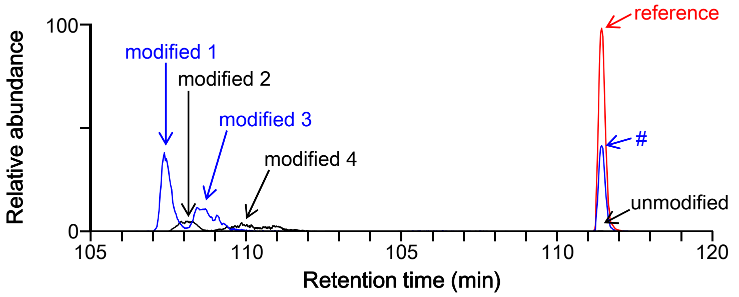

Red line, *m/z* 1771.425
Black line, *m/z* 1769.418
Blue line, *m/z* 1772.221
reference, [AUCUCAACCUAUUCUCAAACUUUAAAU*Gp]$^{5-}$
unmodified, [AUCUCAACCUAUUCUCAAACUUUAAAUGp]$^{5-}$
modified 1, [AUC**Um**CAACCΨAΨΨCUCAAACUUΨAAAUGp]$^{5-}$
modified 2, [AUCUCAACCΨAΨΨCUCAAACUUΨAAAUGp]$^{5-}$
modified 3, [AUC**Um**CAACCUAΨΨCUCAAACUUΨAAAUGp]$^{5-}$
modified 4, [AUCUCAACCUAΨΨCUCAAACUUΨAAAUGp]$^{5-}$
#, one of the isotope signal of [AUCUCAACCUAUUCUCAAACUUUAAAU*Gp]$^{5-}$ can be seen.

B

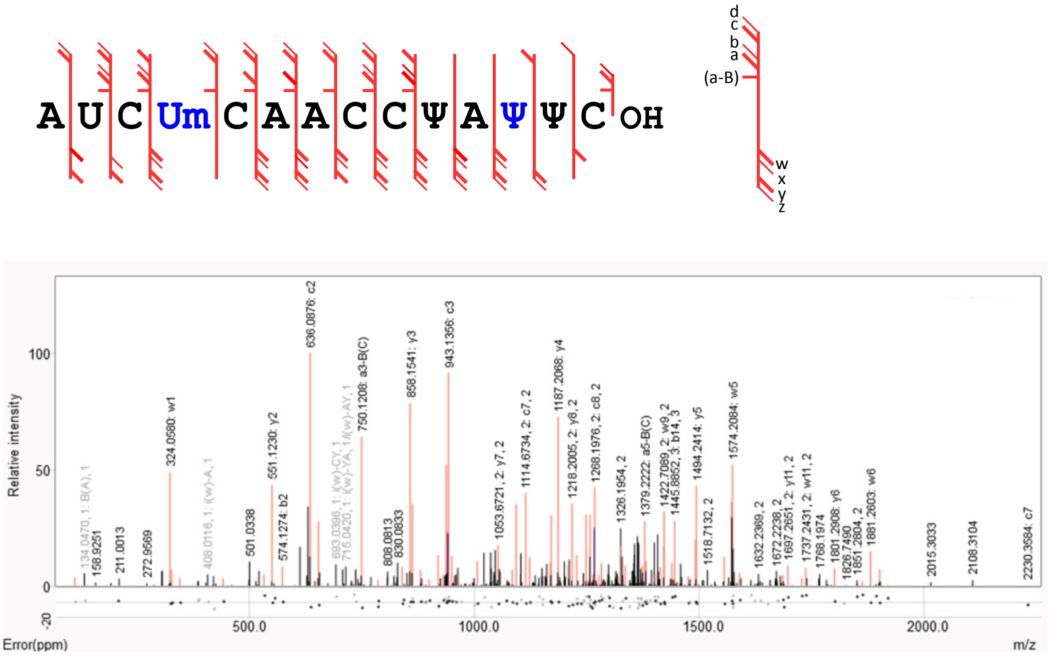

C

#	a-B	(a-B)$^{2-}$	(a-B)$^{3-}$	a	a^{2-}	a^{3-}	b	b^{2-}	b^{3-}	c	c^{2-}	c^{3-}	d	d^{2-}	d^{3-}	seq.
5'																OH
1	113.024	56.009	37.003	248.079	123.536	82.022	266.090	132.541	88.025	328.045	163.519	108.677	346.056	172.524	114.680	A
2	442.077	220.535	146.688	556.117	277.555	184.701	574.127	286.560	190.704	636.083	317.538	211.356	654.094	326.543	217.360	U
3	750.115	374.554	249.367	863.171	431.082	287.052	881.181	440.087	293.056	943.137	471.065	313.708	961.148	480.070	319.711	C
4	1071.184	535.089	356.390	1185.224	592.108	394.403	1203.235	601.114	400.407	1265.190	632.092	421.059	1283.201	641.097	427.062	Um
5	1379.222	689.107	459.069	1492.278	745.635	496.755	1510.289	754.641	502.758	1572.244	785.619	523.410	1590.255	794.624	529.413	C
6	1686.276	842.634	561.421	1821.331	910.162	606.439	1839.341	919.167	612.442	1901.297	950.145	633.094	1919.307	959.150	639.098	A
7	2015.329	1007.161	671.105	2150.383	1074.688	716.123	2168.394	1083.693	722.126	2230.349	1114.671	742.778	2248.360	1123.676	748.782	A
8	2344.381	1171.687	780.789	2457.437	1228.215	818.474	2475.447	1237.220	824.478	2537.403	1268.198	845.130	2555.414	1277.203	851.133	C
9	2651.435	1325.214	883.140	2764.491	1381.742	920.825	2782.501	1390.747	926.829	2844.457	1421.725	947.481	2862.468	1430.730	953.484	C
10	---	---	---	3071.522	1535.258	1023.169	3089.533	1544.263	1029.173	3151.489	1575.241	1049.825	3169.499	1584.246	1055.828	Ψ
11	3265.520	1632.257	1087.835	3400.575	1699.784	1132.853	3418.585	1708.789	1138.857	3480.541	1739.767	1159.509	3498.552	1748.772	1165.512	A
12	---	---	---	3707.606	1853.300	1235.197	3725.617	1862.305	1241.201	3787.573	1893.283	1261.853	3805.583	1902.288	1267.856	Ψ
13	---	---	---	4014.638	2006.815	1337.541	4032.649	2015.821	1343.545	4094.604	2046.799	1364.197	4112.615	2055.804	1370.200	Ψ
14	4208.636	2103.814	1402.207	4321.692	2160.342	1439.892	4339.702	2169.348	1445.896							C
3'																OH

seq.	w	w^{2-}	w^{3-}	x	x^{2-}	x^{3-}	y	y^{2-}	y^{3-}	z	z^{2-}	z^{3-}	#
OH													5'
A													14
U	4090.616	2044.804	1362.867	4072.606	2035.799	1356.864	4010.650	2004.821	1336.212	3992.639	1995.816	1330.208	13
C	3782.578	1890.786	1260.188	3764.568	1881.780	1254.184	3702.612	1850.802	1233.533	3684.601	1841.797	1227.529	12
Um	3475.524	1737.259	1157.837	3457.514	1728.253	1151.833	3395.558	1697.275	1131.181	3377.548	1688.270	1125.178	11
C	3153.471	1576.232	1050.486	3135.460	1567.227	1044.482	3073.505	1536.249	1023.830	3055.494	1527.243	1017.827	10
A	2846.417	1422.705	948.134	2828.407	1413.700	942.131	2766.451	1382.722	921.479	2748.440	1373.717	915.475	9
A	2517.365	1258.179	838.450	2499.354	1249.173	832.447	2437.398	1218.196	811.795	2419.388	1209.190	805.791	8
C	2188.312	1093.652	728.766	2170.302	1084.647	722.762	2108.346	1053.669	702.110	2090.335	1044.664	696.107	7
C	1881.258	940.126	626.415	1863.248	931.120	620.411	1801.292	900.142	599.759	1783.281	891.137	593.756	6
Ψ	1574.204	786.599	524.063	1556.194	777.593	518.060	1494.238	746.615	497.408	1476.228	737.610	491.404	5
A	1267.173	633.083	421.719	1249.162	624.078	415.716	1187.207	593.100	395.064	1169.196	584.094	389.061	4
Ψ	938.120	468.557	312.035	920.110	459.551	306.032	858.154	428.573	285.380	840.143	419.568	279.376	3
Ψ	631.089	315.041	209.691	613.078	306.035	203.688	551.122	275.058	183.036	533.112	266.052	177.032	2
C	324.057	161.525	107.348	306.047	152.520	101.344	244.091	121.542	80.692	226.080	112.537	74.689	1
OH													3'

Supplementary Figure S5-7. Taoka *et al*

A

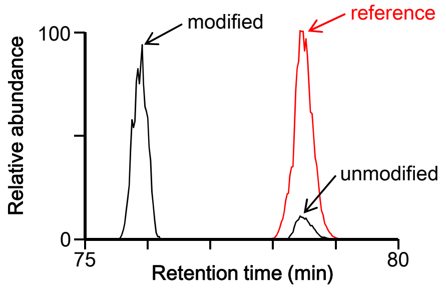

Red line, *m/z* 1448.482
Black line, *m/z* 1445.138
reference, [UUCUCUUUUCUUU*Gp]$^{3-}$
unmodified, [UUCUCUUUUCUUUGp]$^{3-}$
modified, [UUCUCUUΨUCUUUGp]$^{3-}$

B

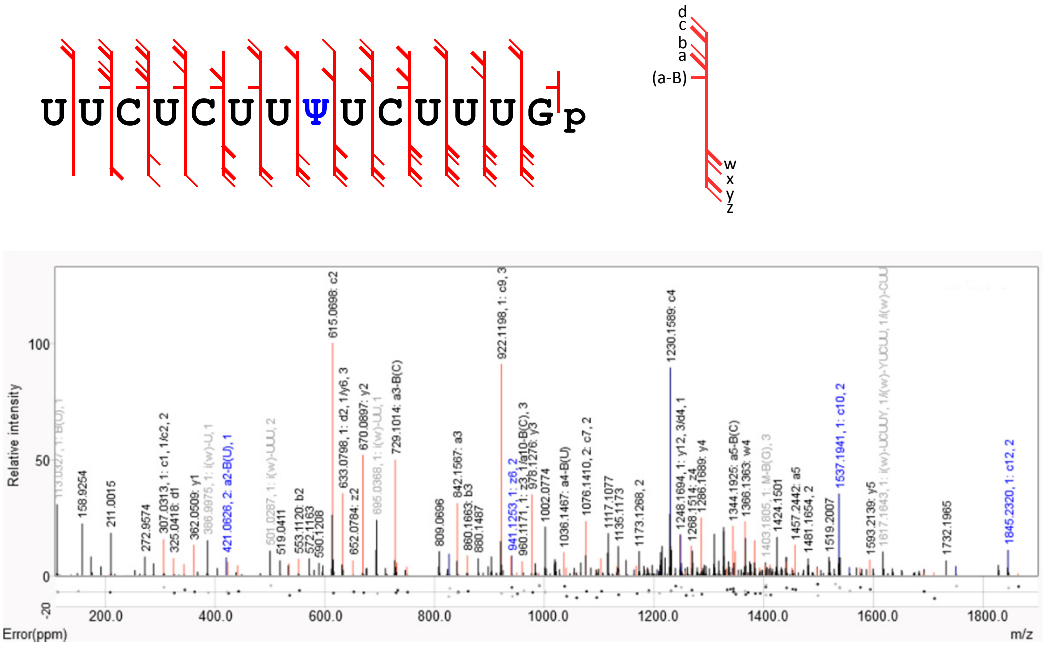

C

#	a-B	$(a-B)^{2-}$	$(a-B)^{3-}$	a	a^{2-}	a^{3-}	b	b^{2-}	b^{3-}	c	c^{2-}	c^{3-}	d	d^{2-}	d^{3-}	seq.	w	w^{2-}	w^{3-}	x	x^{2-}	x^{3-}	y	y^{2-}	y^{3-}	z	z^{2-}	z^{3-}	#
5'																OH													5'
1	113.024	56.009	37.003	227.064	113.029	75.017	245.075	122.034	81.020	307.031	153.012	101.672	325.041	162.017	107.676	U													14
2	421.062	210.028	139.683	535.102	267.047	177.696	553.113	276.053	183.699	615.068	307.031	204.351	633.079	316.036	210.355	U	4134.513	2066.753	1377.500	4116.502	2057.748	1371.496	4054.547	2026.770	1350.844	4036.536	2017.764	1344.841	13
3	729.100	364.046	242.362	842.156	420.574	280.047	860.167	429.580	286.051	922.122	460.558	306.703	940.133	469.563	312.706	C	3826.475	1912.734	1274.820	3808.465	1903.729	1268.817	3746.509	1872.751	1248.165	3728.498	1863.746	1242.161	12
4	1036.154	517.573	344.713	1150.194	574.593	382.726	1168.204	583.599	388.730	1230.160	614.576	409.382	1248.171	623.582	415.385	U	3519.421	1759.207	1172.469	3501.411	1750.202	1166.465	3439.455	1719.224	1145.814	3421.444	1710.219	1139.810	11
5	1344.192	671.592	447.392	1457.248	728.120	485.078	1475.258	737.126	491.081	1537.214	768.103	511.733	1555.225	777.109	517.737	C	3211.383	1605.188	1069.790	3193.373	1596.183	1063.786	3131.417	1565.205	1043.134	3113.407	1556.200	1037.131	10
6	1651.246	825.119	549.744	1765.286	882.139	587.757	1783.296	891.144	593.761	1845.252	922.122	614.412	1863.262	931.128	620.416	U	2904.330	1451.661	967.438	2886.319	1442.656	961.435	2824.363	1411.678	940.783	2806.353	1402.673	934.779	9
7	1959.284	979.138	652.423	2073.323	1036.158	690.436	2091.334	1045.163	696.440	2153.290	1076.141	717.092	2171.300	1085.147	723.095	U	2596.292	1297.642	864.759	2578.281	1288.637	858.756	2516.325	1257.659	838.104	2498.315	1248.654	832.100	8
8	---	---	---	2380.355	1189.674	792.780	2398.366	1198.679	798.784	2460.321	1229.657	819.436	2478.332	1238.662	825.439	Ψ	2288.254	1143.623	762.080	2270.243	1134.618	756.076	2208.288	1103.640	735.424	2190.277	1094.635	729.421	7
9	2574.353	1286.673	857.446	2688.393	1343.693	895.459	2706.403	1352.698	901.463	2768.359	1383.676	922.115	2786.370	1392.681	928.118	U	1981.222	990.108	659.736	1963.212	981.102	653.732	1901.256	950.124	633.081	1883.245	941.119	627.077	6
10	2882.391	1440.692	960.125	2995.447	1497.220	997.811	3013.457	1506.225	1003.814	3075.413	1537.203	1024.466	3093.424	1546.208	1030.470	C	1673.185	836.089	557.057	1655.174	827.083	551.053	1593.218	796.105	530.401	1575.208	787.100	524.398	5
11	3189.445	1594.219	1062.477	3303.485	1651.239	1100.490	3321.495	1660.244	1106.494	3383.451	1691.222	1127.145	3401.461	1700.227	1133.149	U	1366.131	682.562	454.705	1348.120	673.556	448.702	1286.164	642.579	428.050	1268.154	633.573	422.046	4
12	3497.483	1748.238	1165.156	3611.522	1805.258	1203.169	3629.533	1814.263	1209.173	3691.489	1845.241	1229.825	3709.499	1854.246	1235.828	U	1058.093	528.543	352.026	1040.082	519.538	346.023	978.126	488.560	325.371	960.116	479.554	319.367	3
13	3805.520	1902.257	1267.835	3919.560	1959.277	1305.849	3937.571	1968.282	1311.852	3999.527	1999.260	1332.504	4017.537	2008.265	1338.508	U	750.055	374.524	249.347	732.044	365.519	243.343	670.089	334.541	222.691	652.078	325.535	216.688	2
14	4113.558	2056.276	1370.515	4264.608	2131.800	1420.864	4282.618	2140.806	1426.868							G	442.017	220.505	146.668	424.007	211.500	140.664	362.051	180.522	120.012	344.040	171.516	114.009	1
3'																P													3'

A

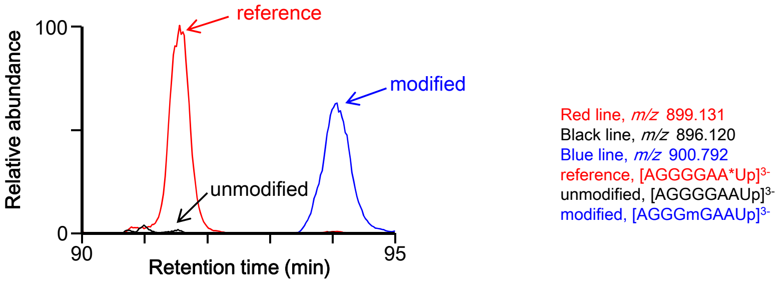

B

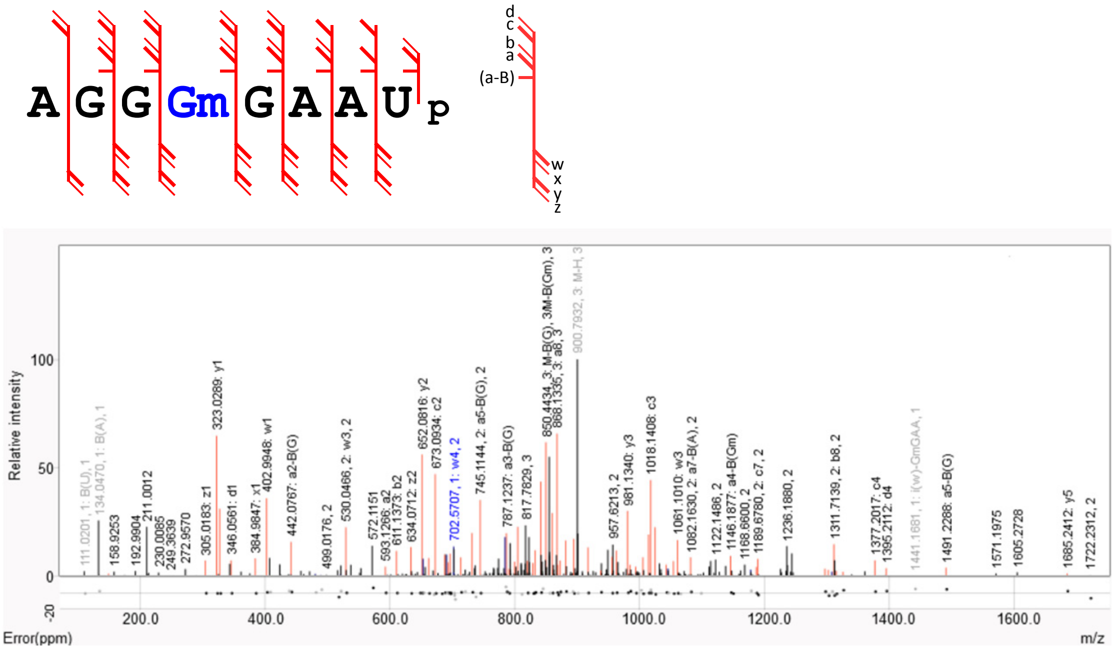

C

#	a-B	(a-B)2-	(a-B)3-	a	a2-	a3-	b	b2-	b3-	c	c2-	c3-	d	d2-	d3-	seq.	w	w2-	w3-	x	x2-	x3-	y	y2-	y3-	z	z2-	z3-	#
5'																OH													5'
1	113.024	56.009	37.003	248.079	123.536	82.022	266.090	132.541	88.025	**328.045**	163.519	108.677	**346.056**	172.524	114.680	A													8
2	**442.077**	220.535	146.688	**593.126**	296.060	197.037	**611.137**	305.065	203.041	**673.093**	336.043	223.693	**691.103**	345.048	229.696	G	2455.305	1227.149	817.764	2437.295	1218.144	811.760	2375.339	**1187.166**	**791.108**	2357.329	**1178.161**	**785.105**	7
3	**787.124**	393.059	261.703	**938.174**	468.583	312.053	**956.184**	477.589	318.057	**1018.140**	508.566	338.709	1036.151	**517.572**	344.712	G	2110.258	**1054.625**	**702.748**	2092.247	**1045.620**	**696.744**	2030.292	**1014.642**	676.092	2012.281	**1005.637**	**670.089**	6
4	**1146.188**	**572.590**	381.391	**1297.237**	**648.115**	431.741	**1315.247**	657.120	437.744	**1377.203**	**688.098**	458.396	**1395.214**	**697.103**	464.400	Gm	1765.211	**882.102**	587.732	1747.200	**873.096**	581.729	**1685.244**	**842.118**	561.077	1667.234	**833.113**	555.073	5
5	**1491.235**	**745.114**	496.407	1642.284	**820.639**	546.757	1660.295	**829.644**	552.760	**1722.251**	**860.622**	573.412	1740.261	**869.627**	579.416	G	1406.147	**702.570**	468.044	1388.137	**693.565**	462.041	**1326.181**	**662.587**	441.389	**1308.171**	**653.582**	435.385	4
6	1836.282	**917.638**	611.423	1971.337	**985.165**	656.441	1989.347	**994.170**	662.444	2051.303	**1025.148**	**683.096**	2069.314	1034.153	689.100	A	**1061.100**	**530.046**	353.029	**1043.089**	**521.041**	347.025	**981.134**	**490.063**	326.373	**963.123**	**481.058**	320.370	3
7	2165.335	**1082.164**	721.107	2300.389	**1149.691**	766.125	2318.400	1158.696	772.129	2380.356	**1189.674**	792.780	2398.366	1198.680	798.784	A	**732.047**	365.520	243.344	**714.037**	356.515	237.341	**652.081**	325.537	216.689	**634.071**	316.532	210.685	2
8	2494.387	1246.690	**830.791**	2606.415	**1302.704**	**868.133**	2624.425	**1311.709**	874.137							U	**402.995**	200.994	133.660	**384.984**	191.989	127.657	**323.029**	161.011	107.005	**305.018**	152.005	101.001	1
3'																P													3'

Supplementary Figure S5-9. Taoka *et al*

A

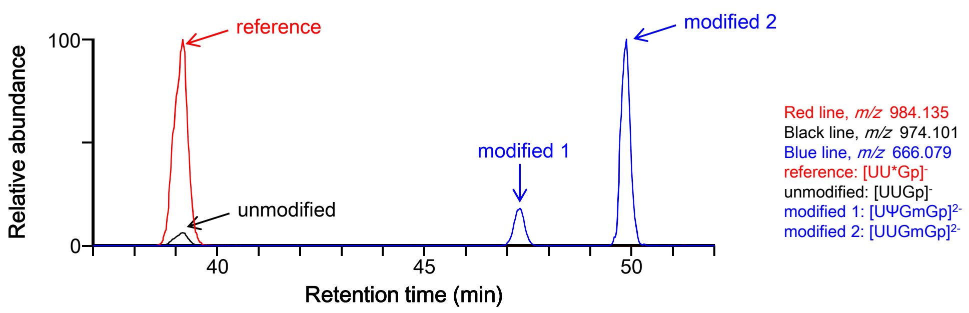

B

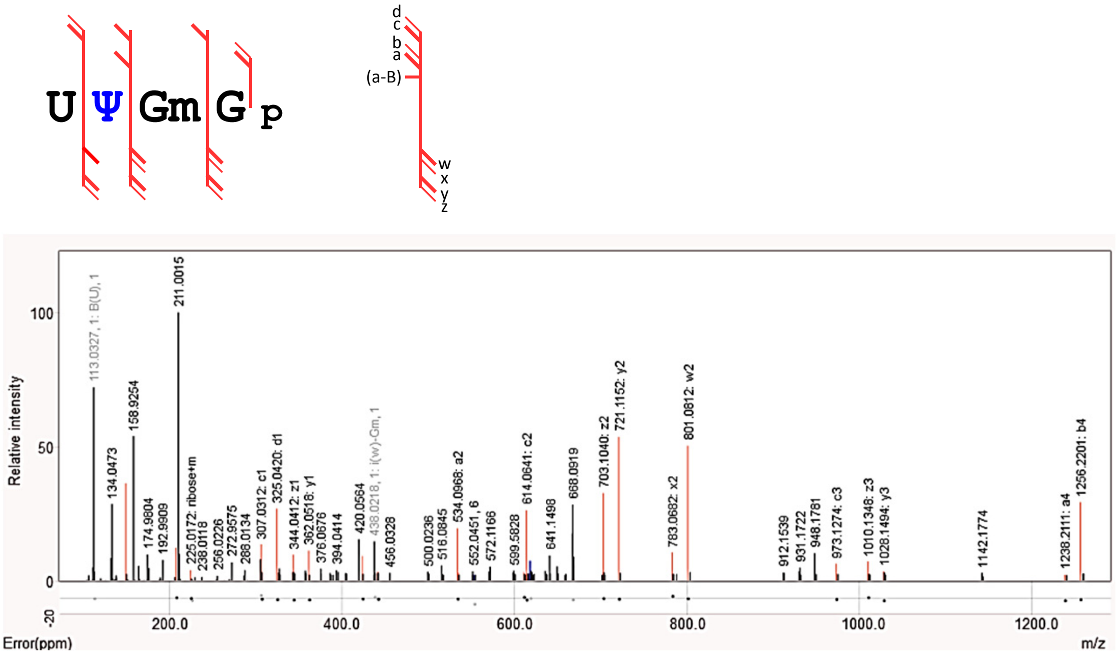

C

#	a-B	(a-B)$^{2-}$	a	a^{2-}	b	b^{2-}	c	c^{2-}	d	d^{2-}	seq.	w	w^{2-}	x	x^{2-}	y	y^{2-}	z	z^{2-}	#
5'											OH									5'
1	113.024	56.009	227.064	113.029	245.075	122.034	**307.031**	153.012	**325.041**	162.017	U									4
2	---	---	**534.096**	266.544	552.106	275.550	**614.062**	306.527	632.073	315.533	Ψ	1108.112	**553.552**	1090.101	544.547	**1028.145**	513.569	**1010.135**	504.564	3
3	742.110	370.551	893.159	446.076	911.170	455.081	**973.125**	486.059	991.136	495.064	Gm	**801.080**	400.036	**783.070**	391.031	**721.114**	360.053	**703.103**	351.048	2
4	1087.157	543.075	**1238.206**	**618.600**	**1256.217**	627.605					G	**442.017**	220.505	**424.007**	211.500	**362.051**	180.522	**344.040**	171.516	1
3'											P									3'

Supplementary Figure S6. Taoka *et al*

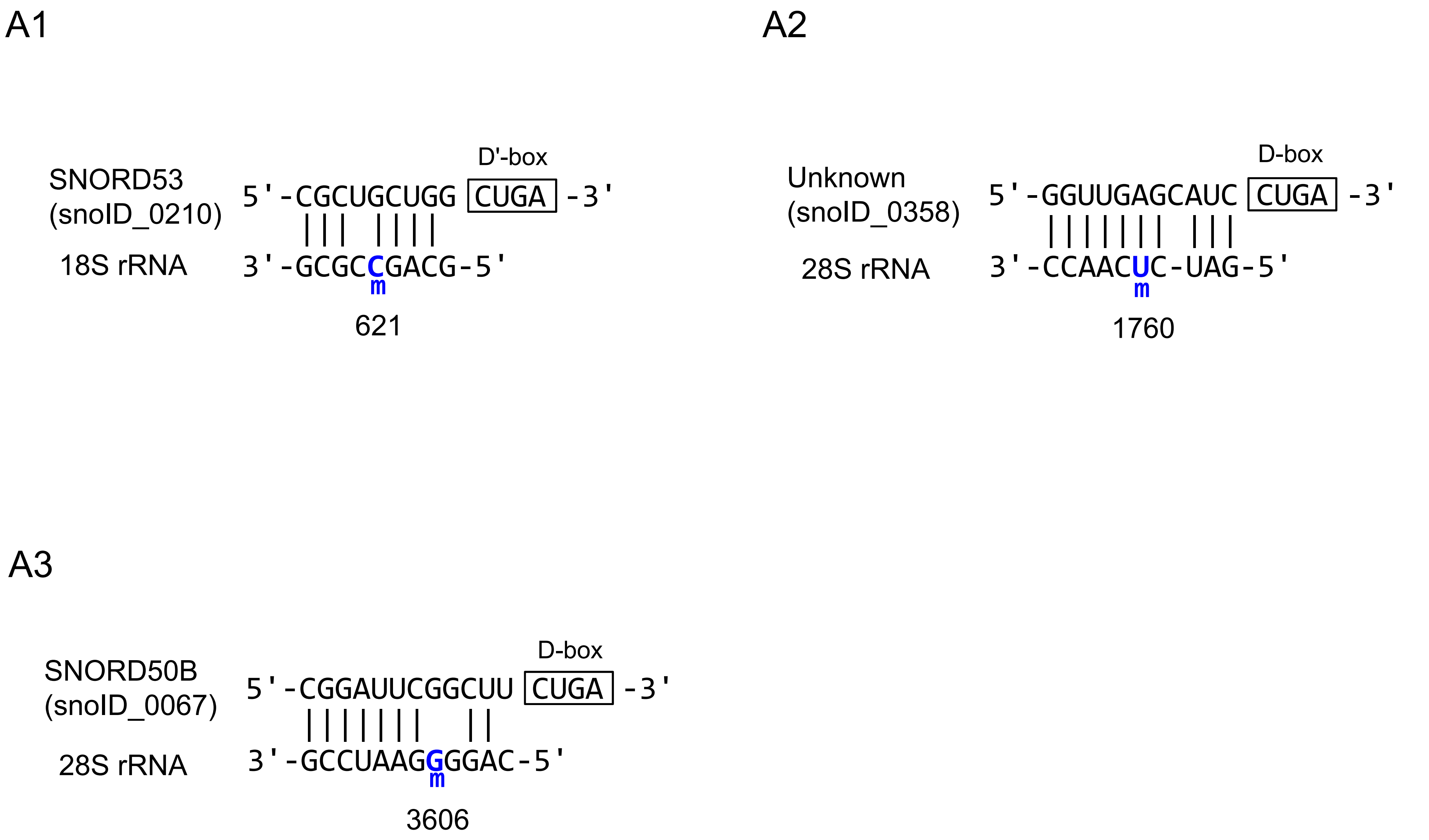

Supplementary Figure S6. Taoka *et al -continued*

B1

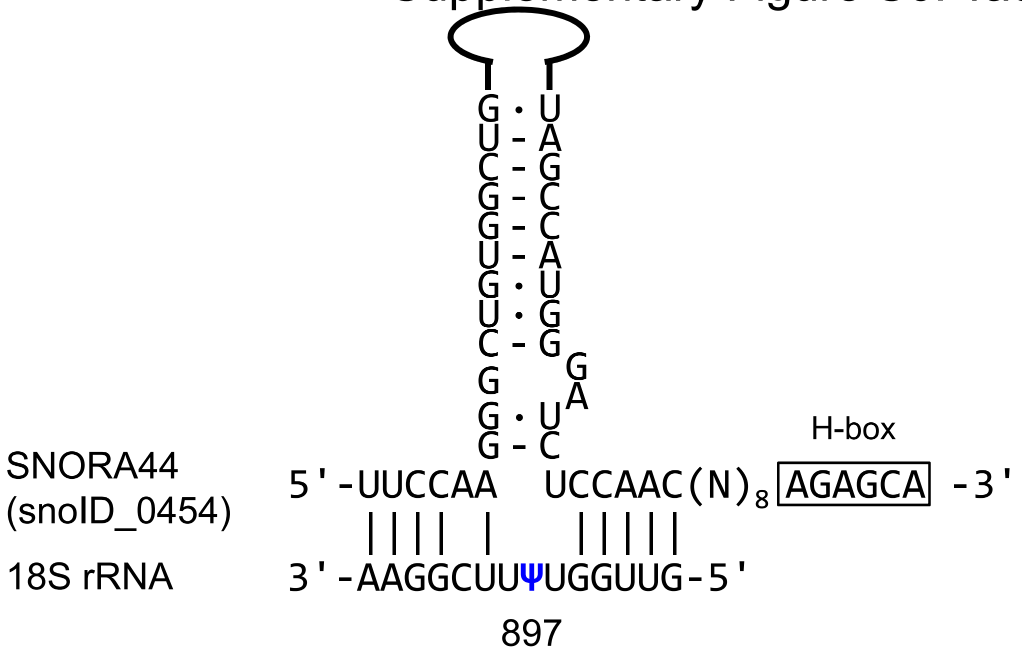

B2

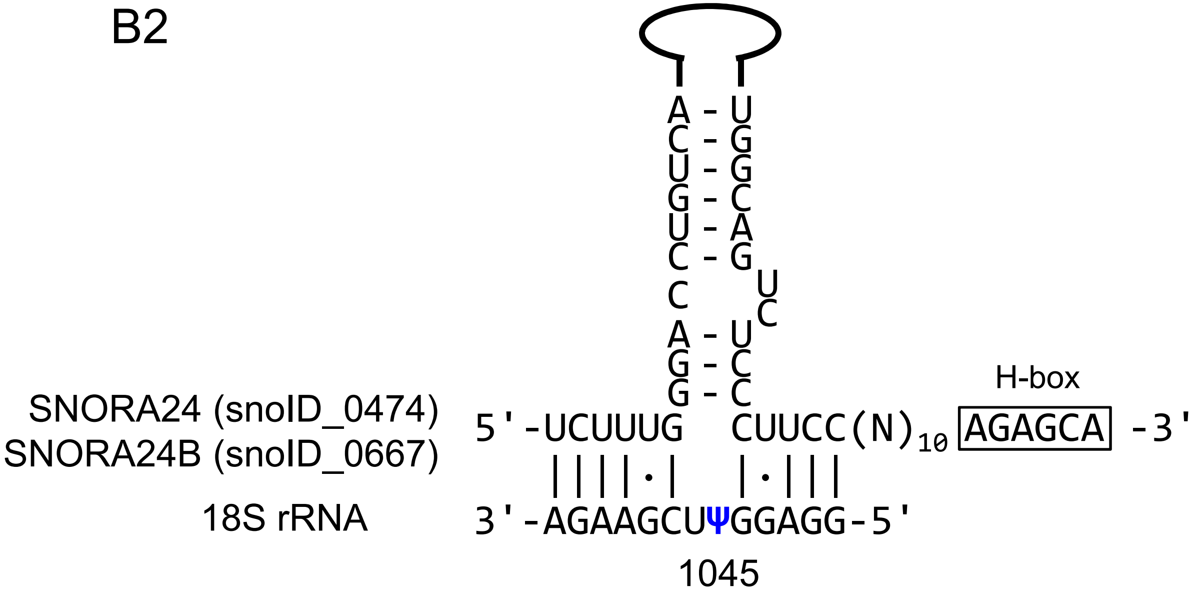

B3

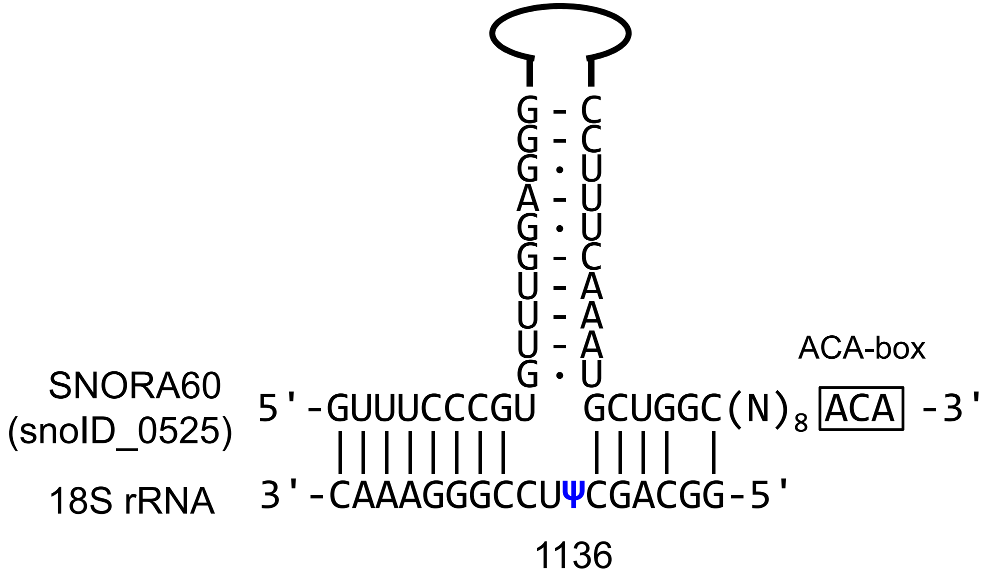
G - C
G - C
G · U
A - U
G · U
G - C
U - A
U - A
U - A
G · U
ACA-box
SNORA60
(snoID_0525)
5'-GUUUCCCGU GCUGGC(N)8 ACA -3'
18S rRNA
3'-CAAAGGGCCUΨCGACGG-5'
1136

B4

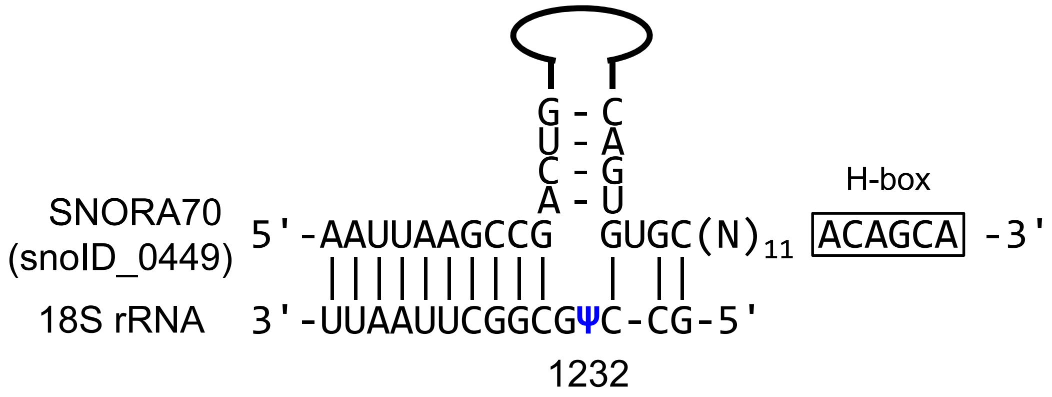
G - C
U - A
C - G
A - U
H-box
SNORA70
(snoID_0449)
5'-AAUUAAGCCG GUGC(N)11 ACAGCA -3'
18S rRNA
3'-UUAAUUCGGCGΨC-CG-5'
1232

B5

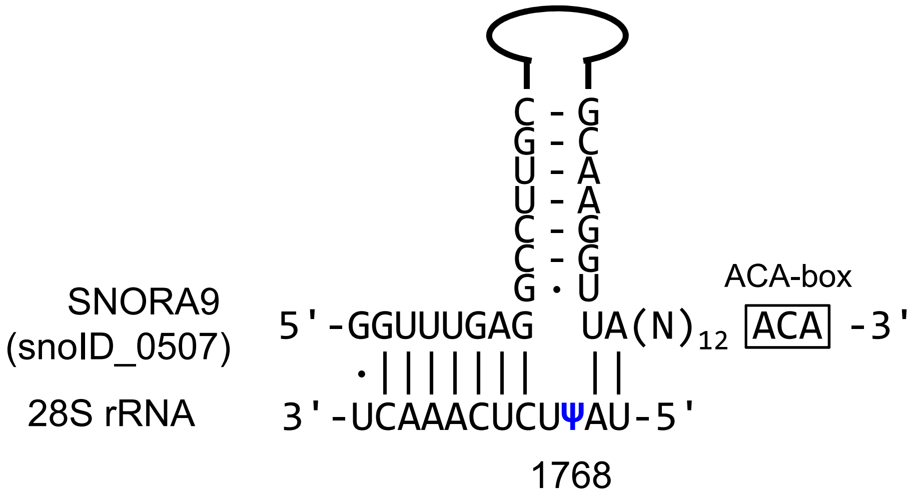
ACA-box
SNORA9
(snoID_0507)
5'-GGUUUGAG UA(N)12 ACA -3'
28S rRNA
3'-UCAAACUCUΨAU-5'
1768

B6

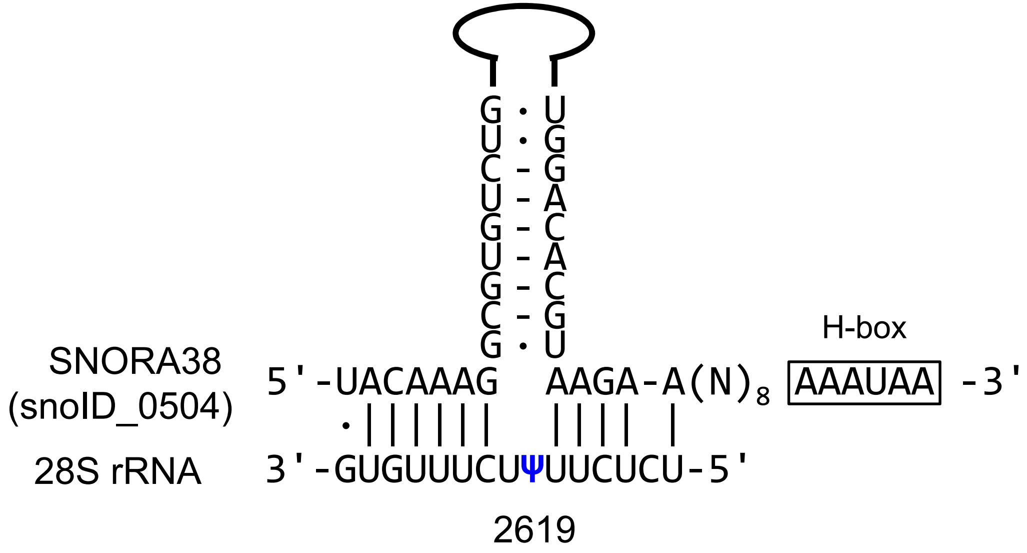
H-box
SNORA38
(snoID_0504)
5'-UACAAAG AAGA-A(N)8 AAAUAA -3'
28S rRNA
3'-GUGUUUCUΨUUCUCU-5'
2619

B7

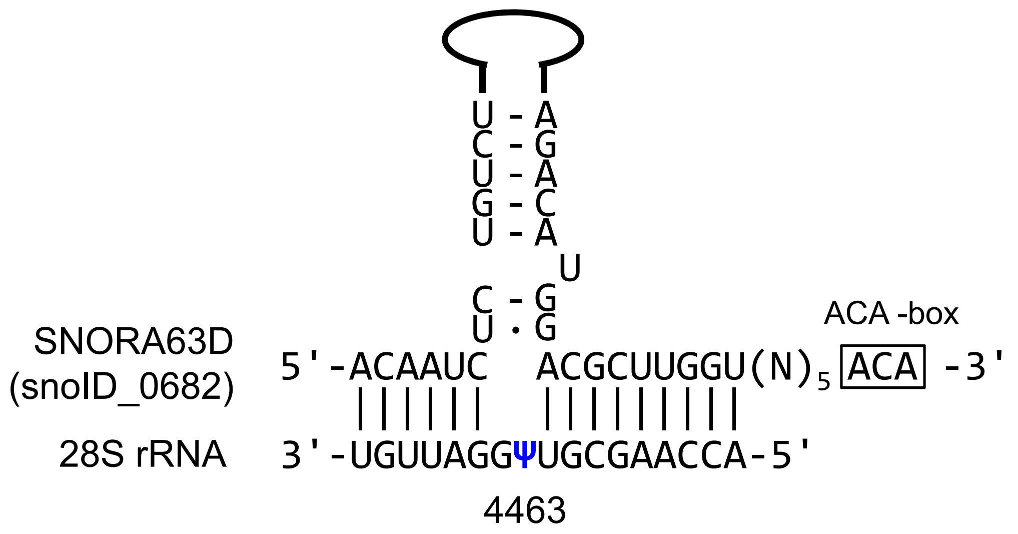
U - A
C - G
U - A
G - C
U - A
U
C - G
U · G
ACA -box
SNORA63D
(snoID_0682)
5'-ACAAUC ACGCUUGGU(N)5 ACA -3'
28S rRNA
3'-UGUUAGGΨUGCGAACCA-5'
4463

Supplementary Figure S7. Taoka *et al*

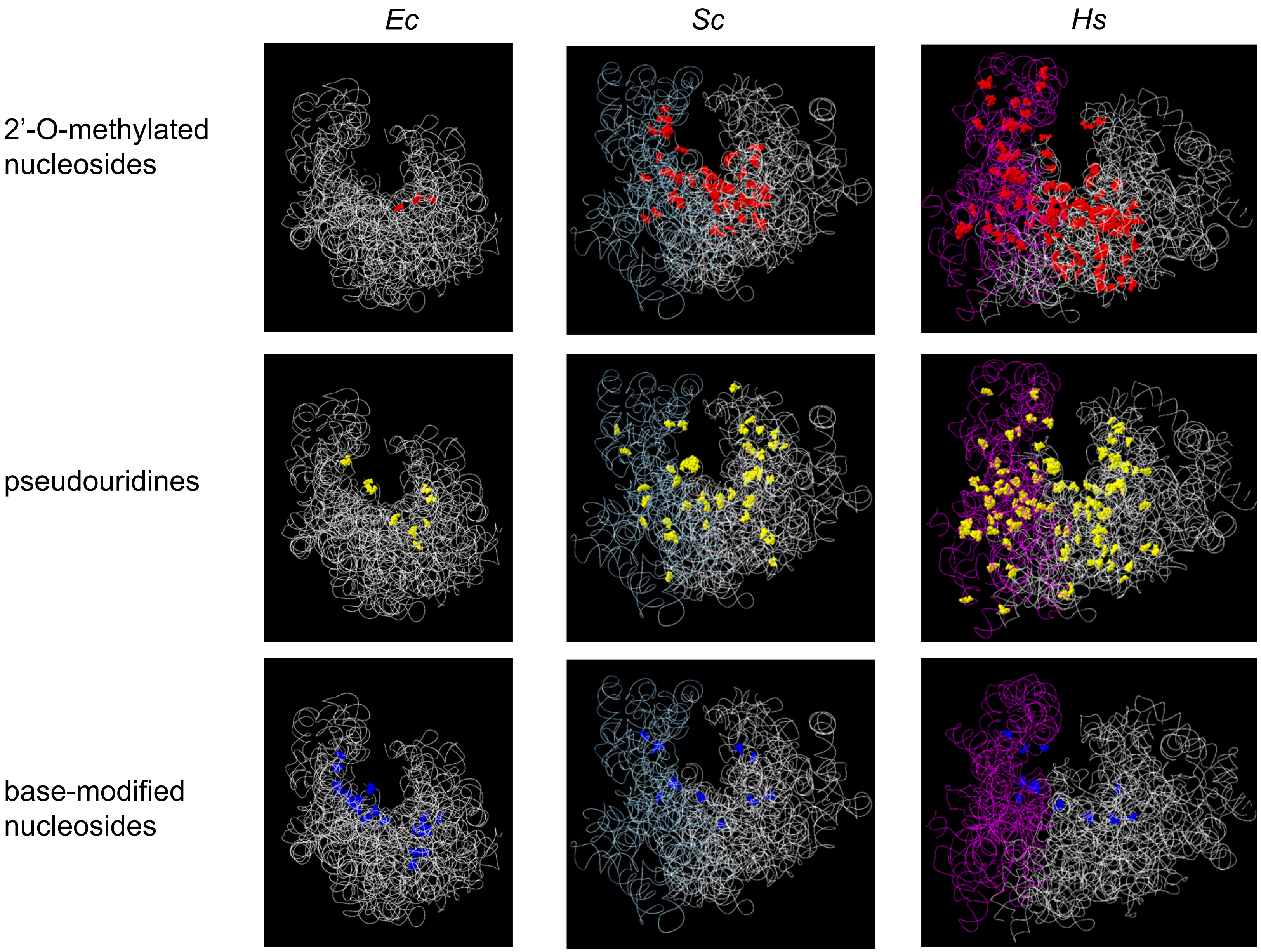

Supplementary Figure S8. Taoka *et al*

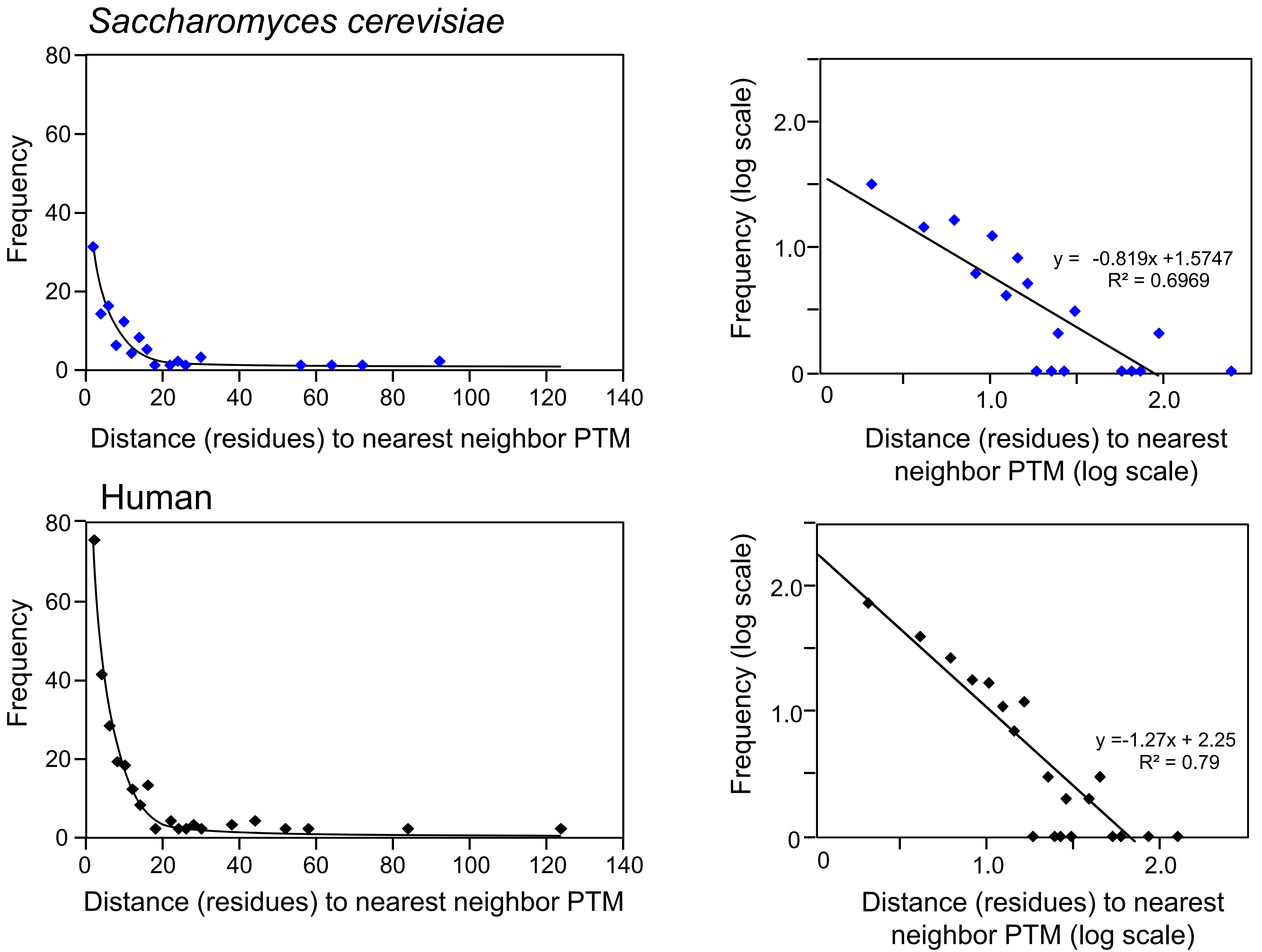

Supplement: Supplementary Data [file gky811_supplemental_files.zip › Human_rRNA_PTM_SuppleFig_Rev.pdf]
